# Determination of extended substrate specificity of the MALT1 as a strategy for the design of potent substrates and activity-based probes

**DOI:** 10.1038/s41598-018-34476-7

**Published:** 2018-10-30

**Authors:** Paulina Kasperkiewicz, Sonia Kołt, Tomasz Janiszewski, Katarzyna Groborz, Marcin Poręba, Scott J. Snipas, Guy S. Salvesen, Marcin Drąg

**Affiliations:** 1 Department of Bioorganic Chemistry, Faculty of Chemistry, Wroclaw University of Science and Technology, Wyb. Wyspianskiego 27, 50-370 Wroclaw, Poland; 2 NCI-designated Cancer Center, Sanford Burnham Prebys Medical Discovery Institute, La Jolla, CA 92037 USA

## Abstract

Mucosa-associated lymphoid tissue lymphoma translocation protein 1 (MALT1) belongs to the CD clan of cysteine proteases. MALT1 is a unique enzyme among this clan because it recognizes the basic amino acid arginine in the P1 pocket. Previous studies carried out with natural amino acids revealed the substrate specificity of the P4-P1 pockets of MALT1 but have provided only limited information about the catalytic preferences of this enzyme. In this study, we exploited Hybrid Combinatorial Substrate Library and Internally Quenched Fluorescence substrate technologies to interrogate the extended substrate specificity profile of the S5-S2’ active site pockets using unnatural amino acids. This strategy resulted in the design of a peptide-based fluorogenic substrate, which exhibited significant activity toward MALT1. Subsequently, the substrate sequence was further utilized to develop potent, irreversible activity-based probes.

## Introduction

Mucosa-associated lymphoid tissue lymphoma translocation protein 1 (MALT1), together with caspases, separase, legumain and gingipain, belongs to the CD clan of cysteine proteases. MALT1 is a component of the CBA (CARMA - Bcl-10 - MALT1)^1^ proteolytic complex that cleaves and inactivates NF-ƙB signaling pathway inhibitors such as TNFAIP3/A20^2^, Bcl-10^3^, RELB^4^ and CYLD^5,6^. Therefore, MALT1 indirectly activates NF-ƙB and, consequently, is a potentially important therapeutic target for the treatment of MALT lymphoma and metastasis^7^.

Numerous processes are regulated by proteases with a degree of specificity that is often determined by the amino acid sequence of the substrate. Similar to caspases, MALT1 contains a paracaspase domain^8^; however, the MALT1 S1 pocket can bind only arginine, while caspases recognize aspartic acid, and to a lesser extent glutamic acid^9,10^. This preferential hydrolysis is a function of the conformation of the surrounding active site pockets within the three-dimensional structure of the enzyme. These subsites are named according to their location in the active site groove. Subsites in the enzyme are designated by the letter “S”, and the corresponding peptide residues in the substrate by the letter “P” in nonprime Sx subsite, according to the nomenclature of Schechter and Berger^11^. The substrate specificity of the MALT1 S4-S2 pockets has been determined using a positional scanning synthetic combinatorial library (PS-SCL)^12^ that consisted of natural amino acids only. Our previous studies, however, have demonstrated that the incorporation of unnatural amino acids in substrate libraries may result in the identification of highly potent and selective peptide sequences^13–17^. We also raised the question of whether the substrate specificity of the prime pockets of MALT1 would be similar to that of caspases, which preferentially recognize small aliphatic amino acids.

The main objective of this work was to determine the substrate specificity of MALT1 at the P5-P2’ positions using tailored combinatorial and individual substrate peptide libraries. Based on the preferences of MALT1 at the P5-P1 positions, optimal substrates and activity-based probes were designed, synthesized and biochemically characterized. These small chemical tools could provide information about enzyme activity status in cells, physiological fluids and lysates, which could subsequently be useful in studying the role of MALT1 in physiology and disease.

## Results

### MALT1 catalytic preferences in the S4-S2 pockets

Z-VRPR-FMK, which was originally designed as a plant metacaspase inhibitor^3^, is the most frequently used MALT1 inhibitor and binds covalently to the MALT1 active site. Mepazine is another inhibitor of MALT1, but functions in a noncovalent and reversible manner, limiting its usefulness in studying the enzyme^18^. Another irreversible inhibitor of MALT1, MI-2, has a high IC_50_ value (5.84 μM) but exhibits activity toward caspases -3, -8 and -9^19^. Hachmann *et al*. described the first tetrapeptidic MALT1-activity-based probe, which was characterized by medium potency (k_obs_/I = 9, 300 M^−1^s^−1^)^20^. A year later, Xin *et al*. developed some additional new probes (derivatives of MI-2); however, the IC_50_ values for these probes were relatively high^21^. Therefore, more precise determination of the MALT1 substrate specificity was required for optimal substrate and inhibitor design. Hachmann *et al*. identified some interesting features of MALT1 substrate specificity using natural amino acids^12^. At the P4 position, MALT1 has narrow specificity, preferring leucine over other amino acids. However, at P3 and P2, the substrate preferences are broad^12^, thus providing increased opportunity for the identification of new substrates and probes for MALT1 based on highly diverse structures. To determine whether extending the chemical space in S2-S4 would deliver higher catalytic rates, we used Hybrid Combinatorial Substrate Libraries (HyCoSuL) with defined P1-Arg and containing both natural and unnatural amino acids at fixed sites^13,17^.

**Figure 1:**
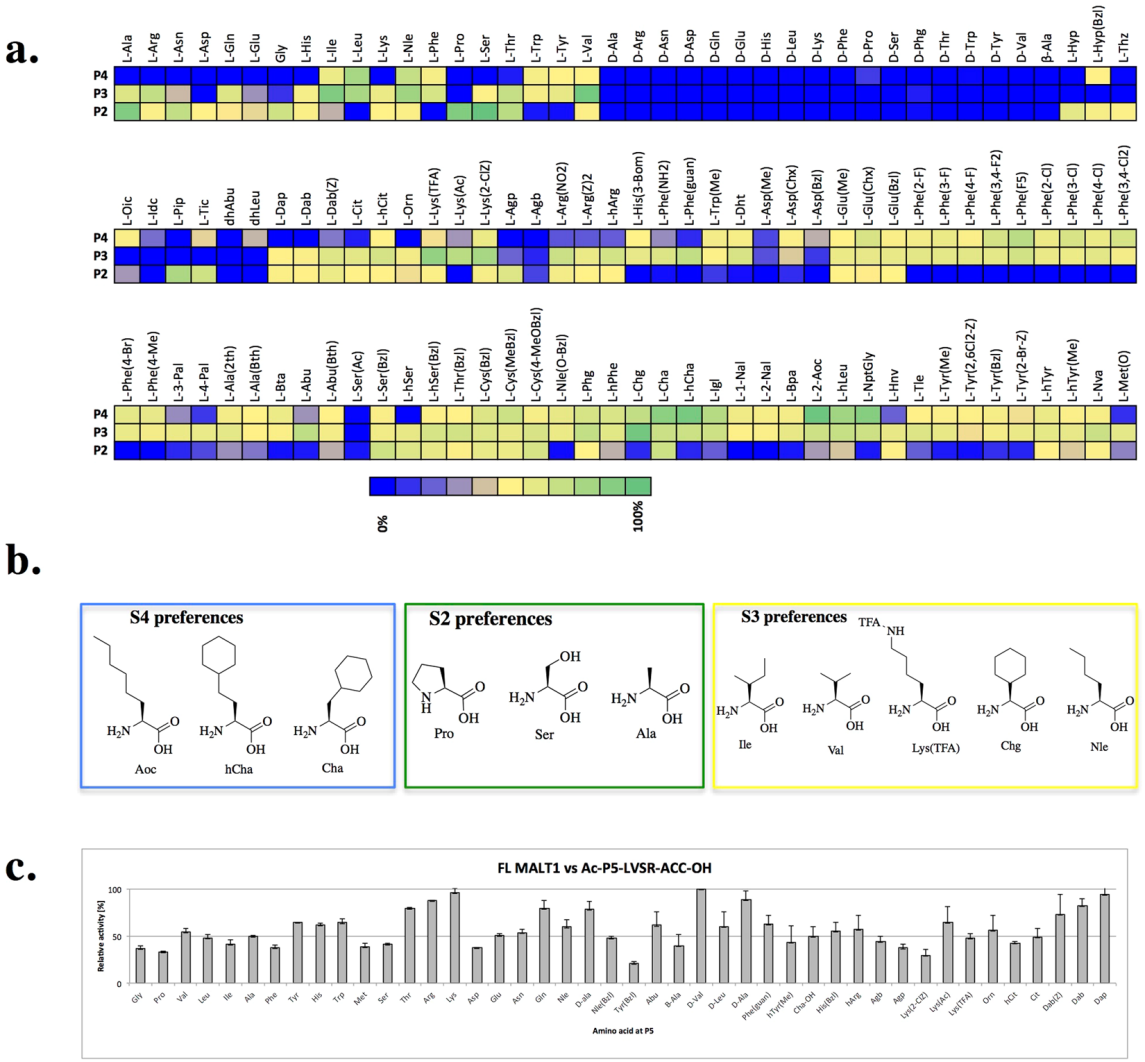
MALT1 S5-S2 substrate preferences. Panel a. Substrate preferences of the MALT1 S4-S2 pockets; 100 μM library component was treated with 130 nM active site-titrated MALT1 in assay buffer containing 50 mM HEPES, 100 mM NaCl, 0.9 M sodium citrate and 10 mM DTT at pH 7.5. Panel b. Structures of the preferred amino acid residues at the P4-P2 positions of MALT1. Panel c. MALT1 P5 specificity. Specificity profile of MALT1 was determined with the library of general structure Ac-P5-LVSR-ACC; 100 nM MALT1 was incubated at 37 °C for 10 minutes, followed by the addition of 1 µM substrate. Relative fluorescence units were measured over time at excitation/emission wavelengths of 355/460 nm for at least 10 minutes. The data represent averages of three separate measurements, and error bars represent standard deviations.

We observed that the S2 pocket can accommodate small amino acids, such as serine (relative activity, 100%) and alanine (85%), as well as proline (84%) and proline derivatives such as piperidine (61%). D-amino acids and large, bulky, hydrophobic structures, including phenylalanine, tyrosine and tryptophan, were not recognized by this pocket. Similarly, among the pool of unnatural amino acids, phenylalanine derivatives such as charged analogues (Phe(NH_2_), Phe(guan)) or derivatives bearing additional halogen (Phe(4-Cl)) or methyl groups (Phe(Me)) did not fit in this pocket. Surprisingly, of the diverse set of amino acids used in the screening, the natural sequences were better recognized at this site compared with unnatural sequences (Fig. 1a,b).

The MALT1 S3 subsite exhibited a preference for aliphatic amino acids, including both branched (valine, 98%; isoleucine, 91%; leucine, 66%) or unbranched (norleucine, 72%) structures. Interestingly, unnatural cyclohexylglycine (100%) was as well-recognized as valine. In contrast to the charged amino acid lysine (26%), lysine derivatives (Lys(TFA), 79%; Lys(Ac), 56%; Lys(2-ClZ), 67%) were well tolerated in the S3 pocket. Similar to the binding at the S2 subsite, D-amino acids did not fit into this pocket, and unlike S2, proline and proline derivatives (Oic, Tic) were not recognized at S3 (Fig. 1a,b).

At the P4 position, unnatural amino acids were preferred over natural amino acids. The most preferred amino acids were Cha and hCha (80% and 92%, respectively), 2-Aoc (100%), hLeu (69%) and NptGly (85%). The most active natural amino acid was leucine (65%).

Our observations regarding the natural amino acid preferences of P4-P2 are consistent with the data reported by Hachmann *et al*.^12^, where the LVSR sequence was identified as the best MALT1 substrate (Fig. 1a,b).

### P5 library design, synthesis and screening

Hachmann *et al*. observed that MALT1 substrates containing five amino acids exhibited higher activity than substrates with four amino acids in the peptide backbone^12^; however, the preferences of the MALT1 S5 pocket have never been explored. Therefore, we designed a P5 library containing natural and unnatural amino acids based on the specificity of the S4-S2 pockets. We used the most promising structure as a lead structure, which contained a serine at P2; the remaining positions were as follows: leucine at P4, valine at P3 and arginine at P1 (Ac-P5-LVSR-ACC, ACC – 7-aminocoumarin-4-acetic acid) (Fig. 1c).

Using this tailored library, we demonstrated that MALT1 tolerates all the investigated amino acids at the S5 pocket (Fig. 1c). L- and D- isomers of aliphatic amino acids, similar to basic residues, were preferred over other amino acids; however, the difference was subtle. The least active amino acid residue at the P5 position was found to be tyrosine with a benzyl group (Tyr(Bzl)). Given the broad P5 specificity, we concluded that the S5 pocket region of the enzyme may not be shaped as a pocket with restricted specificity but rather have the shape of a surface^12^. To better understand the MALT1 S5 kinetics and amino acid preferences, we calculated the k_cat_/K_m_ values for selected structures. Our calculations indicated that D-valine (k_cat_/K_m_ = 7, 940 ± 240 s^−1^M^−1^), Abu (k_cat_/K_m_ = 8, 760 ± 50 s^−1^M^−1^) and alanine (k_cat_/K_m_ = 8, 850 ± 930 s^−1^M^−1^) as well as arginine (k_cat_/K_m_ = 6, 040 ± 18 s^−1^M^−1^) were preferred by MALT1 at the S5 pocket (Table 1).

**Table 1:** Preferences of MALT1 at the P5 position (kinetic calculations).

Sequence	k_cat_ s^−1^	K_m_, μM	k_cat_/K_m_, s^−1^M^−1^
Ac-*D-*Val-LVSR-ACC	0.15 ± 0.012	19.25 ± 2.50	7, 940 ± 243
Ac-*D-*Ala-LVSR-ACC	0.076 ± 0.034	24.35 ± 6.97	3, 300 ± 214
Ac-Thr-LVSR-ACC	0.12 ± 0.0050	22.41 ± 9.94	5, 420 ± 1082
Ac-Arg-LVSR-ACC	0.12 ± 0.0003	19.06 ± 0.05	6, 040 ± 18
Ac-Lys-LVSR-ACC	0.12 ± 0.0010	25.51 ± 3.56	4, 770 ± 760
Ac-Ala-LVSR-ACC	0.089 ± 0.033	9.45 ± 2.46	8, 847 ± 926
Ac-Abu-LVSR-ACC	0.19 ± 0.0295	22.15 ± 0.01	8, 760 ± 50

### Design, synthesis and kinetic analysis of individual substrates

**Figure 2:**
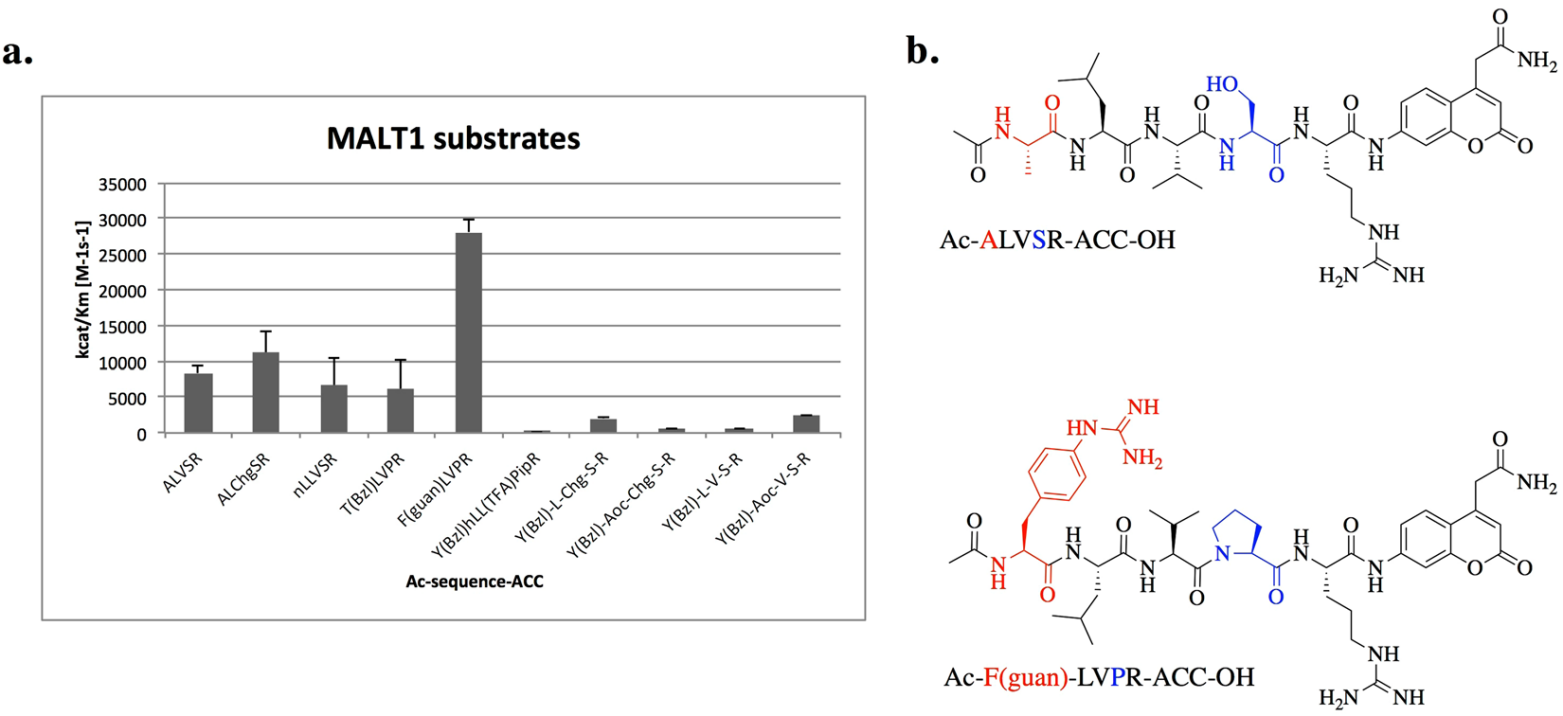
MALT1 substrate activity. Panel a. k_cat_/K_m_ values for MALT1 substrates compared with that of the reference substrate (ALVSR). MALT1 was incubated at 37 °C for 30 minutes and added to the wells containing the substrate at varying concentrations. Relative fluorescence units were measured over time at excitation/emission wavelengths of 355/460 nm for at least 10 minutes. The data represent averages of three separate measurements, and error bars represent standard deviations. Panel b. Structure of our best (lower panel) and the reference (upper panel) MALT1 fluorogenic substrate.

In accordance with the identified MALT1 preferences in the P5-P2 positions, we designed and synthesized a set of defined substrates. We then performed substrate screening and determined the activity of MALT1 towards the most promising substrates. The substrate with Tyr(Bzl) at the P5 position was used as a control (Fig. 2a,b). As expected, the increase in activity against substrates with P3-Chg was low; therefore, we used valine in further experiments. MALT1 was more active toward the substrate Ac-Y(Bzl)-L-Chg-S-R-ACC-OH than toward Ac-Y(Bzl)-Aoc-Chg-S-R-ACC-OH, confirming the P4 library screening results. However, the enzyme was less active toward Ac-Y(Bzl)-L-V-S-R-ACC-OH than toward Ac-Y(Bzl)-Aoc-V-S-R-ACC-OH, indicating strong cooperativity in the enzyme pocket. Therefore, we replaced serine with proline in one of the substrates (Ac-Phe(guan)-LVPR-ACC-OH). Surprisingly this modification led to an increase in MALT1 activity toward the new substrate to 28,000 s^−1^M^−1^, making the new substrate the most active MALT1 substrate compared with the best known substrate (Ac-ALVSR-ACC-OH).

### Design, synthesis and kinetics of the MALT1-activity-based probe

**Figure 3:**
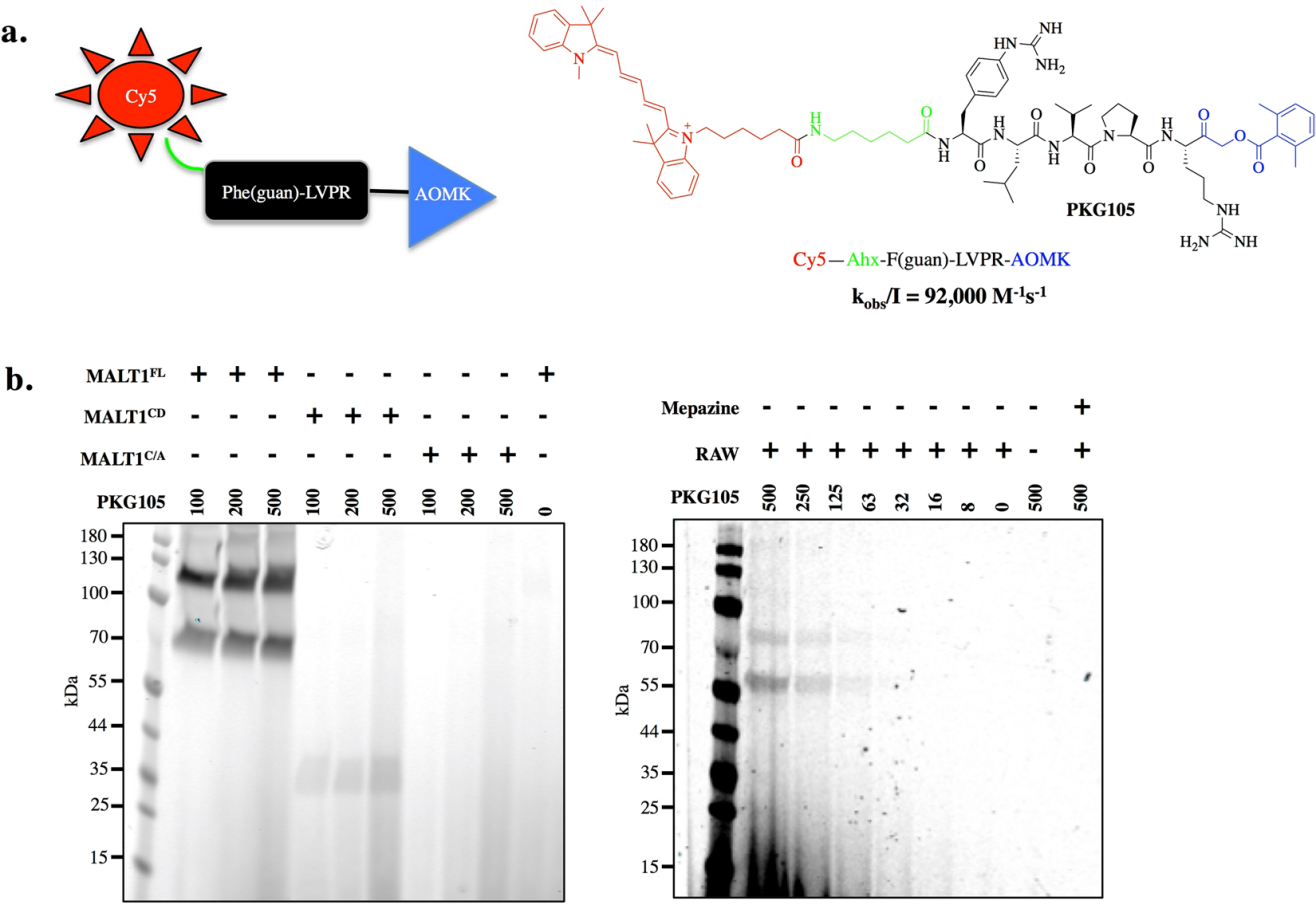
Probe to detect active MALT1. Panel a. Structure of the MALT1 probe (PKG105). Panel b. RAW 264.7 cell lysates were incubated with PKG105 at various concentrations. As a control, lysates were pretreated with mepazine, followed by incubation with probe. Lysates that were not treated with mepazine were also used. SDS-PAGE was performed at 165 V. Fluorescence was scanned using the 700 nm channel (LI-COR). Gels are representative of at least two replicates.

In this study, we designed an activity-based probe that contains a new peptide sequence (Phe(guan)-LVPR), labeled with the fluorescent cyanine derivative Cy5 and equipped with an AOMK (acyloxymethyl keton) warhead that binds covalently at the active site of cysteine proteases (Fig. 3a). The k_obs_/I value (92,000 s^−1^M^−1^) for PKG105 is approximately 10 times higher than the value (9,300 s^−1^M^−1^) reported for the best MALT1 inhibitor in the literature^20^. Therefore, we next tested the utility of PKG105 in *in vitro* assays by treating full-length MALT1 (MALT1^FL^), the MALT1 catalytic domain (MALT1^CD^) and a MALT1 catalytic mutant (MALT1^C/A^) with three different concentrations of the probe. We detected enzyme activity for both MALT1^FL^ and MALT1^CD^, while no labeling was observed for the MALT1^C/A^ mutant, indicating that the probe is bound at the active site (Fig. 3b). MALT1^FL^ in the absence of probe was evaluated as a negative control, where RAW 264.7 cell lysates were incubated with PKG105. We also preincubated RAW 264.7 cell lysates with mepazine (MALT1 inhibitor), followed by addition of the probe. Mepazine inhibited MALT1, and therefore, the PKG105 probe was not bound to the enzyme. Using this tool, we detected active MALT1 in RAW 264.7 cell lysates, demonstrating that PKG105 is capable of selectively detecting MALT1 activity in cell lysates (Fig. 3).

### Design and synthesis of P1’ and P2’ libraries

**Figure 4:**
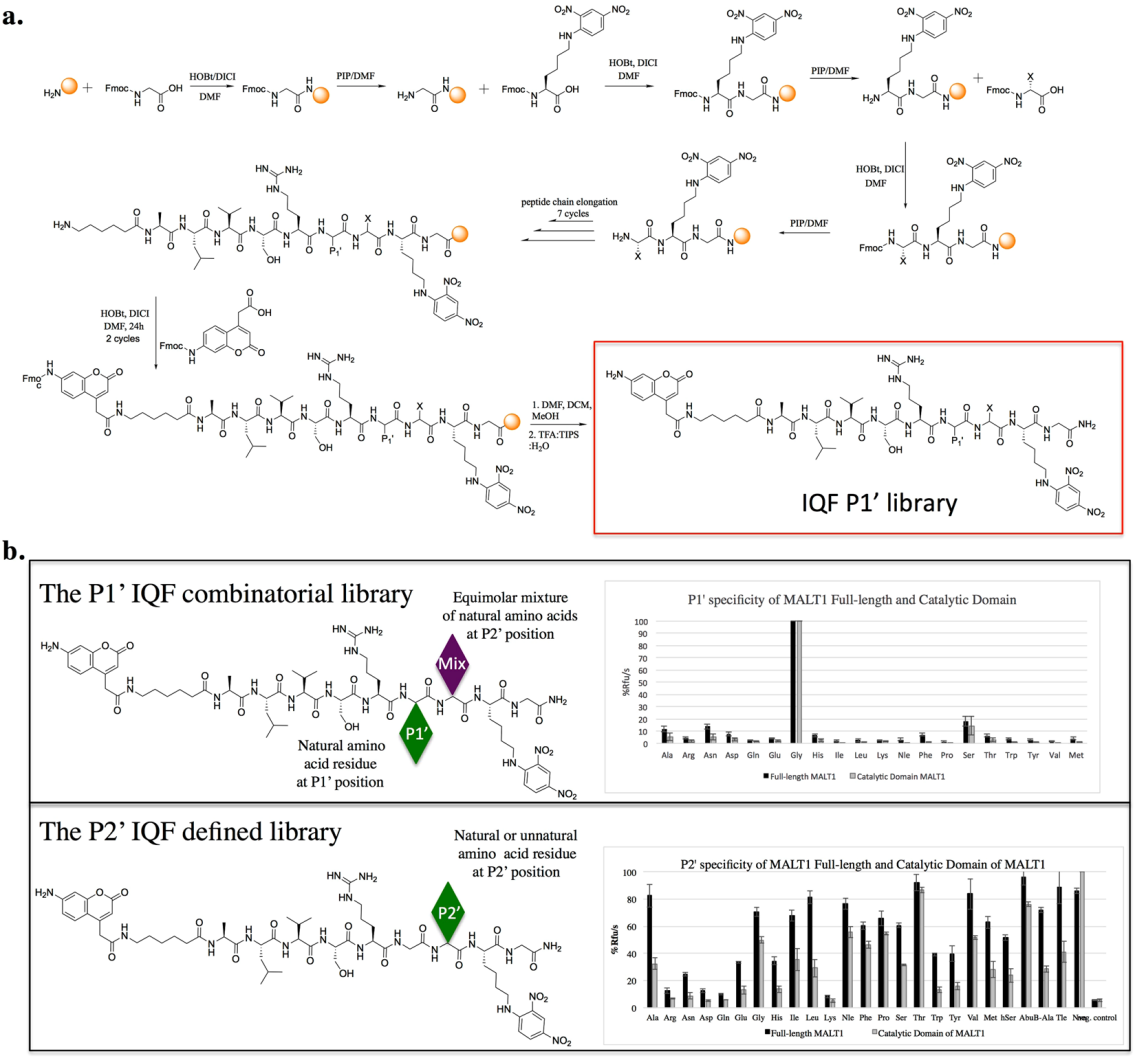
Catalytic preferences of MALT1 at the S1’-S2’ pockets. Panel a. General scheme of P1’ library synthesis. Panel b. Prime site libraries and MALT1 specificity profile. MALT1^CD^ (160/120 nM for libraries/substrate screening, respectively) and MALT^FL^ (80 nM) were incubated at 37 °C for 30 minutes in assay buffer, added to substrates (2 μM/1 μM library/defined substrate, respectively), and fluorescence emission was measured for at least 10 minutes (excitation/emission at 355/460 nm). The data represent averages of three separate measurements, and error bars represent standard deviations.

With the knowledge of the nonprime MALT1 cleavage specificity, it was imperative to define the catalytic preferences of this enzyme at the carboxylic side of hydrolyzed bond (prime sites). To date, the prime site substrate specificity of MALT1 has not been explored; therefore, we next aimed to determine the preferences of the prime binding pocket of this protease. To examine the P1’ preferences, we designed a library with the general structure H_2_N-ACC-Ahx-A-L-V-S-R–P1’-Mix-Lys(Dnp)-Gly-NH_2_, where “Mix” is an equimolar mixture of all natural amino acids, except cysteine and methionine, which is replaced with norleucine. P1’ is a natural amino acid residue, and Ahx is a linker that separates the ACC (7-amiocoumarin-4-acetic acid) fluorophore from the MALT1 catalytic groove. We utilized a classic peptide synthesis method (Fig. 4a), and Rink amide resin was used to attach glycine as the first amino acid in the structure. Since the specificity of P2’ was not known, we incorporated an isokinetic mixture at this position to increase the possibility of peptide binding at the active site of MALT1. Next, we incorporated defined, natural amino acids at P1’, followed by peptide elongation. Among the natural amino acids, arginine, serine, valine, leucine, and alanine fit best at the P1-P5 positions (Fig. 4a). Finally, we attached a fluorogenic moiety to ensure fluorescent readout. Accordingly, to determine the specificity of P2’, we synthesized a combinatorial library with the general structure H_2_N-ACC-Ahx-A-L-V-S-R–Mix-P2’-Lys(Dnp)-Gly-NH_2_. We utilized ACC-Lys(Dnp) as a donor-acceptor pair, because of the stronger signal of this pair compared with other donor-acceptor pairs^9^ (Fig. 4a).

Using the P1’ library, we demonstrated that both MALT1^CD^ and MALT1^FL^ are almost entirely limited to glycine at the P1’ position (100%). Serine was also recognized to a small extent (<20%). The full-length enzyme exhibited low affinity (<10%) for almost all natural amino acids at this position; however, the potency was so low that it can be assumed to be a measurement error. MALT1^CD^ was limited to glycine and serine, while alanine was recognized to a small extent (<5% of the activity) (Fig. 4b). The similar specificity profiles of MALT1^CD^ and MALT1^FL^ indicate that regions extraneous to the catalytic domain do not influence the primary specificity of MALT1. Moreover, given the highly similar specificity profiles of the full-length enzyme and catalytic domain, we concluded that the substrate binds within the enzyme active site that influences the specificity and activity of the enzyme toward the substrate.

**Figure 5:**
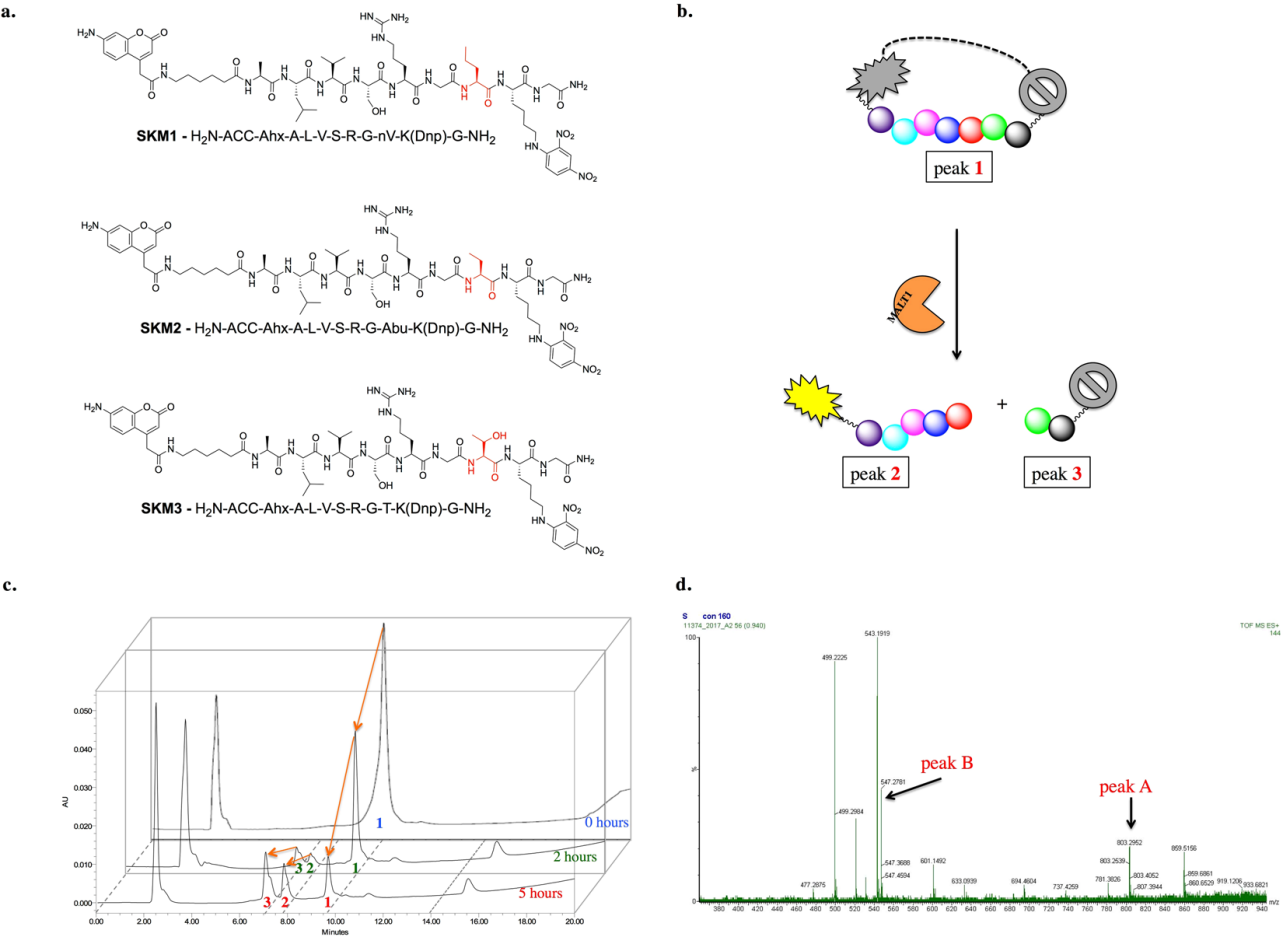
Cleavage site of new IQF MALT1 substrates. Panel a. Structures of the most potent IQF MALT1 substrates. Panel b. Scheme of IQF substrate cleavage by MALT1. Substrate (peak 1) is hydrolyzed at one site and two new peptide derivatives are formed (peak 2 and 3). The first is a pentapeptide with a fluorescent molecule (ACC), while the second is a dipeptide with a quencher molecule. Panel c. Progress of IQF MALT1 substrate hydrolysis over time. Substrate (50 µM) was treated with MALT1 (160 nM) in assay buffer. The experiment was monitored by analytical HPLC for 5 hours. Panel d. MS spectrum of the reaction mixture. Two of the signals are from a pentapeptide derivative and dipeptide derivative formed after IQF substrate hydrolysis by MALT1.

Subsequently, we tested the specificity of the P2’ position and observed slightly different specificity profiles. For MALT1^FL^, alanine (100%) was preferred over threonine (~80%), while for MALT1^CD^, threonine was the best recognized amino acid (100%), followed by alanine (~80%). However, similar to for P1’, we observed similar specificity profiles for MALT1^CD^ and MALT^FL^ (Fig. 4b). In summary, the S2’ pocket has broad specificity, with a preference for aliphatic amino acids (alanine, valine, serine, glycine) or cyclic amino acids (proline). Since P1’ was limited to glycine, we did not perform any additional structural modifications. However, to further explore the S2’ pocket, we additionally synthesized a defined library with the general structure H_2_N-ACC-Ahx-ALVSR–G-P2’-K(Dnp)G-NH_2_, where P2’ is a fixed, defined amino acid residue. The natural library was supplemented with a few unnatural aliphatic amino acids with branched (Tle), linear (Abu, Nle), elongated (hSer, Nva) or β-amino acid (β-Ala) residues. We confirmed that both MALT1^FL^ and MALT1^CD^ prefer aliphatic amino acids (MALT1^FL^: Abu > Ala > Thr > Tle; MALT1^CD^: Nva > Thr > Abu) over charged amino acids (arginine, leucine, aspartic acid). We then selected the best substrates (Nva, Abu, threonine) (Fig. 5a) and compared the kinetic parameters of MALT1^FL^ to those of MALT1^CD^. We found that MALT1^CD^ was less catalytically active than MALT1^FL^ (Table 2).

**Table 2:** k_cat_/K_m_ values calculated for IQF substrates against MALT1.

Substrate	k_cat_/K_m_, M^−1^s^−1^	
	MALT1 ^FL^	MALT1 ^CD^
**M1**, H_2_N-ACC-Ahx-A-L-V-S-R–G-**Nva**-K(Dnp)G-OH	4.8 * 10^3^ ± 362	3.5 * 10^3^ ± 165
**M2**, H_2_N-ACC-Ahx-A-L-V-S-R–G-**Abu**-K(Dnp)G-OH	5.5 * 10^3^ ± 291	2.6 * 10^3^ ± 38
**M3**, H_2_N-ACC-Ahx-A-L-V-S-R–G-**Thr**-K(Dnp)G-OH	6.3 * 10^3^ ± 311	2.8 * 10^3^ ± 101

Finally, we compared the specificity of MALT1^FL^ and MALT1^CD^ on both the tailored and the combinatorial P2’ library. We observed similar specificity profiles, demonstrating the utility of combinatorial Internally Quenched Fluorescent (IQF) substrate libraries in investigation of the MALT1 prime site. However, we observed differences in the activity of MALT1^FL^ toward phenylalanine, norleucine and valine. As expected, the tailored structures were more active than the combinatorial libraries.

### Cleavage site

While designing IQF substrates, we expected cleavage of the peptide at one specific site, after the arginine (Fig. 5b). To test this hypothesis, we treated the IQF substrate with MALT1^FL^ and monitored the hydrolytic yield with analytical HPLC at two separate time points. After 2 hours of hydrolysis, two new peaks appeared, and the signal from the intact substrate decreased over time, while the signal from two new products increased over time (up to 5 hours) (Fig. 5c). The peak at the third minute is from buffer components. Therefore, we confirmed that MALT1^FL^ hydrolyzes the new IQF substrate at one site only. Because little was known about the specific cleavage site, we performed mass spectrometry of the reaction mixture. As expected, the mass spectrum demonstrated that cleavage occurred between the P1 and P1’ amino acid residues (Fig. 5d). We did not observe any other peaks that would indicate a different peptide bond hydrolysis site. After hydrolysis, the fluorescence was not quenched, and the intensity of the signal increased, making these substrates valuable tools for MALT1 activity measurements in extracts or cell lysates.

## Discussion

In the present paper, we describe an extended MALT1 specificity profile at the S5-S2’ sites, as well as introduce new IQF substrates and a Cy5-labeled probe for irreversible labeling of MALT1 *in vitro* and in cell lysates. Hachmann *et al*. asked whether MALT1 has generally lower activity than other members of the CD clan or whether the optimal substrate for MALT1 had not been discovered yet^12^. In our opinion, MALT1 is an interesting paracaspase with restricted specificity at the P1 and P1’ positions, where cooperativity of the enzyme pockets plays a key role in investigation of the substrate. Based on the in-depth kinetic analysis, we concluded that, unlike the S1 pocket, the other substrate binding sites are not fully formed and are present in the form of a platform for substrate binding. However, the rate of substrate hydrolysis (k_cat_/K_m_) for the PKGS16 substrate was estimated to be 28,000 M^−1^s^−1^, which is approximately 3.5 times higher than that of the best reported substrate to date.

We herein report the synthesis of new substrates as well as an activity-based probe that contains guanidynyl-phenylalanine in the P5 positon. This probe can efficiently detect MALT1 at a low nanomolar concentration. Xin *et al*. described a set of activity-based probes for MALT1 detection that had a proline residue incorporated at the P2 position, similar to our structure and Z-VRPR-FMK, a covalent and irreversible MALT1 inhibitor^21^. Moreover, this group modified the MI-2 structure in order to obtain a MALT1-activity-based probe labeled with Cy5. Our PKG105 probe detects MALT1, and only one additional band is observed from undefined species.

All reported MALT1 substrates, which have been generated from only natural amino acids, are classic peptides that bind to the five pockets of the enzyme. The fairly strict requirement for glycine at the P1’ position is in an agreement with the hydrolysis sites occurring in natural MALT1 substrates (HOIL, CYLD, RelB, NIK, A20, Regnase^−1^ and RC3H1), where hydrolysis occurs between the P1 arginine and P1’ glycine residues^6^.

The fact that large, bulky amino acid residues are minimally recognized leads us to hypothesize that IQF substrates should be the substrates of choice for MALT1 activity detection, instead of the classic tetrapeptide substrates that incorporate fluorophores at P1’. In summary, in this report, we describe potent substrates and activity-based probes for MALT1 and, for the first time, describe the specificity of the prime pockets of MALT1.

## Methods

### Kinetic assays

All kinetic assays were performed with a Spectra Max Gemini XPS spectrofluorimeter (Molecular Devices) and 96-well Corning plates. The measurement characteristics were as follows: 37 °C, excitation/emission wavelength of 355/460 nm, cutoff of 455 nm, and varying concentrations of substrates and enzyme. The assay buffer was prepared at 23 °C and contained 50 mM HEPES (2-[4-(2-hydroxyethyl)piperazin-1-yl]ethanesulfonic acid), 100 mM NaCl, 0.9 M sodium citrate and 10 mM dithiothreitol (DTT) (pH 7.5). MALT1^FL^ or MALT1^CD^ were preincubated for 30 minutes at 37 °C before being added to wells containing substrate. Next, the increase in fluorescence was recorded over the time. Only the linear portions of the curves were used for calculations. The k_cat_ and K_m_ values were calculated using GraphPad Prism and Microsoft Excel software. All measurements were repeated three times, and the data presented are averages of these replicates.

### Synthesis of activity-based probes

#### (1) Boc-Arg(Z_2_)-AOMK synthesis

First, Boc-Arg(Z_2_)-OH was converted into a bromomethyl ketone (BMK) using a previously described synthetic procedure^22^. A 0.2 M solution of Boc-Arg(Z_2_)-OH (5 mmol, 1 eq (equivalent)) in anhydrous THF (tetrahydrofuran) was stirred in an ice/acetone bath at −10 °C for 20 minutes. Then, 4-methylmorpholine (6.25 mmol, 1.25 eq) and isobutyl chloroformate (5.75 mmol, 1.15 eq) were added. Immediately after adding isobutyl chloroformate, a white precipitate was formed, which indicated anhydride formation. The reaction was carried out at −10 °C for 35 minutes. In a parallel experiment, an ethereal solution of diazomethane was generated according to the Aldrich Technical Bulletin (AL-180) protocol. A solution of mixed anhydrides was then added dropwise to the ethereal diazomethane (16.6–21.4 mmol) at 0 °C. The mixture was vigorously stirred for 30 minutes, after which the ice bath was removed. The reaction was carried out for another hour. Next, 15 mL of a 1:2 solution of HBr (30% wt. in CH_3_COOH), and water was added dropwise to the mixture over a period of 15 minutes. After one hour, the reaction was complete, and the mixture was diluted with ethyl acetate, transferred to a separatory funnel and extracted with saturated aqueous NaHCO_3_ (three times), brine (twice) and water (once). The organic fraction was dried over MgSO_4_ and evaporated under reduced pressure. The product was obtained as a yellow oil and used for further synthesis without purification. The product purity determined by HPLC was >85%, and the overall yield was >80%. Next, the obtained product was transformed to acyloxymethyl ketone by using 1.2 eq of 2,4-dimethylbenzoic acid and 3 eq of KF in DMF. The reaction was carried out for one hour, and the mixture was diluted again with ethyl acetate, transferred to a separatory funnel and extracted with water (once), saturated aqueous NaHCO_3_ (three times) and brine (three times). The organic fraction was dried over MgSO_4_ and evaporated under reduced pressure. The product was obtained as a yellow oil, and the yield of this reaction was >95%.

#### (2) Cy5-Phe(guan)-Leu-Val-Pro-Arg-AOMK (PKG105)

First, the Boc-Phe(guan)-Leu-Val-Pro-OH peptide was synthesized on solid support using 2-chlorotrityl chloride resin and Fmoc-protected amino acids. Initially, 300 mg of 2-chlorotrityl chloride resin (0.48 mmol) was suspended in 4 mL of anhydrous DCM and gently stirred once every 5 minutes for 30 minutes. Next, the DCM was filtered off, and the resin was washed three times with DCM. Then, 3 eq of the Fmoc-*L*-Pro-OH amino acid in anhydrous DCM was preactivated with 3 eq of DIPEA and added to the resin. The suspension was gently agitated. After 4 hours, the mixture was filtered, and the resin was washed two times with DCM and three times with DMF in order to dispose of excess reagents. The Fmoc protecting group was removed using a 20% solution of piperidine in DMF (three cycles: 5, 5, and 25 min). After Fmoc group removal, a ninhydrin test was carried out. Next, the resin was washed six times with DMF, and 3 eq of Fmoc-*L*-Val-OH, preactivated with 3 eq of HOBt (hydroxybenzotriazole) and 3 eq of DICI in DMF, was added to the reaction cartridge. The reaction was carried out for three hours at room temperature. After coupling the second amino acid, the Fmoc protecting group was removed in the same manner, and the two remaining amino acid positions were coupled analogously. After coupling all required amino acids, the resin was washed three times with DMF, three times with DCM, and three times with MeOH then dried over P_2_O_5_ (overnight). The tetrapeptide was cleaved from the resin using mild acidic conditions with a mixture of TFE/AcOH/DCM (%, v/v/v, 10:10:80), thus preserving the Boc protecting group at the *N* terminus of the peptide chain. The liquid product was then mixed with hexane, and the solvents were evaporated under reduced pressure. The obtained peptide was dissolved in an ACN/H_2_O mixture (%, v/v 75:25) and lyophilized. The purity of the obtained compound was confirmed by analytical HPLC, and the molecular weight of the compound was determined by HRMS. Next, Boc-Arg(Z_2_)-AOMK was *N*-terminally de-protected in TFA/DCM (1:1, v/v; with 3% TIPS for 30 min) and coupled with the synthesized peptide to obtain Boc-Phe(guan)-Leu-Val-Pro-Arg(Z_2_)-AOMK. The Cbz groups were then removed via hydrogenolysis (H_2_, Pd/C, DMF), and the *N*-terminal Boc protecting group was removed in TFA/DCM (1:1, v/v; with 3% TIPS for 30 min), followed by HPLC purification. Next, 1 eq of Cy5-NHS was dissolved in DMF, and 5 eq of DIPEA was added. The solution was immediately mixed with crude peptide-AOMK (1 eq). The reaction was carried out at room temperature for one hour. Reaction progress was monitored by HPLC (220 nm for H_2_N-Phe(guan)-Leu-Val-Pro-Arg-AOMK or 646 nm for Cy5-NHS). The final product was purified on HPLC to obtain Cy5-Phe(guan)-Leu-Val-Pro-Arg-AOMK (PKG105).

### MALT1 labeling with PKG105

MALT1^FL^, MALT1^CD^ or MALT1^C/A^ at 100 nM were incubated with PKG105 at three different concentrations: 500 nM, 250 nM and 125 nM for 30 minutes at 37 °C. As a control, MALT1^FL^ without the probe was incubated in the assay buffer. Samples were denatured in 3 × SDS-loading buffer for 5 minutes at 95 °C. SDS-PAGE was performed under reducing conditions for 37 minutes at 165 V. The fluorescence signal in the gel was measured using a Li-Cor (Odyssey®) instrument at a wavelength of 700 nm.

### Labeling of active MALT1 in RAW 264.7 cells

Twenty-five microliters of RAW 264.7 cell lysates (from 5 * 10^6^ cells/mL) were incubated with PKG105 at concentrations ranging from 8 to 500 nM. As controls, cells that were not incubated with probe or cells that were pretreated with 1 µL of 10 mM mepazine were treated with 500 nM PKG105. The lysates were incubated with the probe for 30 minutes at 37 °C, followed by denaturation in 3 × SDS-loading buffer for 5 minutes at 95 °C. SDS-PAGE was performed under reducing conditions for 37 minutes at 165 V. The fluorescence signal in the gel was measured using a Li-Cor (Odyssey^®^) at a wavelength of 700 nm.

### Synthesis of Internally Quenched Libraries

All internally quenched peptides were synthesized by solid-phase peptide synthesis on Fmoc-Rink amide AM resin (0.74 mmol). First, 2 g (1.48 mmol) Rink amide resin in a peptide synthesis vessel was activated for 30 minutes in DCM and washed 3 times with DFM. Next, the *N*-terminal Fmoc protecting group was removed from the resin with 20% piperidine solution in DMF for 5, 5 and 20 minutes and washed 6 times with DMF. The first amino acid, Fmoc-Gly-OH (2.5 eq, 1.85 mmol), was coupled to the resin using 2.5 eq (1.85 mmol) of DICI and 2.5 eq (1.85 mmol) of HOBt. The amino acid was incubated with coupling reagents in DMF for 3 minutes and poured onto the resin. The reaction was carried out for 3 hours. The resin was then washed 3 times with DMF to obtain Fmoc-Gly-Rink amide resin. Next, the Fmoc protecting group was removed with piperidine in DMF, and Fmoc-Lys(Dnp)-OH (2.5 eq, 1.85 mmol) was coupled to the resin. The Fmoc group was removed with piperidine in DMF. The product (H_2_N-Lys(Dnp)-Gly-resin) was washed 6 times with DMF, 3 times with DCM, and 3 times with MeOH, dried over P_2_O_5_ overnight, divided into 100-mg (0.074 mmol) portions and placed in the wells of a FlexChem multiwell synthesis block.

Synthesis proceeded in the multiwell vessel using the Fmoc strategy described above. To synthesize the P1’ library, 5 eq (0.37 mmol) of isokinetic mixture consisting of natural amino acids (without Met and Cys and with Nle) was activated with 5 eq (0.37 mmol) of DICI and 5 eq (0.37 mmol) of HOBt and poured onto the resin. The coupling reaction was conducted for 4 hours. The X-K(Dnp)-G-Rink amide resin was obtained, where X is an equimolar mixture of natural amino acids at the P2’ position. After removal of the Fmoc group, each of the compounds was treated in separate wells with 2.5 eq (0.185 mmol) of different natural amino acids (replacing Cys with Nle) activated with 2.5 eq (0.185 mmol) of DICI and 2.5 eq (0.185 mmol) of HOBt. The reaction was carried out for 3 hours, and peptide chain elongation proceeded until H_2_N-A-L-V-S-R-P1’-X-K(Dnp)-G-resin was obtained. Finally, Fmoc-ACC-OH (7-Fmoc-coumarin-4-acetic acid) was coupled to the *N*-terminal residue using 3 eq (0.222 mmol) of the fluorophore, 3 eq (0.222 mmol) of DICI and 3 eq (0.222 mmol) of HOBt. Coupling of the ACC molecule was carried out for 24 hours and repeated the next day for an additional 24 hours, followed by removal of the Fmoc protecting group. The resin was washed 6 times with DMF, 3 times with DCM and 2 times with MeOH and dried over P_2_O_5_ overnight. Peptides were cleaved from the resin using a TFA/H_2_O/TIPS mixture (95:2.5:2.5, v/v/v), and purified by HPLC semi-preparative chromatography with a Discovery BIO wide pore C8 10 µm (250 × 21.2 mm) column and a UV-vis detector. Peptides were dissolved in biochemical-grade dry DMSO to a final concentration of 10 mM and stored at −20 °C until use.

### Screening of internally quenched libraries

Individual components of the library were assayed with MALT1 at concentration of 2  (individual substrates were at a concentration of approximately 105 nM). MALT1^FL^ (80 nM) and MALT1^CD^ (160 nM) were preincubated at 37 °C in assay buffer for 20 minutes and added to the library (previously pipetted on Costar plates). Hydrolysis rates were calculated by measuring the release of the fluorophore over time (excitation at 355 nm and emission at 460 nm). The highest rate obtained for each sub-library was set as 100%, and the other rates were adjusted accordingly.

## Electronic supplementary material

Supplementary Dataset 1

## Data Availability

The authors declare that all relevant data supporting the findings of this study are available within the paper (and its Supplementary information file). Any raw data can be obtained from the corresponding authors on reasonable request.
