# Supplementary material for: Determination of extended substrate specificity of the MALT1 as a strategy for the design of potent substrates and activity-based probes
Source: Sci Rep. 2018 Oct 30;8:15998. doi: 10.1038/s41598-018-34476-7 (PMC6207715; doi:10.1038/s41598-018-34476-7)

Determination of extended substrate specificity of the MALT1 as a strategy for the design of potent substrates and activity-based probes.

Paulina Kasperkiewicz[1,*], Sonia Kołt[1], Tomasz Janiszewski[1], Katarzyna Groborz[1], Marcin Poręba[1,2], Scott J. Snipas[2], Guy S. Salvesen[2,*], Marcin Drąg[1,*]

[1] Department of Bioorganic Chemistry, Faculty of Chemistry, Wroclaw University of Science and Technology, Wyb. Wyspianskiego 27, 50-370 Wroclaw, Poland; [2] NCI-designated Cancer Center, Sanford Burnham Prebys Medical Discovery Institute, La Jolla, CA 92037, USA

*corresponding authors

SUPPLEMENTAL INFORMATION

Reagents

All chemicals were purchased from commercial suppliers and used without further purification. All Fmoc-labeled amino acids (purity > 99%) used for synthesis were purchased from Iris Biotech GmbH (Germany), Combi-Blocks (USA), QMBIO (China), CreoSalus (USA), Bachem (Germany), ApexBio (USA). Fmoc-Rink amide AM polystyrene resin (loading, 0.74 mmol/g) was obtained from Iris Biotech GmbH; HOBt (*N*-hydroxybenzotriazole, purity > 98%) was purchased from CreoSalus (USA); DCM (dichloromethane, analytical grade purity) was purchased from POCh (Poland); MeOH (methanol, analytical grade purity) was purchased from POCh; DICI (diisopropylcarbodiimide, peptide grade), HBTU (*O*-benzotriazole-*N*,*N*,*N*',*N*'-tetramethyl-uronium-hexafluoro-phosphate, peptide grade), HATU (2-(1-H-7-azabenzotriazol-1-yl)-1,1,3,3-tetramethyl uranium hexafluorophosphatemethanaminium, peptide grade), and DIPEA (*N*,*N*-diisopropylethylamine, peptide grade), were purchased from Iris Biotech GmbH (Germany); Pip (piperidine; purity, 99%) and collidine (2,4,6-trimethylpyridine, peptide grade) were purchased from Sigma-Aldrich (Poland); DMF (*N*,*N*'-dimethylformamide, peptide grade) and ACN (acetonitrile, HPLC-gradient grade) were purchased from Avantor; Et_2O (diethyl ether, analytical grade purity) and AcOH (acetic acid; purity, 99%) were purchased from POCh; TFA (trifluoroacetic acid; purity, 99%) was purchased from Iris Biotech GmbH; TIPS (triisopropylsilane; purity, 99%) was purchased from Sigma-Aldrich (Poland). All individual substrates were purified by HPLC (Waters M600 solvent delivery module, Waters M2489 detector system, Waters Spherisorb S10ODS2 column; Waters sp. z.o.o., Poland). The solvents used were as follows: Phase A (water/0.1% TFA), Phase B (acetonitrile/0.1% TFA). Substrate purity was tested by analytical HPLC (Waters Spherisorb S5ODS2 column). Finally, the molecular weights were determined by mass spectrometry (Waters LCT Premier XE high-resolution mass spectrometer, electrospray ionization (ESI), and time-of-flight (TOF) detector).

Combinatorial and defined substrate library screening

Library screening was performed with the conditions described above. The total volume of the reaction was 100 µL per well. The substrate concentration used for the assay was

1 μM per well. The measurement was carried out for 20-30 minutes. The values obtained for each substrate were compared to the values obtained for the best substrate (highest production of relative fluorescence units per second), and the results were plotted as a graph.

Individual substrate synthesis

To determine the best peptide sequences for the enzyme, we synthesized ACC-labeled fluorogenic substrates by a previously described method[16]. The Fmoc-ACC-OH fluorophore was synthesized as described by Maly et al.[23]. First, 2 g of Rink amide resin (1.48 mmol) was added to a glass reaction vessel, which was followed by the addition of 6 mL of DCM. The resin was gently stirred once every 10 minutes for one hour and then washed three times with DMF. The *N*-terminal Fmoc protecting group was removed by using 20% piperidine in DMF (three cycles: 5 minutes, 5 minutes and 25 minutes), and the resin was washed six times with DMF. Next, 3 eq of Fmoc-ACC-OH preactivated with 3 eq of HOBt and 3 eq of DICI was added to the resin, and the ACC coupling reaction was carried out for 24 hours. Then, the reaction was repeated by using 1.5 eq of the above reagents to improve the yield of fluorophore coupling. Then, the Fmoc protecting groups were removed as previously described. To attach the P1 amino acid, 3 eq of Fmoc-Arg(Pbf)-OH preactivated with 3 eq of HATU and 3 eq of collidine was added to the resin and gently agitated for 24 hours at room temperature. The reaction was repeated by using the same amounts of the above reagents. Next, the resin was washed three times with DMF, and the Fmoc protecting group was removed by using 20% piperidine in DMF. The resin was washed six times with DMF, three times with DCM, and three times with MeOH and dried over P_2O_5. In the next stage, 14 equal portions of H_2N-Arg-Rink amide resin (100 mg, 0.074 mmol) were added to a solid-phase peptide synthesizer and stirred gently once every 10 minutes in DCM for 1 hour and then filtered and washed with DMF. Next, 2.5 eq of Fmoc-P2-OH was preactivated with 3 eq of HOBt and DICI in DMF, and the mixture was poured onto the resin. The reaction was carried out for three hours in room temperature, and then, the resin was washed with DMF, and the Fmoc protecting group was removed by using 20% piperidine in DMF. The P3, P4 and P5 positions were coupled in the same manner as the P2 position. A ninhydrin test

was carried out after each coupling and Fmoc deprotection step. The *N* terminus was protected with an acetyl group using 5 eq AcOH, 5 eq HBTU, and 5 eq DIPEA in DMF. After solvent removal, the resin was washed six times with DMF in order to remove excess reagents, three times with DCM, and three times with MeOH and then dried over P_2O_5 and cleaved from the resin with a TFA/TIPS/H_2O mixture (%, v/v/v, 95:2.5:2.5). The crude product was purified by HPLC and lyophilized. The purity of each substrate was confirmed by analytical HPLC, and each substrate was analyzed using HRMS. Substrates were dissolved in peptide-grade DMSO to a concentration of 20 mM and stored at -80°C until use.

Calculation of k_{cat}, K_m and k_{cat}/K_m values

Kinetic parameters were calculated under the same conditions as the library with slight modification. The total reaction volume in each well was 100 µL. The substrate and enzyme concentrations used ranged from 3.90 µM to 150 µM and 40 µM, respectively. The increase in relative fluorescence units was monitored for 20-30 minutes, and only the linear parts of the curves were used to determine the rate of the reaction. The k_{cat} and K_m values were calculated using GraphPad Prism software. All measurements were repeated three times using the appropriate concentration of the enzyme, and the data presented are averages of these repetitions.

Synthesis of the P2' library

Synthesis of the P2' library was conducted using the same method as that used for the P1' library, with the exception that the P2' position was occupied with a set of natural amino acids (replacing Cys with Nle), and the P1' position contained an equimolar mixture of natural amino acids (replacing Met and Cys with Nle).

Synthesis of internally quenched substrates

All the internally quenched substrates were synthesized in the same manner as the internally quenched libraries with slight modification. For the defined substrates, the P1' position was fixed with Gly, and the P2' positions contained natural amino acids and a few unnatural amino acids.

Investigation of the P2' pocket

Screening of defined substrates for investigation of the P2' pocket was conducted using the same method as that used for screening the libraries, with the exception of the final concentrations of the enzymes (160 nM catalytic domain and 120 nM MALT1).

Cleavage site determination

To determinate the MALT1 cleavage site, 2.5 μL of the MALT1 substrate SKM3 was added to 500 μL of assay buffer (pH 7.5) in a plastic tube and stirred well to obtain a 50-μM solution of the SKM3 peptide. Then, 8 μL of full-length MALT1 was added to obtain a protease concentration of 160 nM. The mixture was incubated at 37°C. The progress of the hydrolysis reaction was monitored after 2 and 5 hours by analytical HPLC with a Discovery BIO wide pore C8 10-μm (250 × 4.6 mm) column. To perform the analysis, 100 μL of the sample was diluted fourfold by adding 300 μL of ACN:H_2O (3:1, v/v). The injection volume was 30 μL, and a linear gradient from 0 to 100% B (where A is 0.1% TFA in H_2O, and B is 0.1% TFA in ACN) at a flow rate of 1.5 mL/minute was applied for 20 minutes. After 2 hours of incubation, the substrate was found to be hydrolyzed poorly, with only very small peaks observed for the two peptide fragments. Elongation of the reaction time to 5 hours allowed us to observe the amount of intact peptide decreased in contrast to increased amount of the two hydrolyzed peptides. The first peak corresponds to the peptide with the fluorophore at the C-terminal end, and the second peak corresponds to the peptide with quencher at the N terminus.

Because of the low substrate concentration, molecular weight analysis could be performed on only the reaction mixture, which was analyzed by high-resolution mass spectrometry on a Waters LCT Premier XE high-resolution mass spectrometer with ESI and TOF. Individually collected peptide fractions did not produce signals strong enough to be identify the molecular masses of the peptides. The mass spectrum confirmed the cleavage site, which was present between the P1 and P1' amino acid residues. We did not detect any peaks that corresponded to peptide fragments obtained after the hydrolysis of any other bond.

Compounds analysis

Ac-Gly-Leu-Val-Ser-Arg-ACC HRMS (m/z) [MH+] calcd. for $C_{35}H_{52}N_{10}O_{10}$, 773.39; found, 773.39

Ac-Pro-Leu-Val-Ser-Arg-ACC HRMS (m/z) [MH+] calcd. for $C_{38}H_{56}N_{10}O_{10}$, 813.42; found, 813.39

Ac-Val-Leu-Val-Ser-Arg-ACC HRMS (m/z) [MH+] calcd. for $C_{38}H_{58}N_{10}O_{10}$, 815.44; found, 815.38

Ac-Leu-Leu-Val-Ser-Arg-ACC HRMS (m/z) [MH+] calcd. for $C_{39}H_{60}N_{10}O_{10}$, 829.45; found, 829.38

Ac-Ile-Leu-Val-Ser-Arg-ACC HRMS (m/z) [MH+] calcd. for $C_{39}H_{60}N_{10}O_{10}$, 829.45; found, 829.38

Ac-Ala-Leu-Val-Ser-Arg-ACC HRMS (m/z) [MH+] calcd. for $C_{36}H_{54}N_{10}O_{10}$, 787.41; found, 787.35

Ac-Phe-Leu-Val-Ser-Arg-ACC HRMS (m/z) [MH+] calcd. for $C_{42}H_{58}N_{10}O_{10}$, 863.44; found, 863.45

Ac-Tyr-Leu-Val-Ser-Arg-ACC HRMS (m/z) [MH+] calcd. for $C_{42}H_{58}N_{10}O_{11}$, 879.43; found, 879.35

Ac-His-Leu-Val-Ser-Arg-ACC HRMS (m/z) [MH+] calcd. for $C_{39}H_{56}N_{12}O_{10}$, 853.43; found, 863.43

Ac-Trp-Leu-Val-Ser-Arg-ACC HRMS (m/z) [MH+] calcd. for $C_{44}H_{59}N_{11}O_{10}$, 902.45; found, 902.45

Ac-Met-Leu-Val-Ser-Arg-ACC HRMS (m/z) [MH+] calcd. for $C_{44}H_{59}N_{11}O_{10}$, 847.41; found, 847.41

Ac-Ser-Leu-Val-Ser-Arg-ACC HRMS (m/z) [MH+] calcd. for $C_{44}H_{59}N_{11}O_{10}$, 803.41; found, 803.40

Ac-Thr-Leu-Val-Ser-Arg-ACC HRMS (m/z) [MH$^+$] calcd. for $C_{37}H_{56}N_{10}O_{11}$, 817.42; found, 817.42

Ac-Arg-Leu-Val-Ser-Arg-ACC HRMS (m/z) [MH$^+$] calcd. for $C_{39}H_{61}N_{13}O_{10}$, 872.47; found, 872.47

Ac-Lys-Leu-Val-Ser-Arg-ACC HRMS (m/z) [MH$^+$] calcd. for $C_{39}H_{61}N_{11}O_{10}$, 844.46; found, 844.46

Ac-Asp-Leu-Val-Ser-Arg-ACC HRMS (m/z) [MH$^+$] calcd. for $C_{37}H_{54}N_{10}O_{12}$, 831.40; found, 831.40

Ac-Glu-Leu-Val-Ser-Arg-ACC HRMS (m/z) [MH$^+$] calcd. for $C_{38}H_{56}N_{10}O_{12}$, 845.41; found, 845.41

Ac-Asn-Leu-Val-Ser-Arg-ACC HRMS (m/z) [MH$^+$] calcd. for $C_{37}H_{55}N_{11}O_{11}$, 830.41; found, 830.41

Ac-Gln-Leu-Val-Ser-Arg-ACC HRMS (m/z) [MH$^+$] calcd. for $C_{38}H_{57}N_{11}O_{11}$, 844.43; found, 844.43

Ac-NLeu-Leu-Val-Ser-Arg-ACC HRMS (m/z) [MH$^+$] calcd. for $C_{39}H_{60}N_{10}O_{10}$, 829.45; found, 829.45

Ac-D-Ala-Leu-Val-Ser-Arg-ACC HRMS (m/z) [MH$^+$] calcd. for $C_{36}H_{54}N_{10}O_{10}$, 787.41; found, 787.41

Ac-Abu-Leu-Val-Ser-Arg-ACC HRMS (m/z) [MH$^+$] calcd. for $C_{36}H_{55}N_{11}O_{11}$, 801.41; found, 801.42

Ac-B-Ala-Leu-Val-Ser-Arg-ACC HRMS (m/z) [MH$^+$] calcd. for $C_{36}H_{54}N_{10}O_{10}$, 787.41; found, 787.41

Ac-D-Val-Leu-Val-Ser-Arg-ACC HRMS (m/z) [MH$^+$] calcd. for $C_{38}H_{58}N_{10}O_{10}$, 815.44; found, 815.44

Ac-D-Leu-Leu-Val-Ser-Arg-ACC HRMS (m/z) [MH^+] calcd. for $C_{39}H_{60}N_{10}O_{10}$, 829.45; found, 829.45

Ac-Phe(guan)-Leu-Val-Ser-Arg-ACC HRMS (m/z) [MH^+] calcd. for $C_{43}H_{61}N_{13}O_{10}$, 920.47; found, 920.47

Ac-hTyr(Me)-Leu-Val-Ser-Arg-ACC HRMS (m/z) [MH^+] calcd. for $C_{44}H_{62}N_{10}O_{11}$, 907.46; found, 907.46

Ac-Cha-Leu-Val-Ser-Arg-ACC HRMS (m/z) [MH^+] calcd. for $C_{42}H_{64}N_{10}O_{10}$, 869.48; found, 869.48

Ac-His(Bzl)-Leu-Val-Ser-Arg-ACC HRMS (m/z) [MH^+] calcd. for $C_{46}H_{62}N_{12}O_{10}$, 943.47; found, 943.47

Ac-hArg-Leu-Val-Ser-Arg-ACC HRMS (m/z) [MH^+] calcd. for $C_{40}H_{63}N_{13}O_{10}$, 886.49; found, 886.49

Ac-Agp-Leu-Val-Ser-Arg-ACC HRMS (m/z) [MH^+] calcd. for $C_{37}H_{57}N_{13}O_{10}$, 844.44; found, 844.44

Ac-Lys(2ClZ)-Leu-Val-Ser-Arg-ACC HRMS (m/z) [MH^+] calcd. for $C_{47}H_{66}ClN_{11}O_{12}$, 1012.46; found, 1012.46

Ac-Lys(Ac)-Leu-Val-Ser-Arg-ACC HRMS (m/z) [MH^+] calcd. for $C_{41}H_{63}N_{11}O_{11}$, 886.47; found, 886.47

Ac-Lys(TFA)-Leu-Val-Ser-Arg-ACC HRMS (m/z) [MH^+] calcd. for $C_{41}H_{60}F_3N_{11}O_{11}$, 940.45; found, 940.45

Ac-Orn-Leu-Val-Ser-Arg-ACC HRMS (m/z) [MH^+] calcd. for $C_{38}H_{59}N_{11}O_{10}$, 830.45; found, 830.45

Ac-hCit-Leu-Val-Ser-Arg-ACC HRMS (m/z) [MH^+] calcd. for $C_{40}H_{62}N_{12}O_{11}$, 887.47; found, 887.47

Ac-Cit-Leu-Val-Ser-Arg-ACC HRMS (m/z) [MH^+] calcd. for $C_{39}H_{60}N_{12}O_{11}$, 873.45; found, 873.45

Ac-Dab(Z)-Leu-Val-Ser-Arg-ACC HRMS (m/z) [MH^+] calcd. for $C_{45}H_{63}N_{11}O_{12}$, 950.47; found, 950.47

Ac-Dab-Leu-Val-Ser-Arg-ACC HRMS (m/z) [MH^+] calcd. for $C_{37}H_{57}N_{11}O_{10}$, 816.43; found, 816.43

Ac-Dap-Leu-Val-Ser-Arg-ACC HRMS (m/z) [MH^+] calcd. for $C_{36}H_{55}N_{11}O_{10}$, 802.42; found, 802.42

ACC-Ahx-Ala-Leu-Val-Ser-Arg-Gly-Ala-Lys(Dnp)-Gly HRMS (m/z): [MH^+] calcd for $C_{59}H_{88}N_{18}O_{18}$, 1337,6603; found, 1337,6615

ACC-Ahx-Ala-Leu-Val-Ser-Arg-Gly-Arg-Lys(Dnp)-Gly HRMS (m/z): [MH^+] calcd for $C_{62}H_{95}N_{21}O_{18}$, 1422,7242; found, 711,8661

ACC-Ahx-Ala-Leu-Val-Ser-Arg-Gly-Asn-Lys(Dnp)-Gly HRMS (m/z): [MH^+] calcd for $C_{60}H_{89}N_{19}O_{19}$, 1380,6660; found, 1380,6683

ACC-Ahx-Ala-Leu-Val-Ser-Arg-Gly-Asp-Lys(Dnp)-Gly HRMS (m/z): [MH^+] calcd for $C_{60}H_{88}N_{18}O_{20}$, 1381,6500; found, 691,3296

ACC-Ahx-Ala-Leu-Val-Ser-Arg-Gly-Glu-Lys(Dnp)-Gly HRMS (m/z): [MH^+] calcd for $C_{61}H_{90}N_{18}O_{20}$, 1395,6656; found, 698,3362

ACC-Ahx-Ala-Leu-Val-Ser-Arg-Gly-Gln-Lys(Dnp)-Gly HRMS (m/z): [MH^+] calcd for $C_{61}H_{91}N_{19}O_{19}$, 1394,6816; found, 697,8435

ACC-Ahx-Ala-Leu-Val-Ser-Arg-Gly-Gly-Lys(Dnp)-Gly HRMS (m/z): [MH^+] calcd for $C_{58}H_{86}N_{18}O_{18}$, 1323,6445; found, 662,3245

ACC-Ahx-Ala-Leu-Val-Ser-Arg-Gly-His-Lys(Dnp)-Gly HRMS (m/z): [MH^+] calcd for $C_{62}H_{90}N_{20}O_{18}$, 1403,6776; found, 702,3446

ACC-Ahx-Ala-Leu-Val-Ser-Arg-Gly-Ile-Lys(Dnp)-Gly HRMS (m/z): [MH^+] calcd for $C_{62}H_{94}N_{18}O_{18}$, 1379,7072; found, 690,3569

ACC-Ahx-Ala-Leu-Val-Ser-Arg-Gly-Leu-Lys(Dnp)-Gly HRMS (m/z): [MH^+] calcd for $C_{62}H_{94}N_{18}O_{18}$, 1379,7072; found, 690,3576

ACC-Ahx-Ala-Leu-Val-Ser-Arg-Gly-Lys-Lys(Dnp)-Gly HRMS (m/z): [MH^+] calcd for $C_{62}H_{95}N_{19}O_{18}$, 1394,7136; found, 697,8629

ACC-Ahx-Ala-Leu-Val-Ser-Arg-Gly-Nle-Lys(Dnp)-Gly HRMS (m/z): [MH^+] calcd for $C_{62}H_{94}N_{18}O_{18}$, 1379,7072,6603; found, 690,3591

ACC-Ahx-Ala-Leu-Val-Ser-Arg-Gly-Phe-Lys(Dnp)-Gly HRMS (m/z): [MH^+] calcd for $C_{65}H_{92}N_{18}O_{18}$, 1413,6871; found, 707,3502

ACC-Ahx-Ala-Leu-Val-Ser-Arg-Gly-Pro-Lys(Dnp)-Gly HRMS (m/z): [MH^+] calcd for $C_{61}H_{90}N_{18}O_{18}$, 1363,6759; found, 682,3411

ACC-Ahx-Ala-Leu-Val-Ser-Arg-Gly-Ser-Lys(Dnp)-Gly HRMS (m/z): [MH^+] calcd for $C_{59}H_{88}N_{18}O_{19}$, 1353,6507; found, 677,3304

ACC-Ahx-Ala-Leu-Val-Ser-Arg-Gly-Thr-Lys(Dnp)-Gly HRMS (m/z): [MH^+] calcd for $C_{60}H_{90}N_{18}O_{19}$, 1367,6708; found, 684,3381

ACC-Ahx-Ala-Leu-Val-Ser-Arg-Gly-Trp-Lys(Dnp)-Gly HRMS (m/z): [MH^+] calcd for $C_{67}H_{93}N_{19}O_{18}$, 1452,6980; found, 726,8544

ACC-Ahx-Ala-Leu-Val-Ser-Arg-Gly-Tyr-Lys(Dnp)-Gly HRMS (m/z): [MH^+] calcd for $C_{65}H_{92}N_{18}O_{19}$, 1429,6820; found, 715,3482

ACC-Ahx-Ala-Leu-Val-Ser-Arg-Gly-Val-Lys(Dnp)-Gly HRMS (m/z): [MH^+] calcd for $C_{61}H_{92}N_{18}O_{18}$, 1365,6871; found, 683,3505

ACC-Ahx-Ala-Leu-Val-Ser-Arg-Gly-Met-Lys(Dnp)-Gly HRMS (m/z): [MH^+] calcd for $C_{61}H_{92}N_{18}O_{18}S$, 1397,6591; found, 699,3345

ACC-Ahx-Ala-Leu-Val-Ser-Arg-Gly-hSer-Lys(Dnp)-Gly HRMS (m/z): [MH^+] calcd for $C_{60}H_{90}N_{18}O_{19}$, 1367,6663; found, 684,3383

ACC-Ahx-Ala-Leu-Val-Ser-Arg-Gly-Abu-Lys(Dnp)-Gly HRMS (m/z): [MH^+] calcd for $C_{60}H_{90}N_{18}O_{18}$, 1351,6714; found, 676,3420

ACC-Ahx-Ala-Leu-Val-Ser-Arg-Gly-β-Ala-Lys(Dnp)-Gly HRMS (m/z): [MH^+] calcd for $C_{59}H_{88}N_{18}O_{18}$, 1337,6558; found, 669,3337

ACC-Ahx-Ala-Leu-Val-Ser-Arg-Gly-Tle-Lys(Dnp)-Gly HRMS (m/z): [MH^+] calcd for $C_{62}H_{94}N_{18}O_{18}$, 1379,7027; found, 690,3563

ACC-Ahx-Ala-Leu-Val-Ser-Arg-Gly-Nva-Lys(Dnp)-Gly HRMS (m/z): [MH^+] calcd for $C_{61}H_{92}N_{18}O_{18}$, 1365,6871; found, 683,3499

ACC-Ahx-Ala-Leu-Val-Ser-Arg-Leu-Ala-Lys(Dnp)-Gly HRMS (m/z): [MH^+] calcd for $C_{63}H_{96}N_{18}O_{18}$, 1393,7184; found, 697,3640

Compounds structure, MS and HPLC analysis

Ac-Gly-Leu-Val-Ser-Arg-ACC

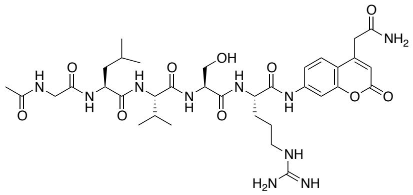

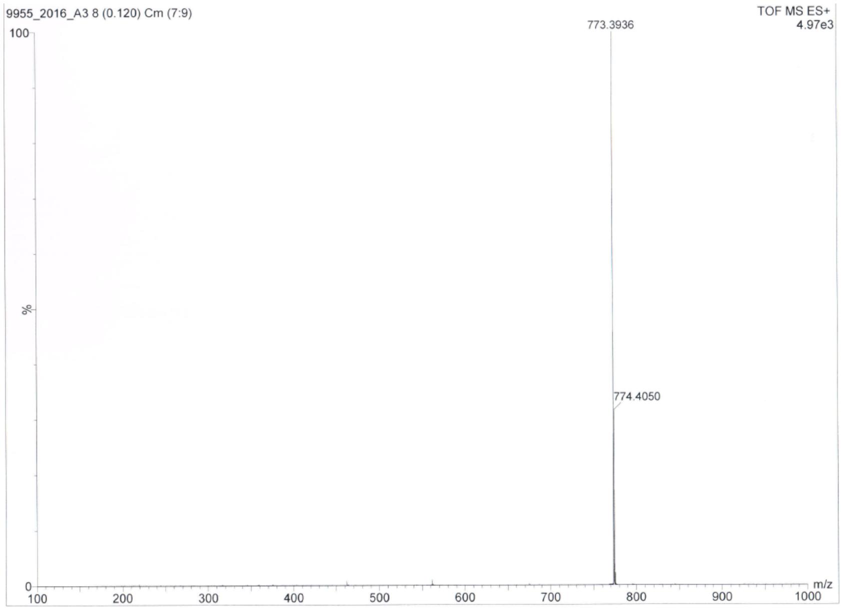

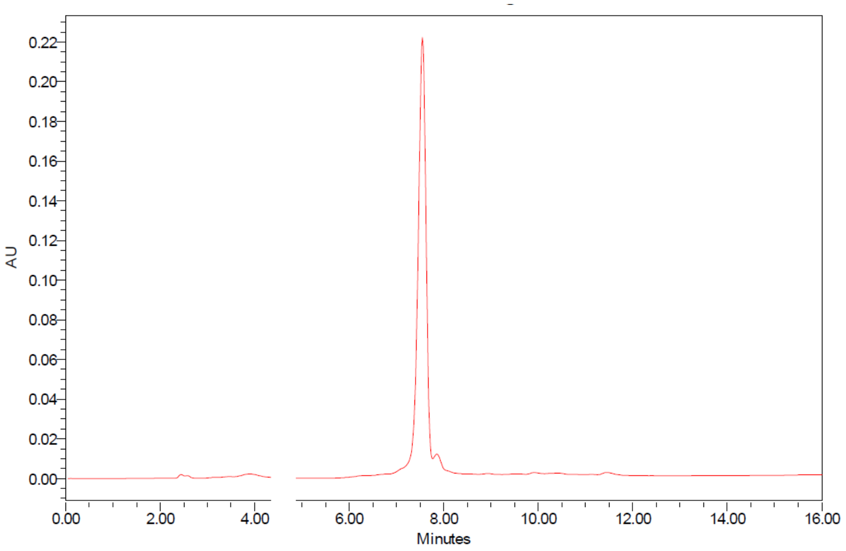

Ac-Pro-Leu-Val-Ser-Arg-ACC

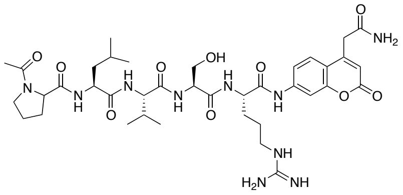

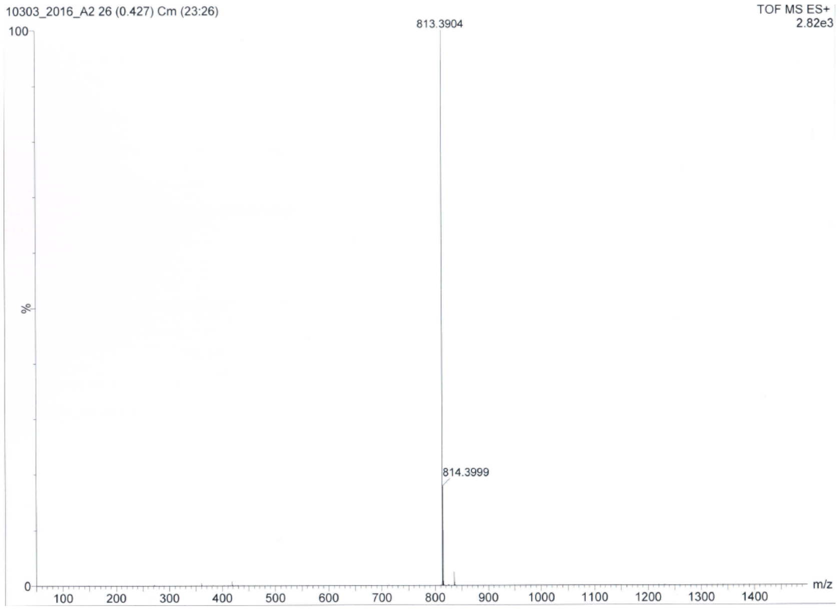

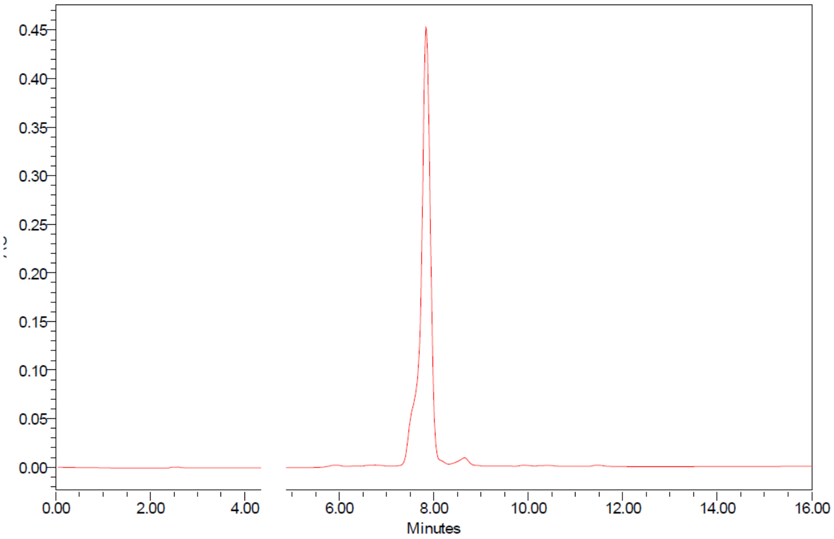

Ac-Val-Leu-Val-Ser-Arg-ACC

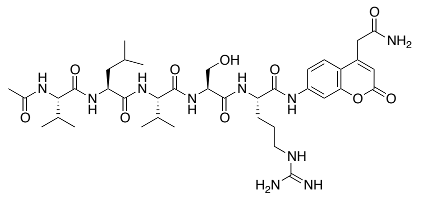

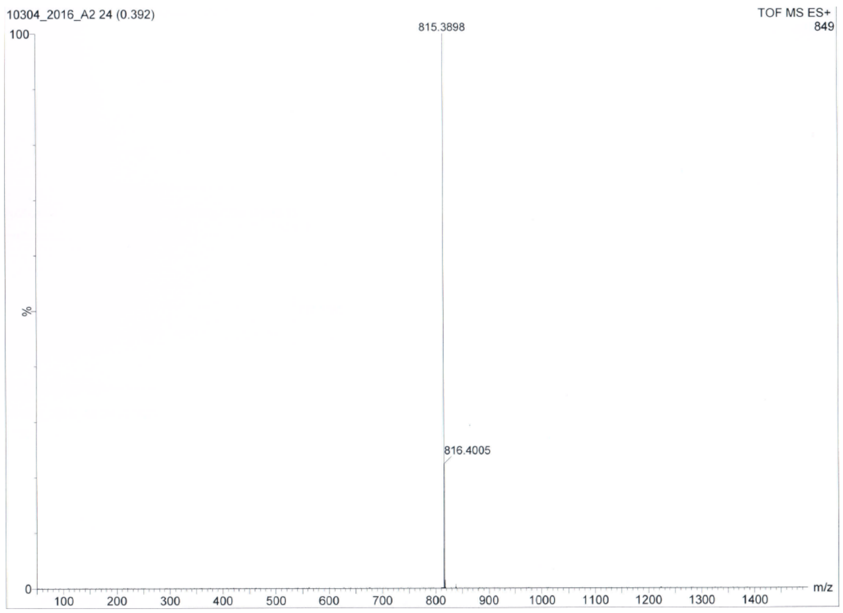

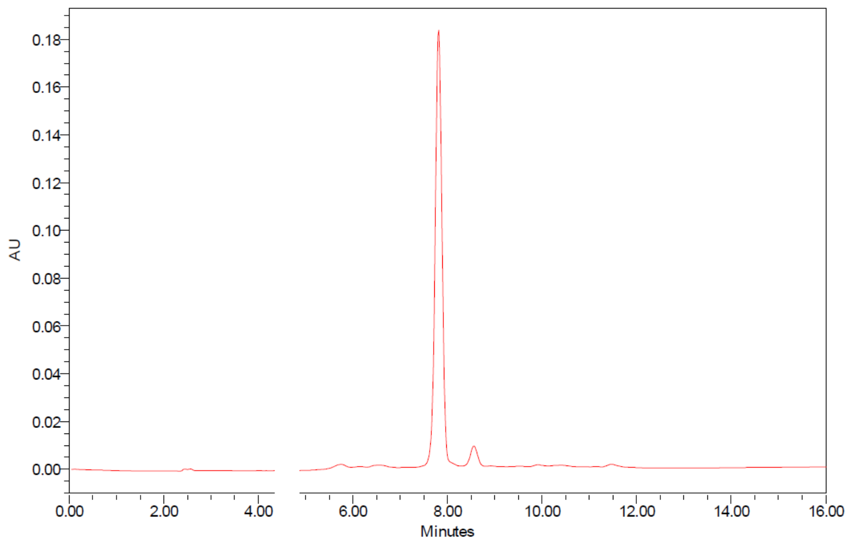

Ac-Leu-Leu-Val-Ser-Arg-ACC

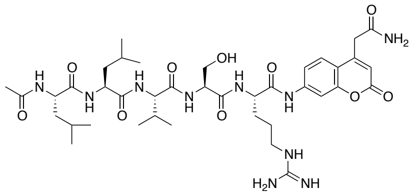

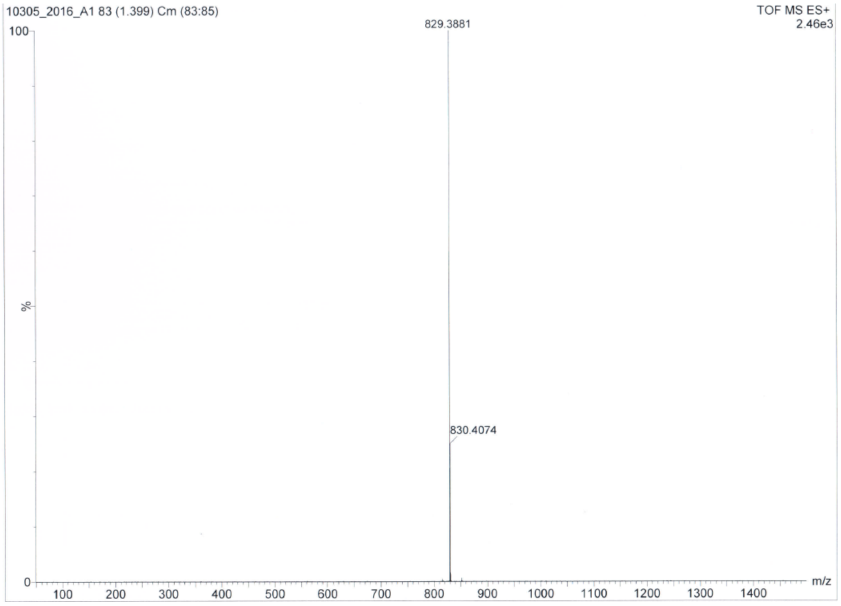

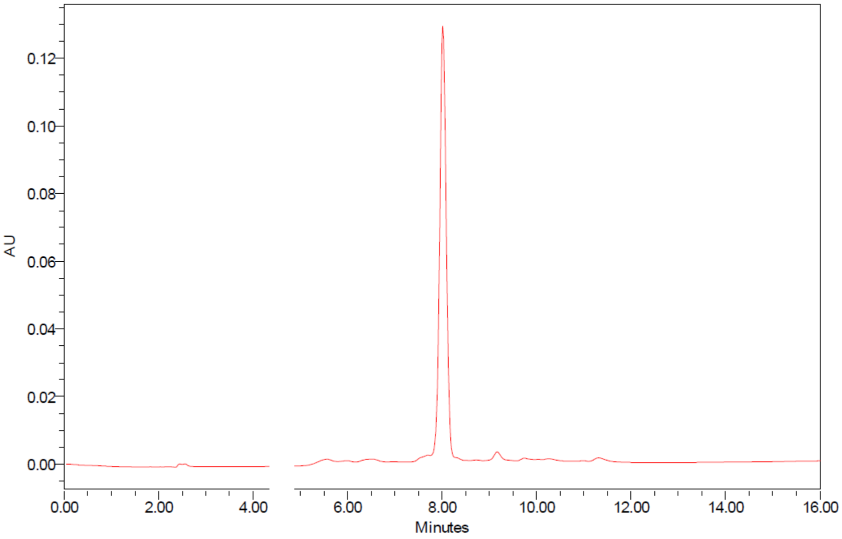

Ac-Ile-Leu-Val-Ser-Arg-ACC

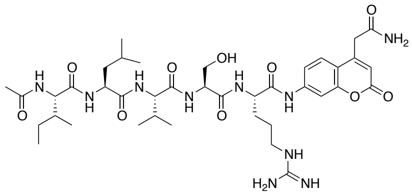

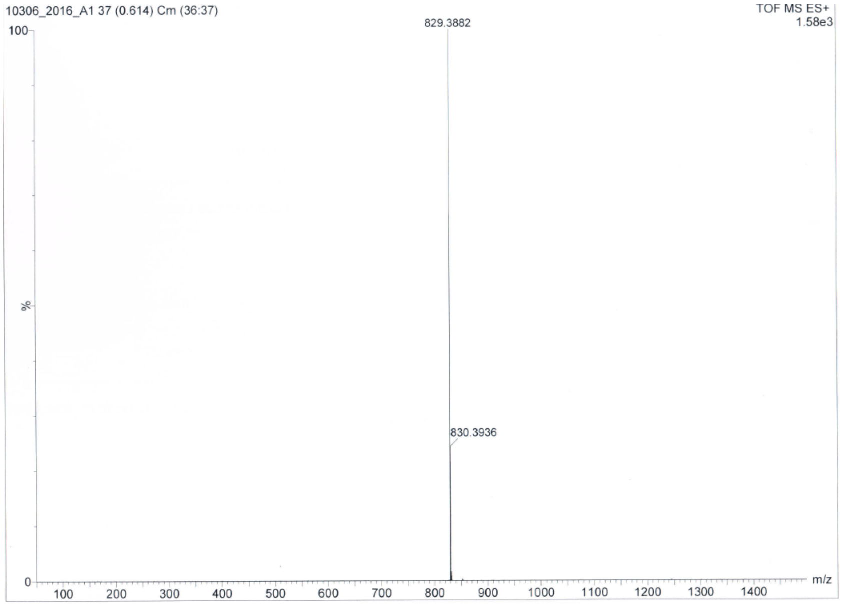

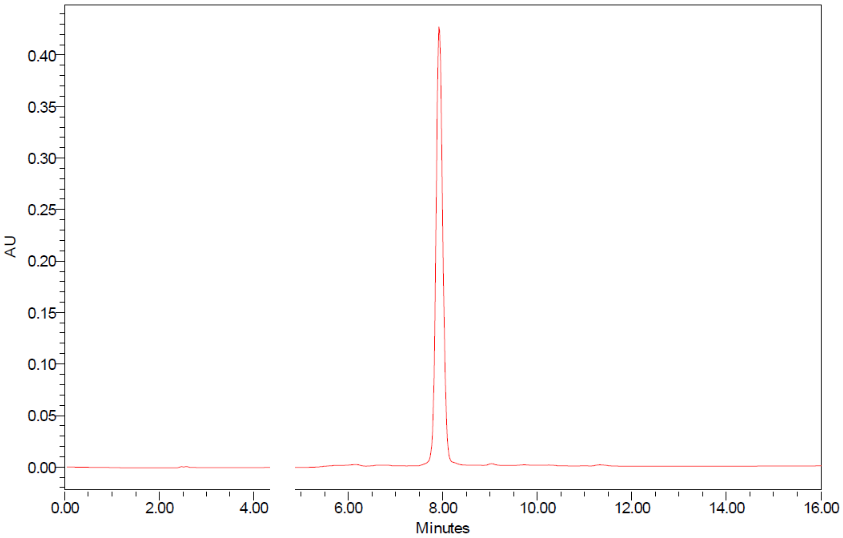

Ac-Ala-Leu-Val-Ser-Arg-ACC

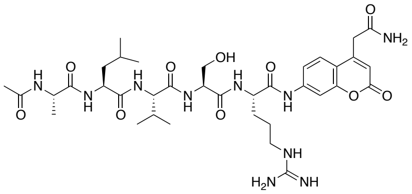

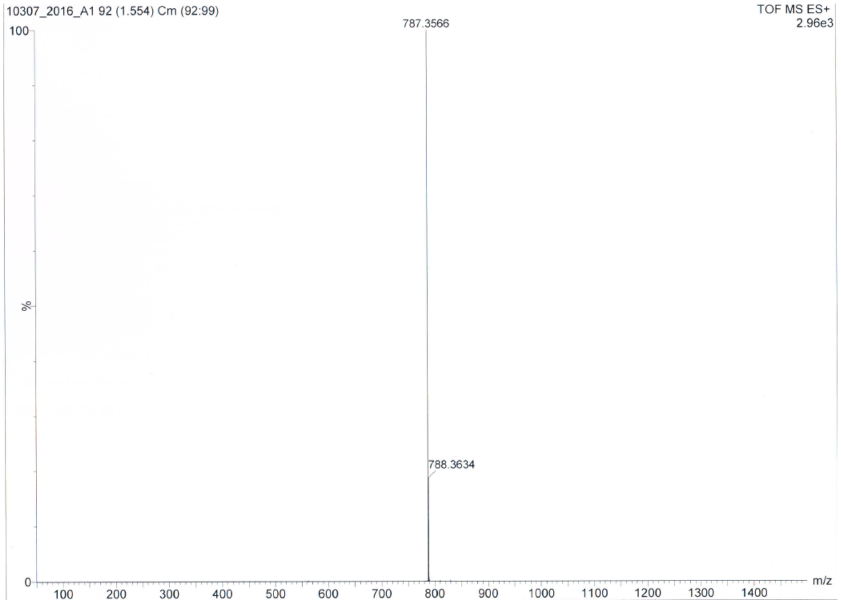

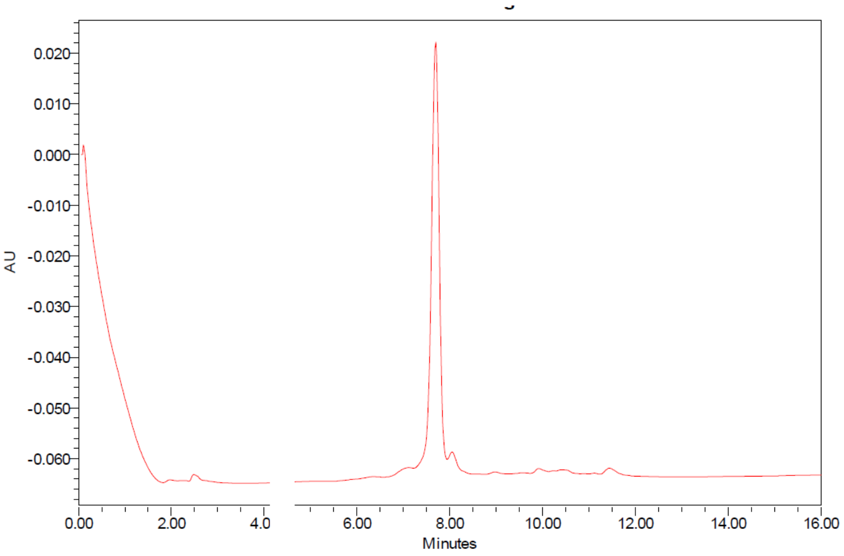

Ac-Phe-Leu-Val-Ser-Arg-ACC

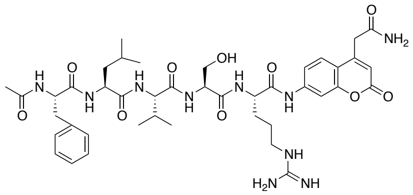

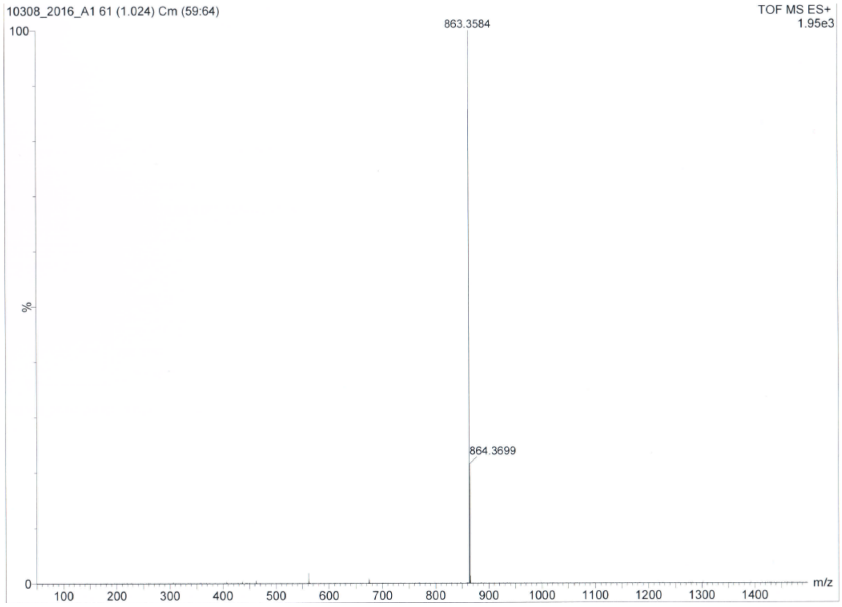

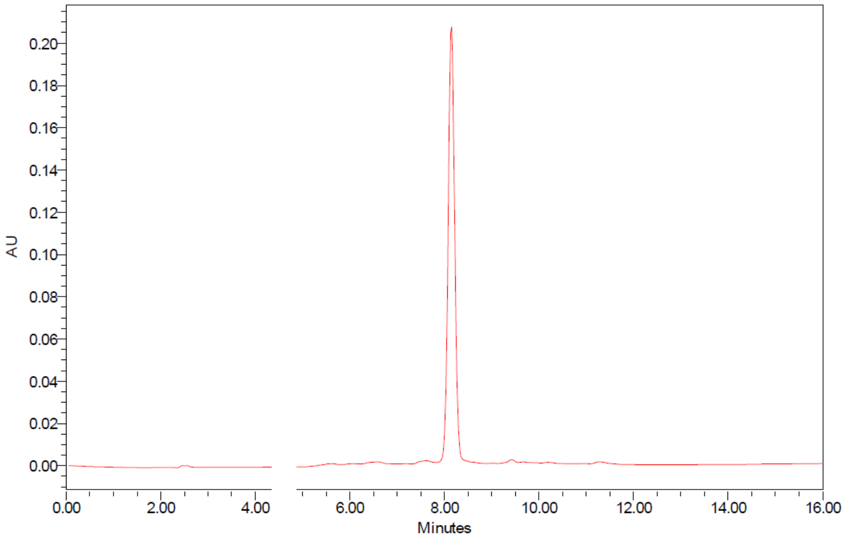

Ac-Tyr-Leu-Val-Ser-Arg-ACC

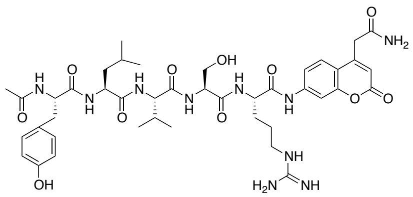

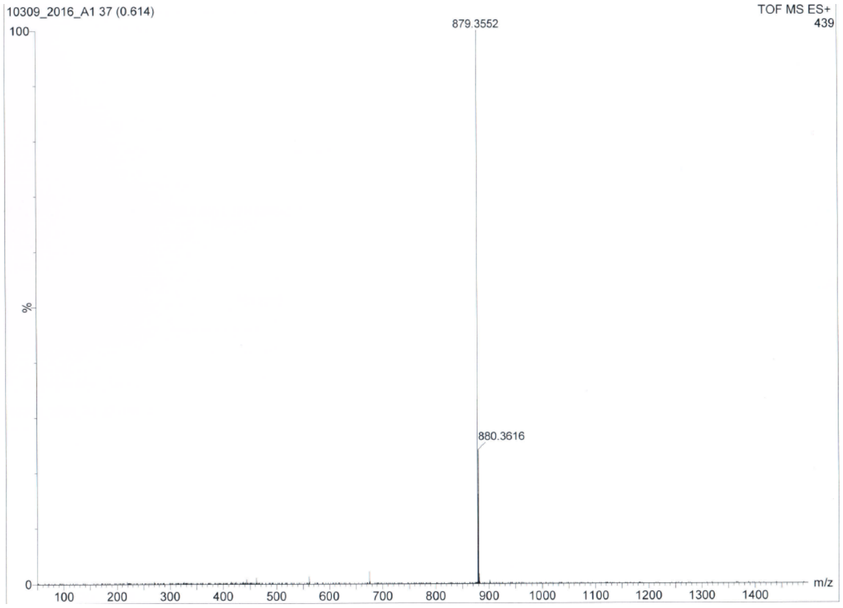

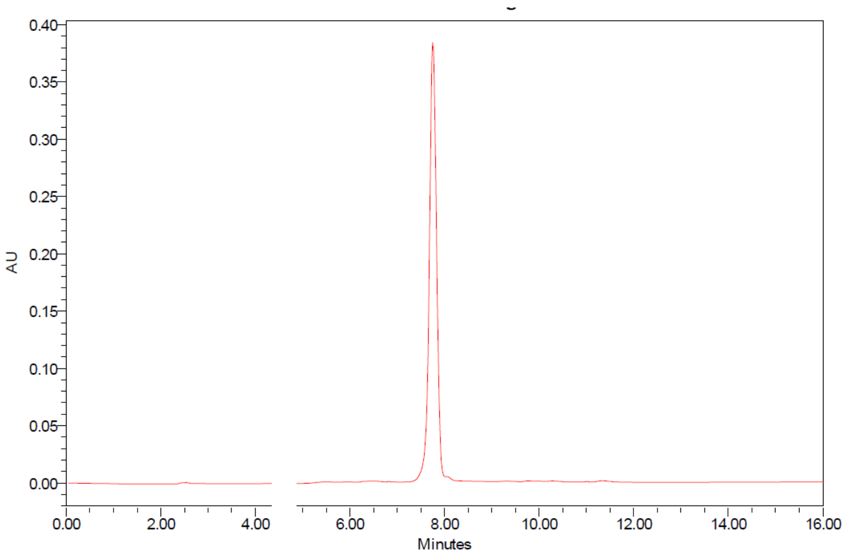

Ac-His-Leu-Val-Ser-Arg-ACC

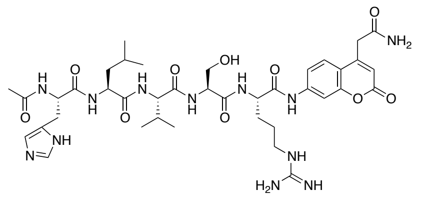

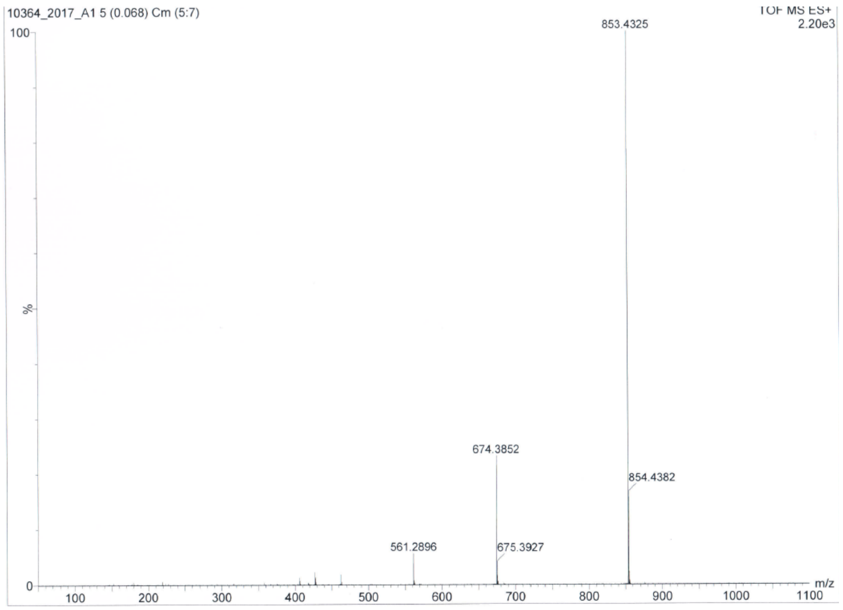

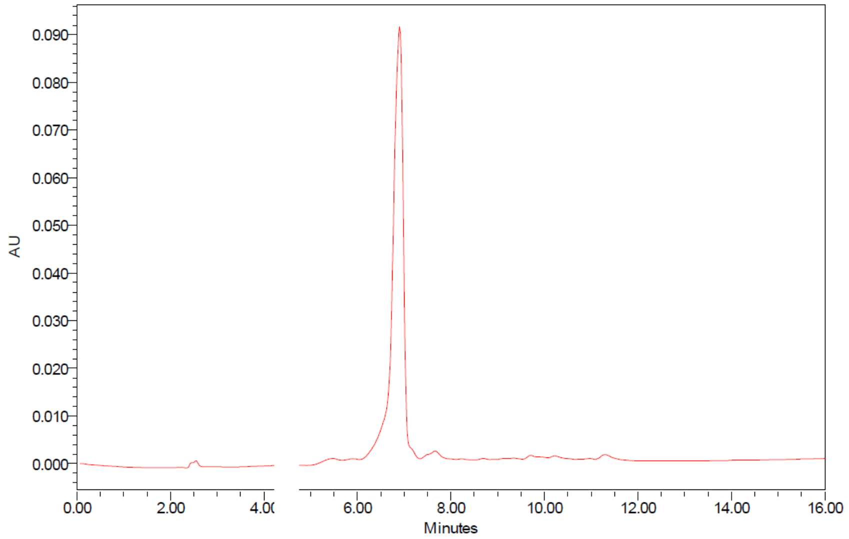

Ac-Trp-Leu-Val-Ser-Arg-ACC

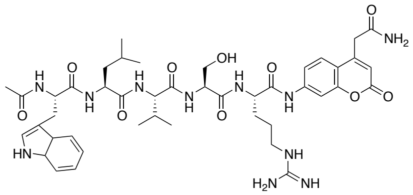

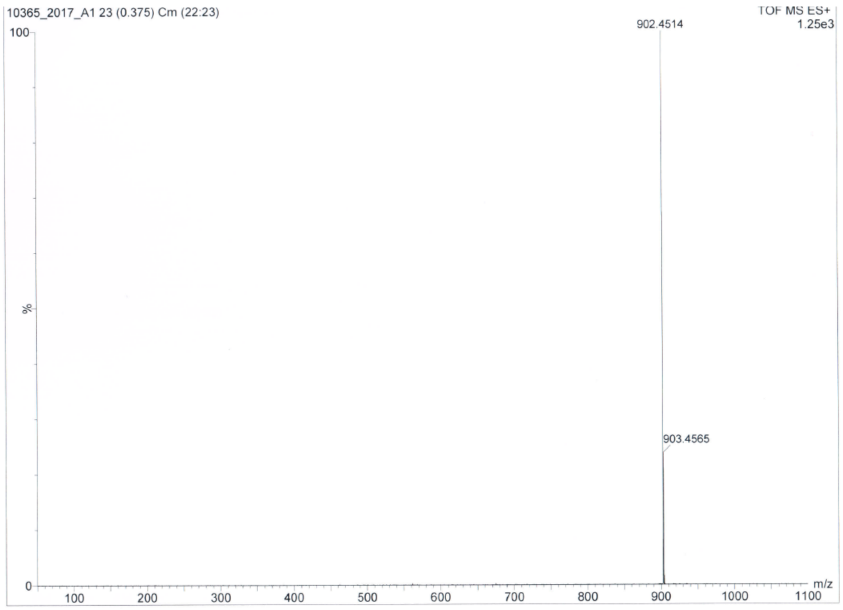

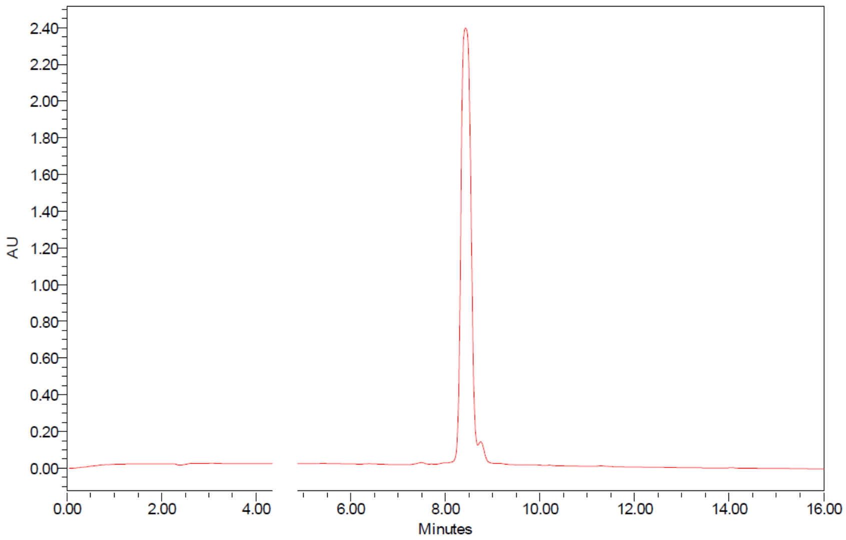

Ac-Met-Leu-Val-Ser-Arg-ACC

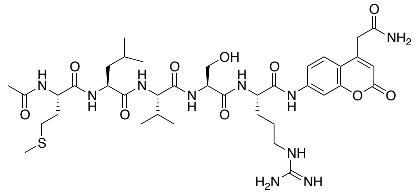

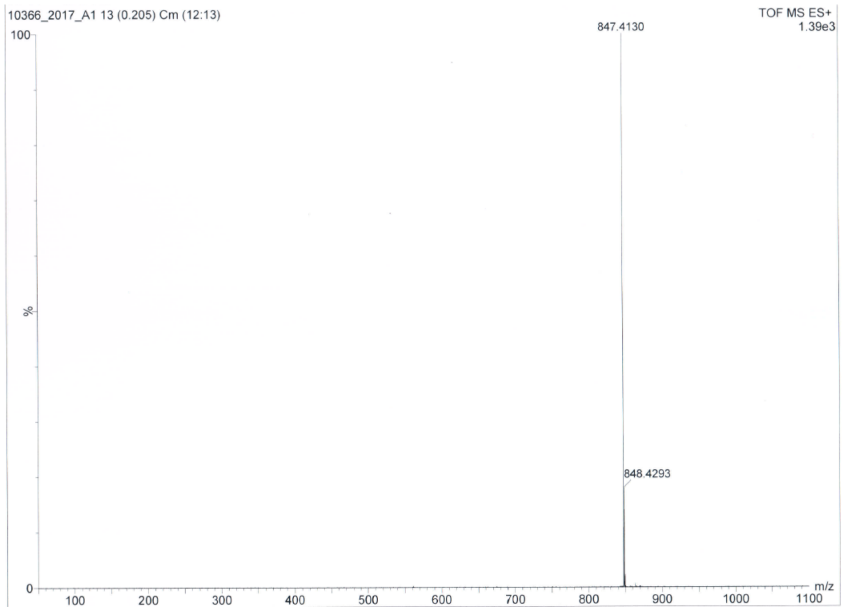

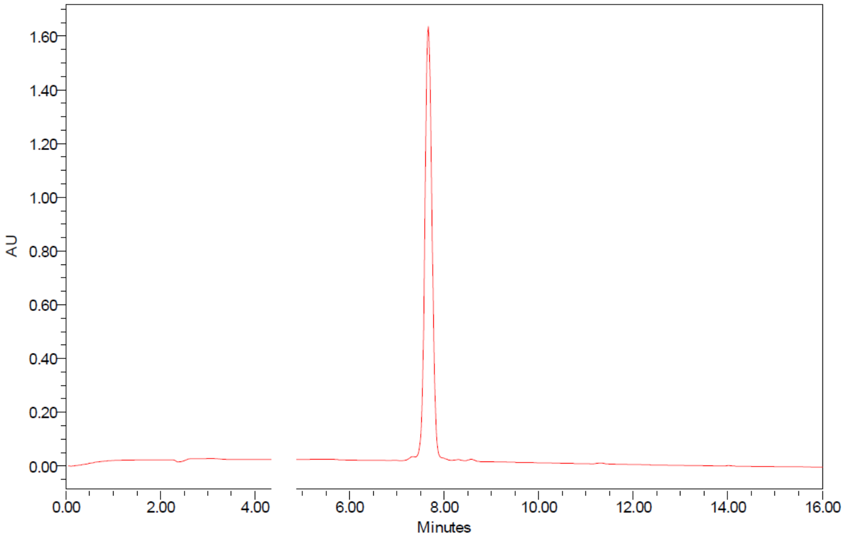

Ac-Ser-Leu-Val-Ser-Arg-ACC

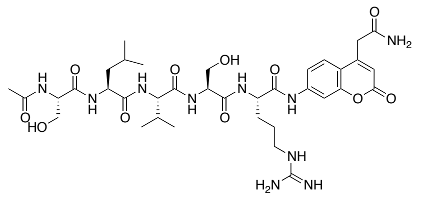

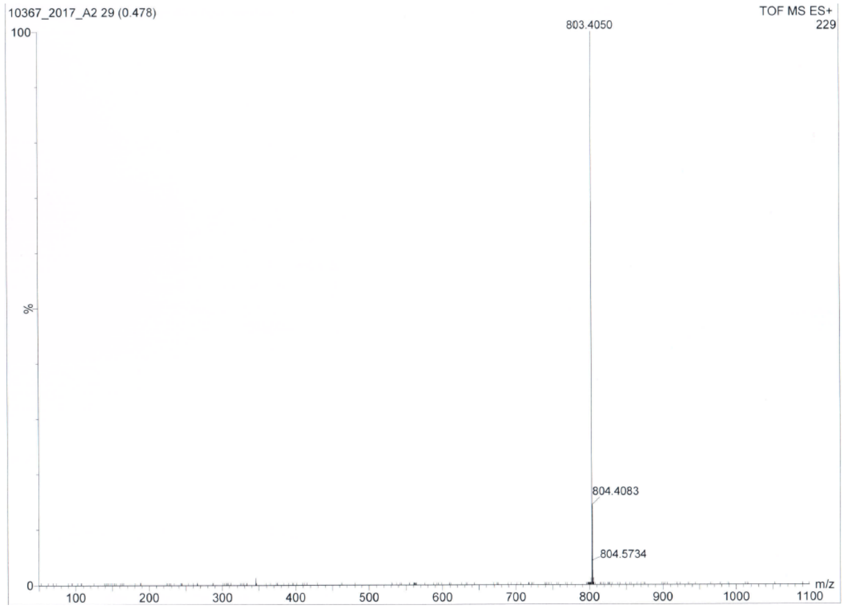

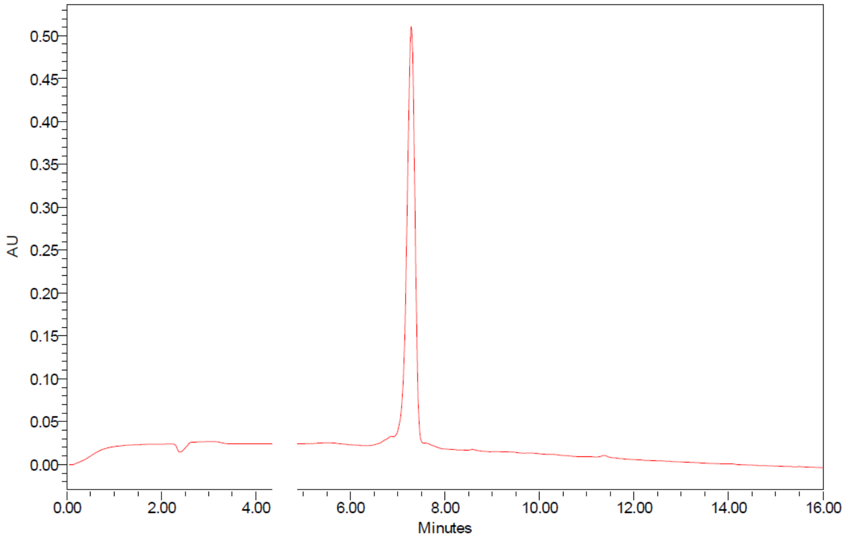

Ac-Thr-Leu-Val-Ser-Arg-ACC

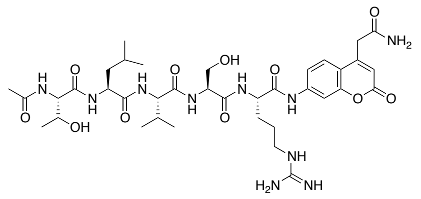

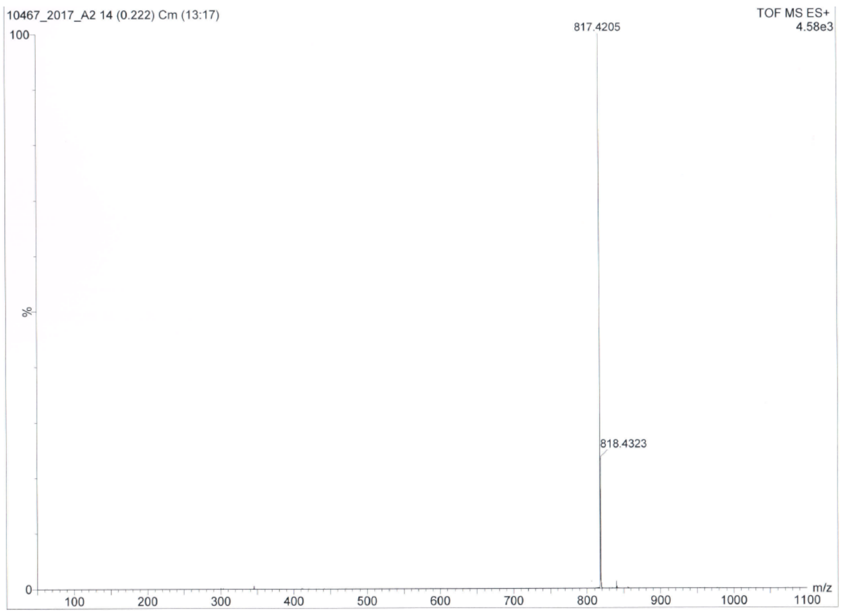

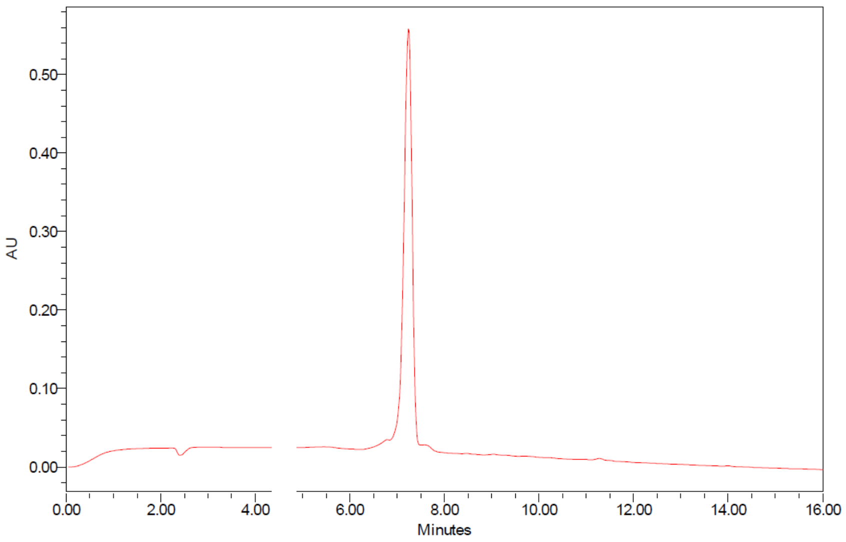

Ac-Arg-Leu-Val-Ser-Arg-ACC

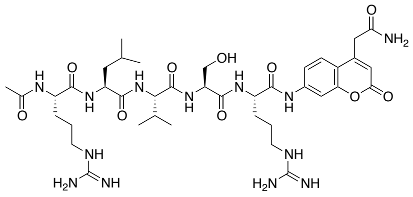

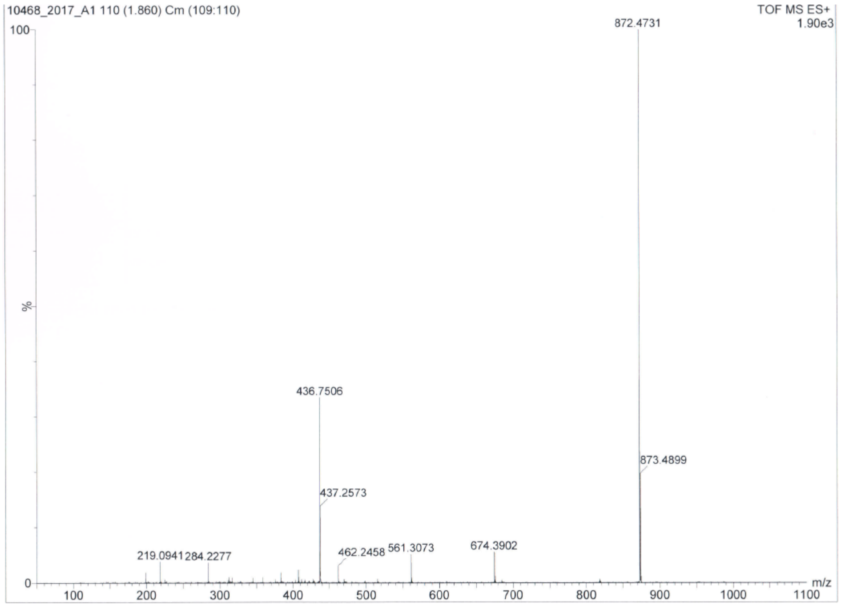

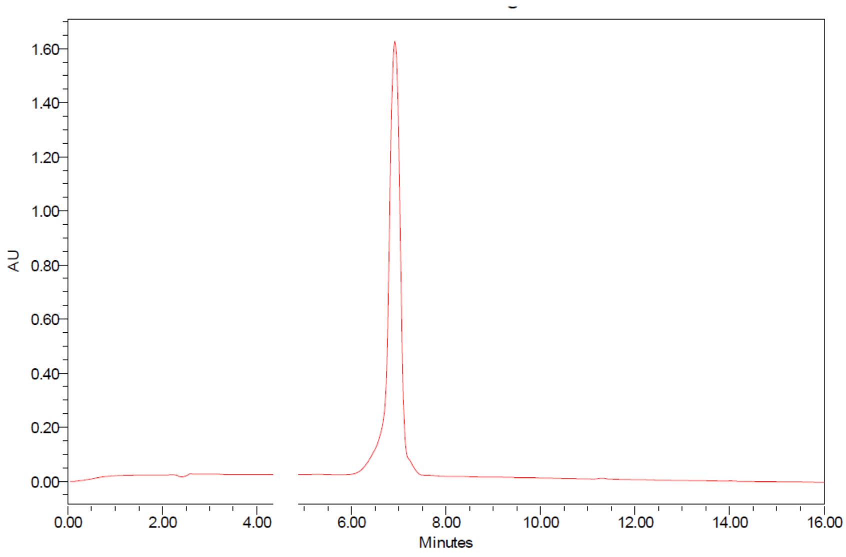

Ac-Lys-Leu-Val-Ser-Arg-ACC

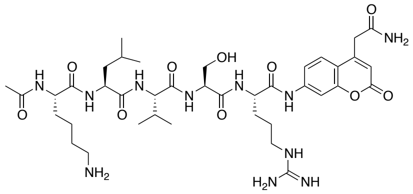

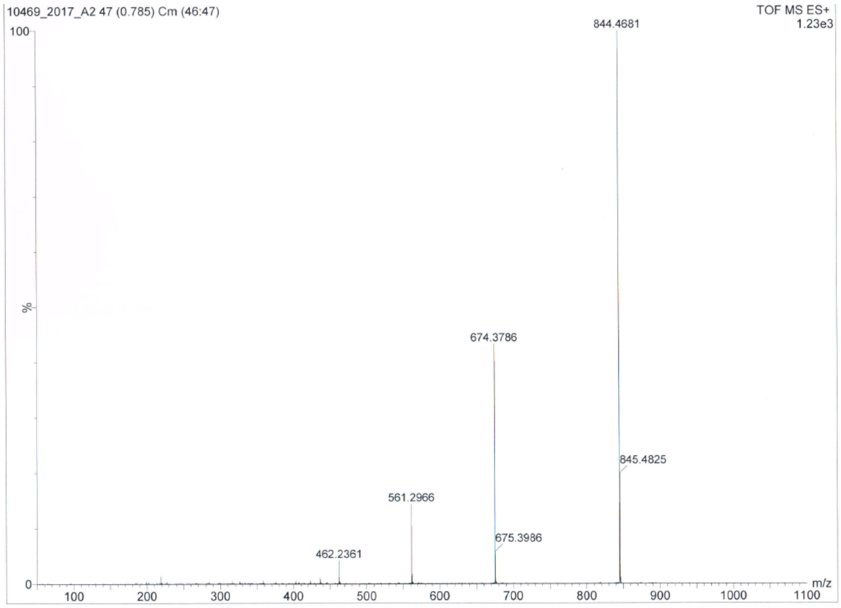

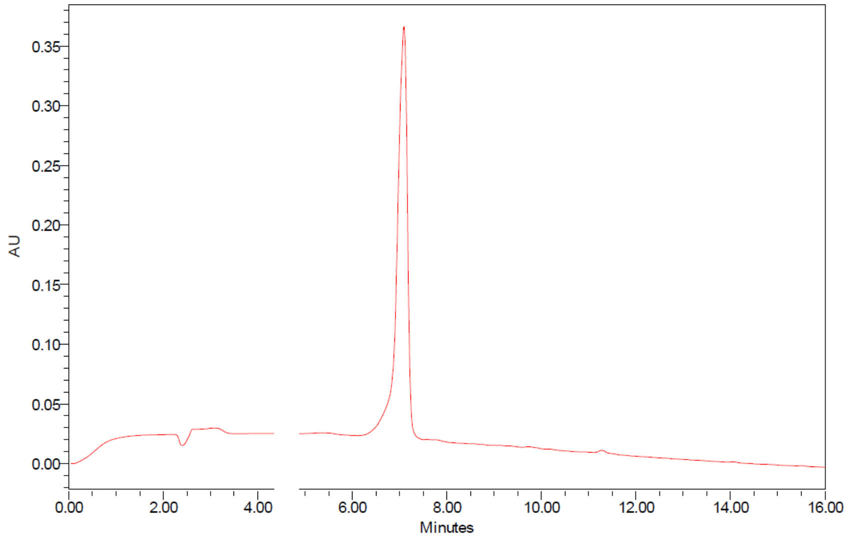

Ac-Asp-Leu-Val-Ser-Arg-ACC

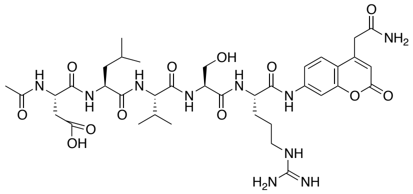

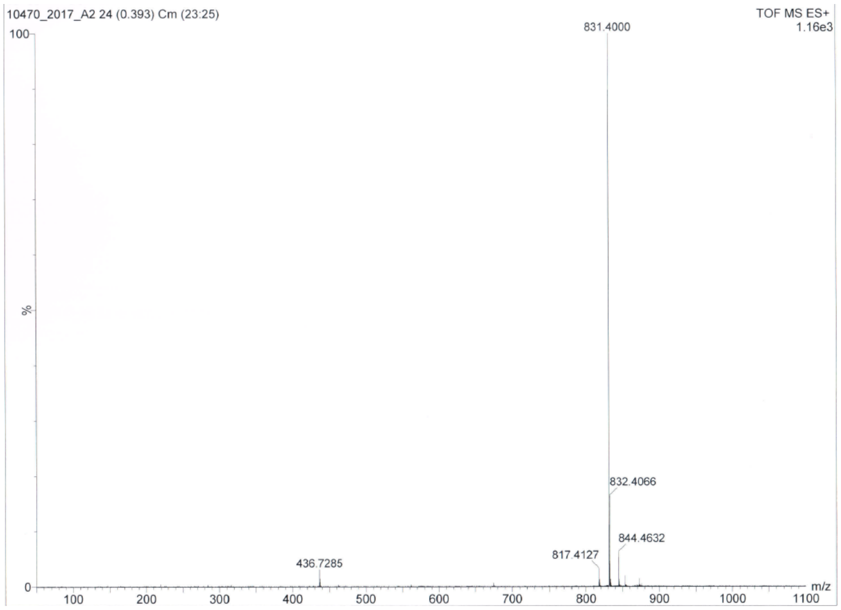

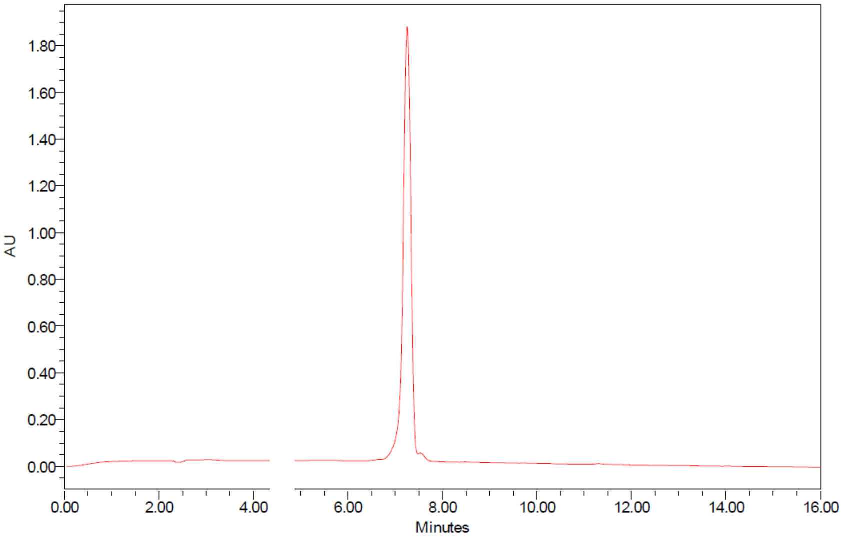

Ac-Glu-Leu-Val-Ser-Arg-ACC

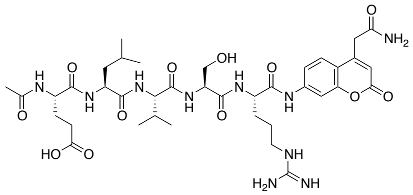

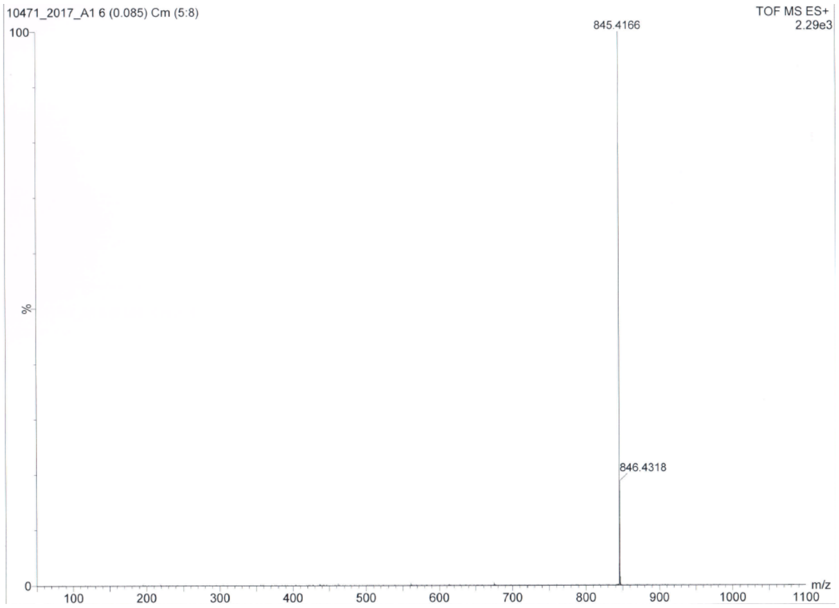

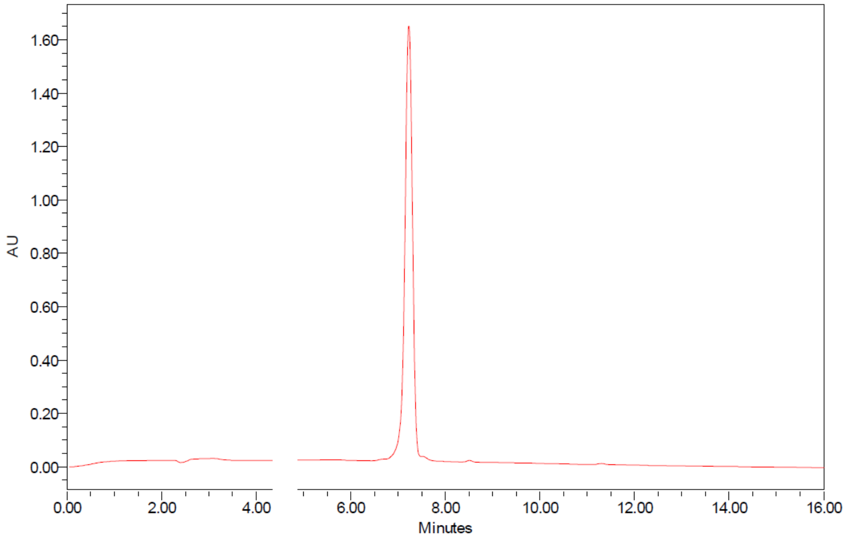

Ac-Asn-Leu-Val-Ser-Arg-ACC

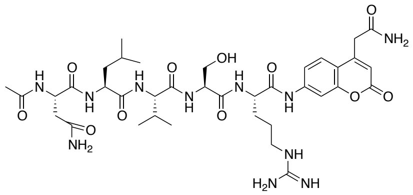

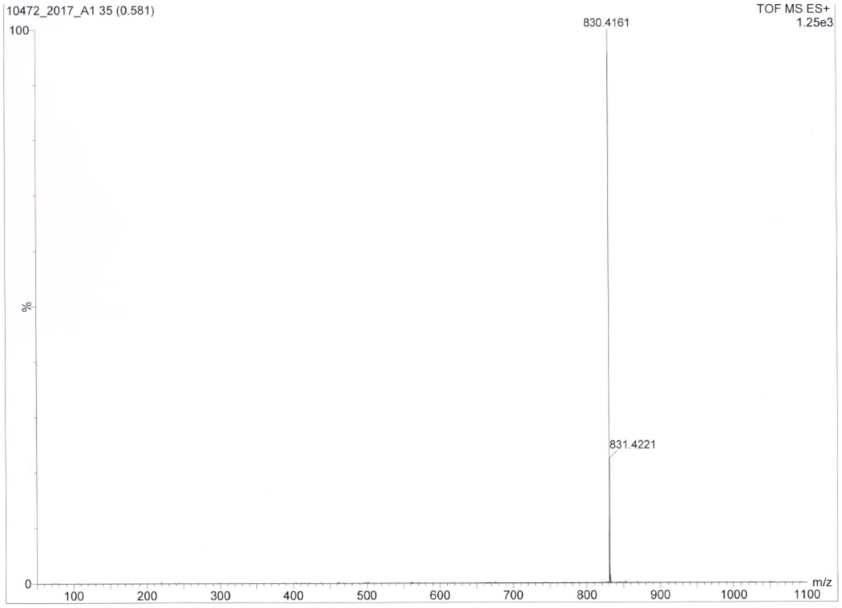

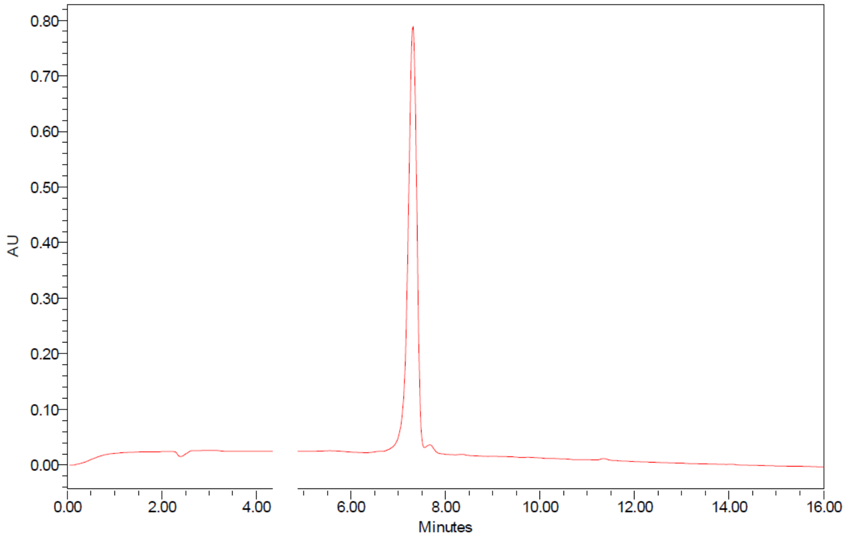

Ac-Gly-Leu-Val-Ser-Arg-ACC

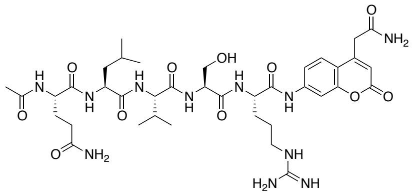

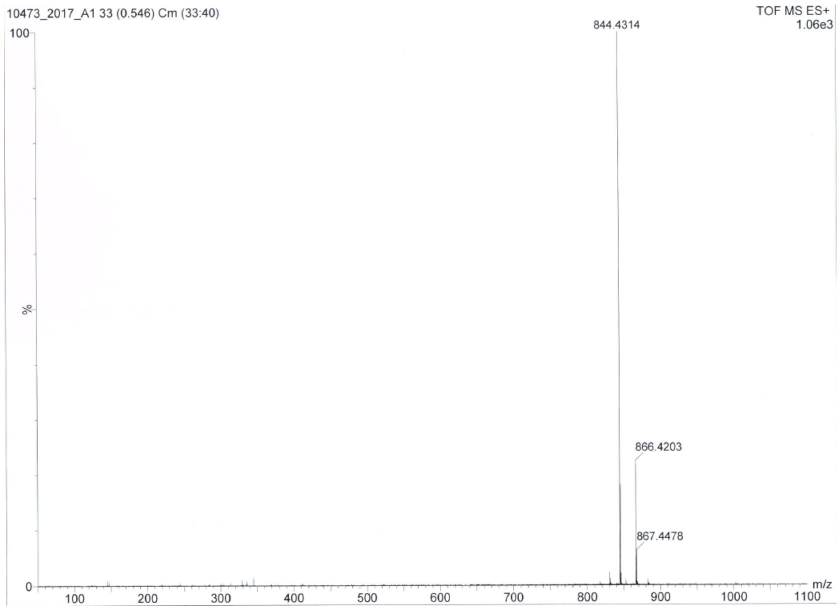

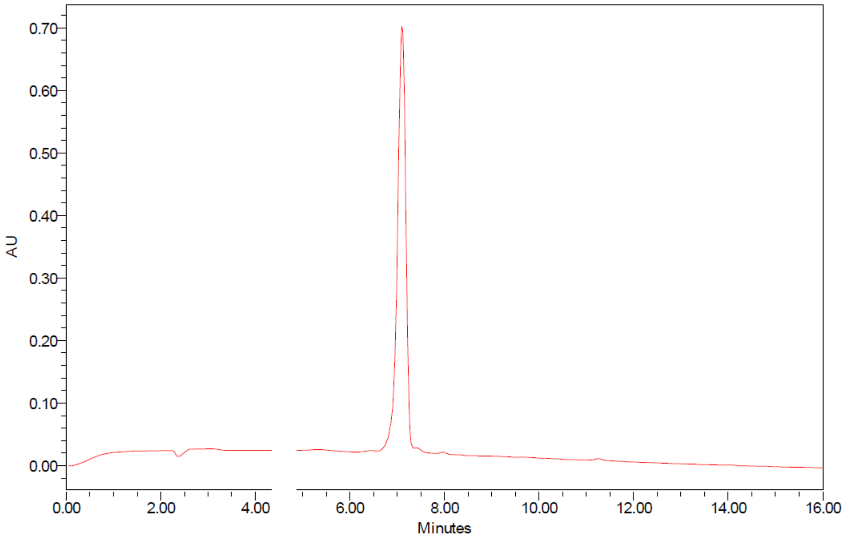

Ac-NLeu-Leu-Val-Ser-Arg-ACC

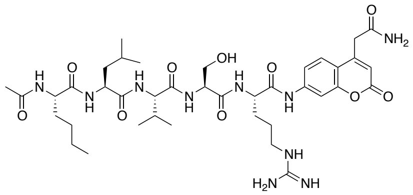

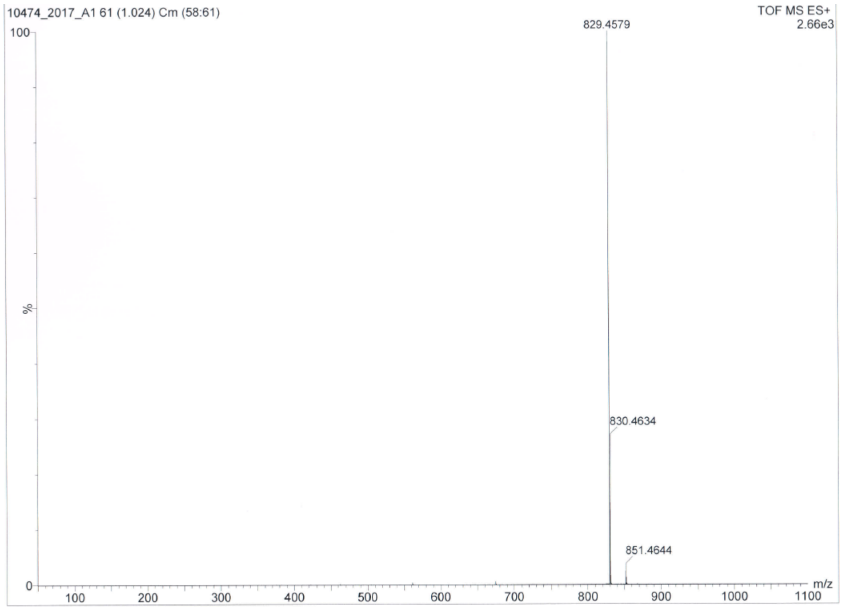

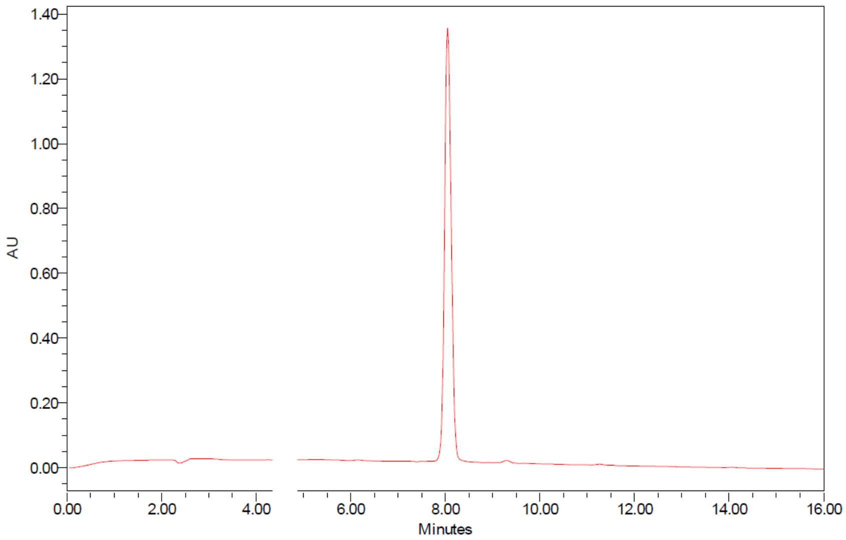

Ac-D-Ala-Leu-Val-Ser-Arg-ACC

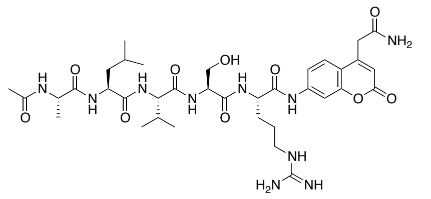

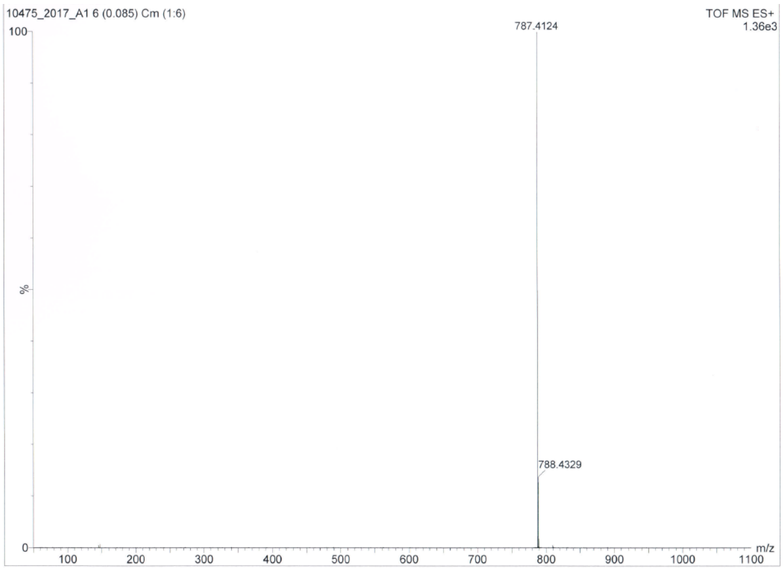

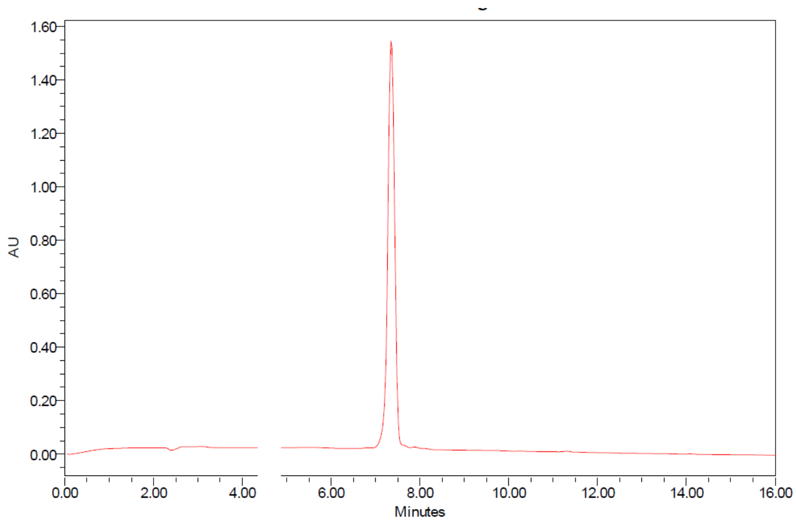

Ac-Abu-Leu-Val-Ser-Arg-ACC

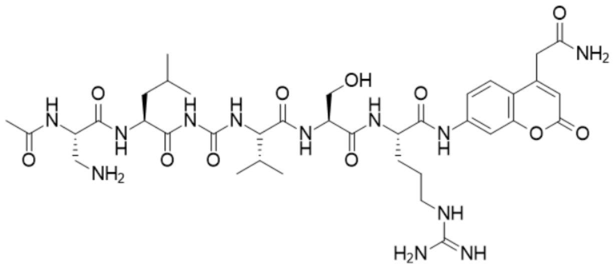

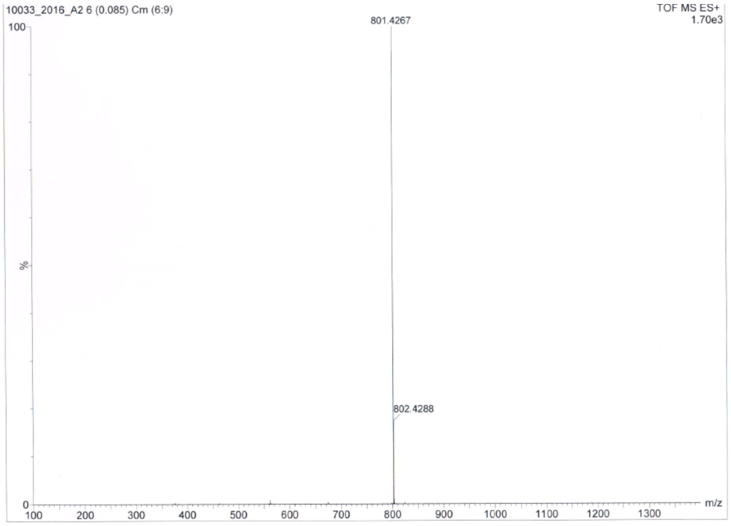

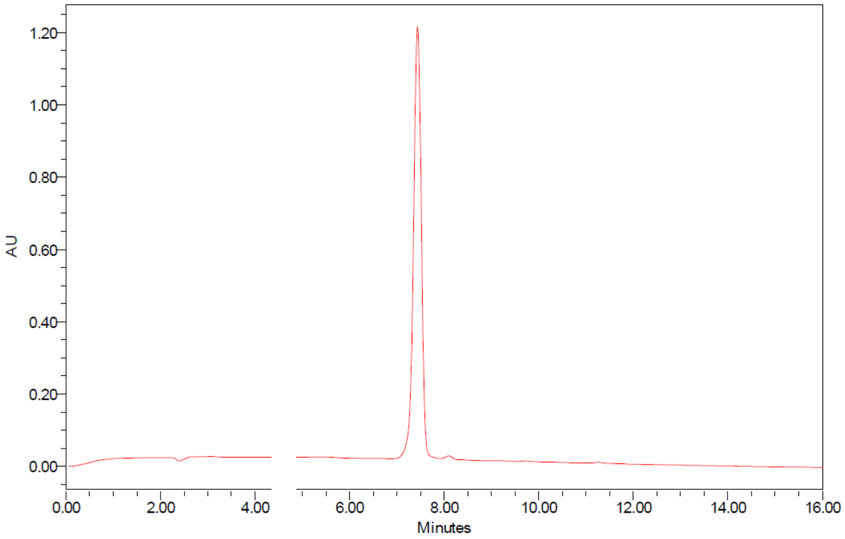

Ac-D-Val-Leu-Val-Ser-Arg-ACC

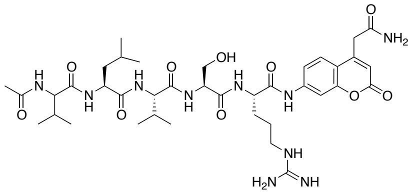

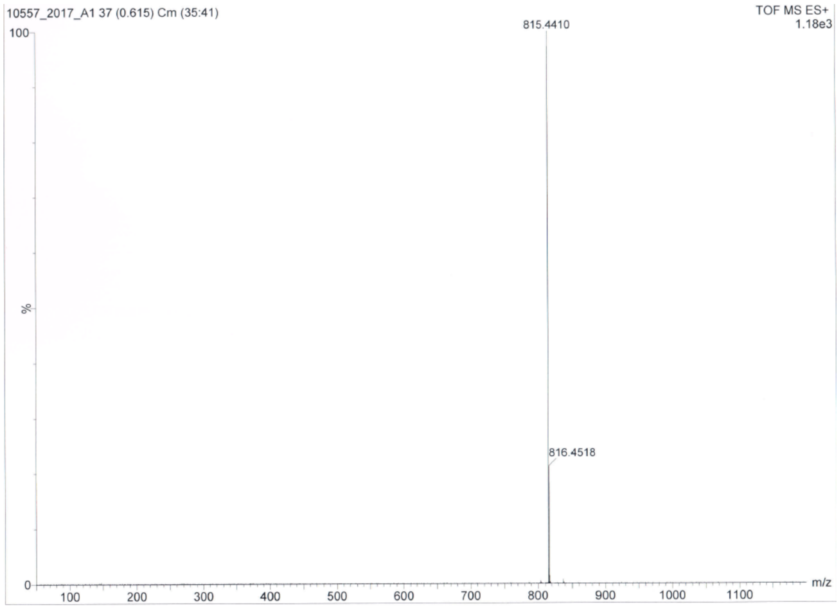

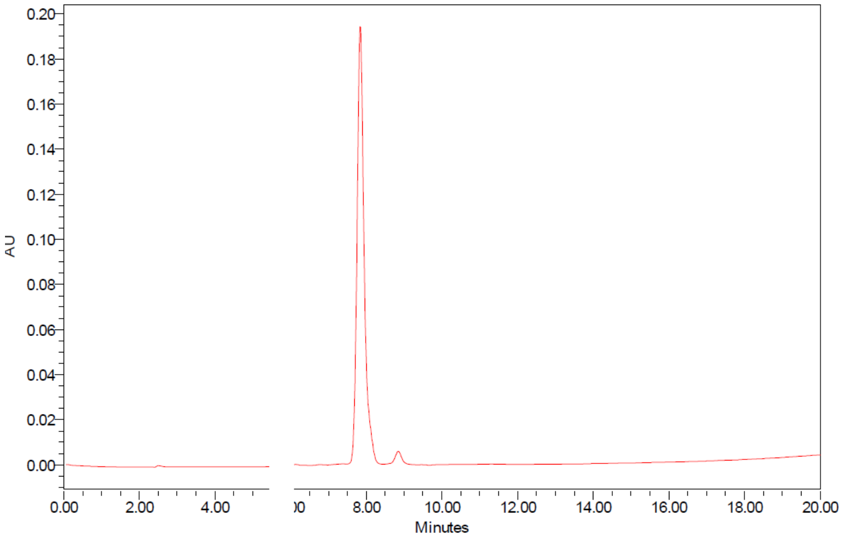

Ac-D-Leu-Leu-Val-Ser-Arg-ACC

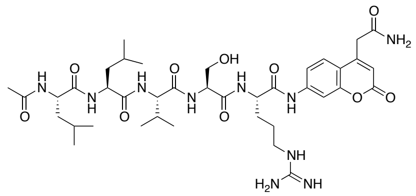

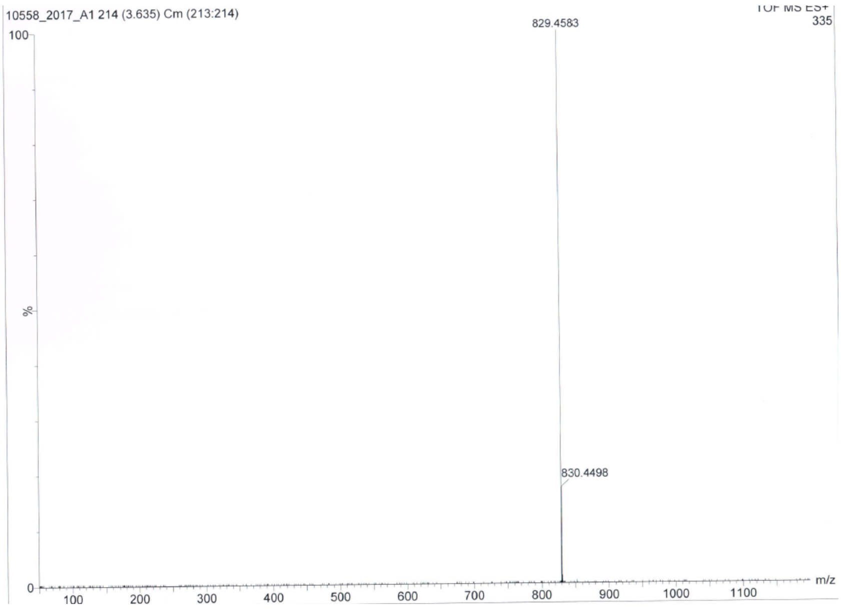

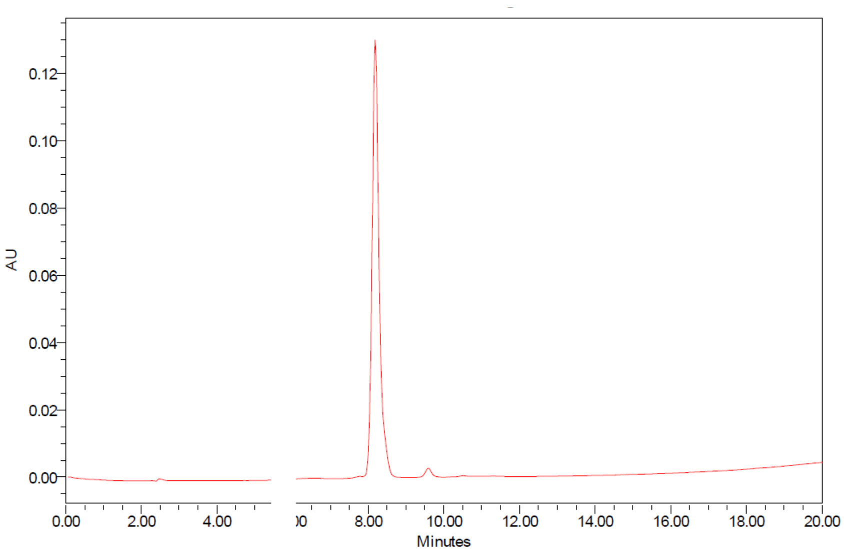

Ac-β-Ala-Leu-Val-Ser-Arg-ACC

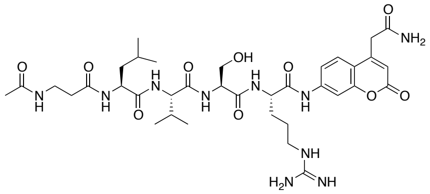

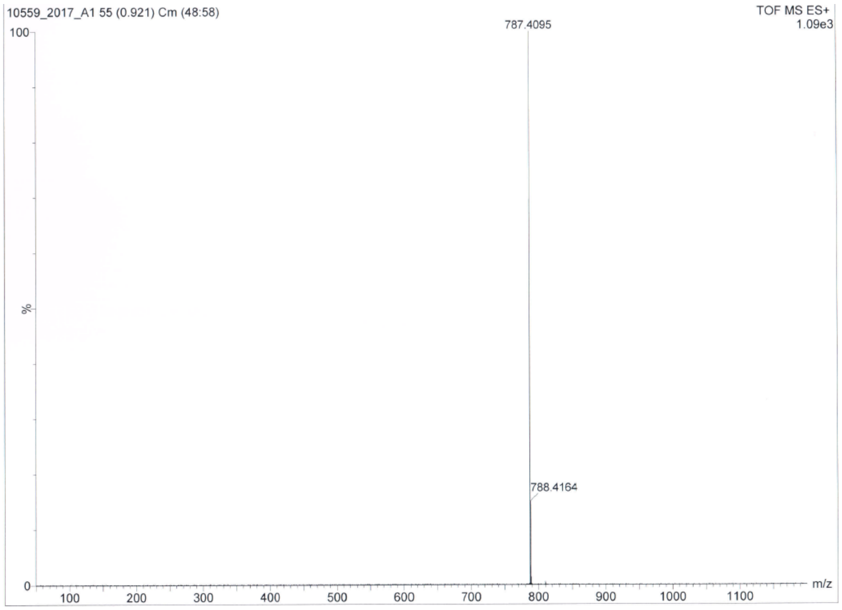

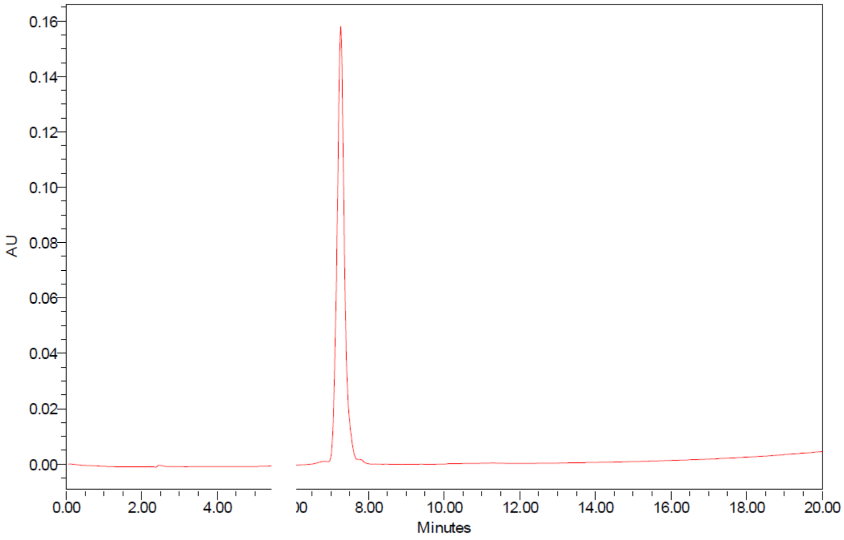

Ac-Phe(guan)-Leu-Val-Ser-Arg-ACC

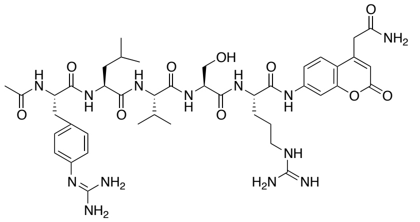

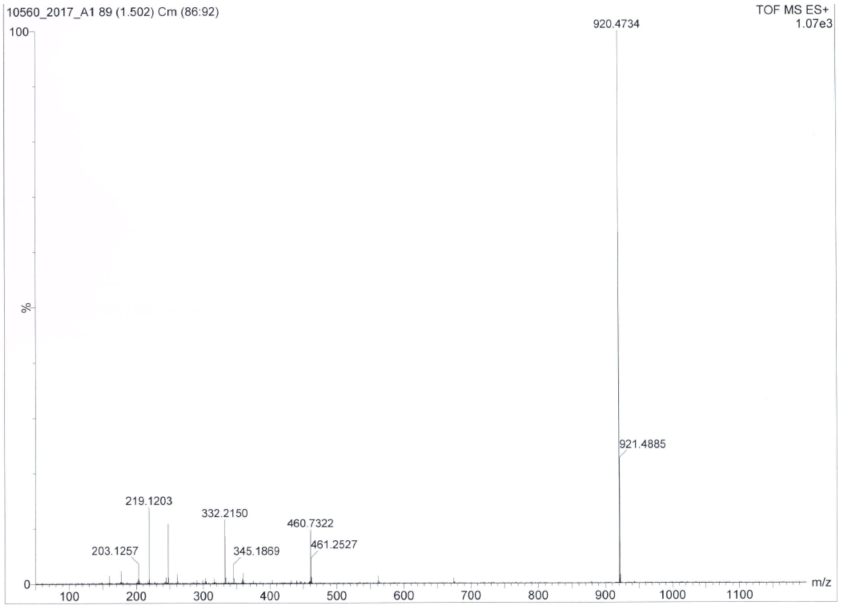

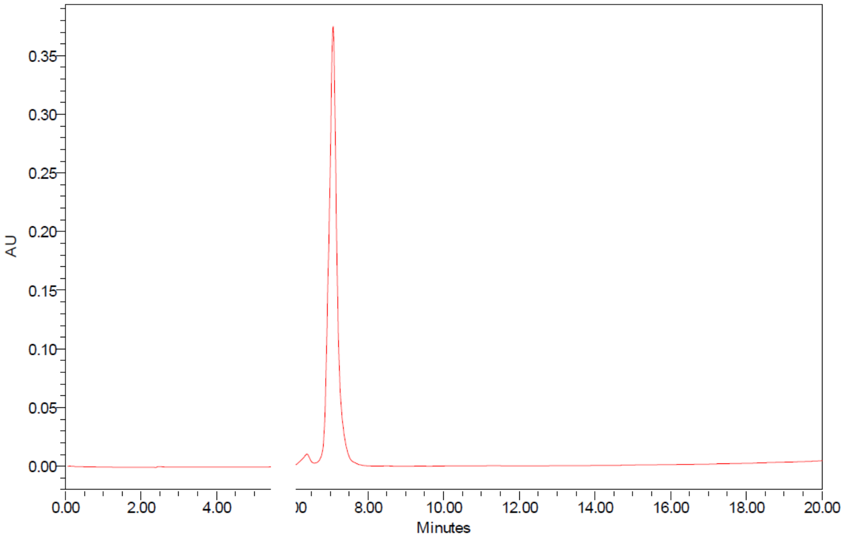

Ac-hTyr(Me)-Leu-Val-Ser-Arg-ACC

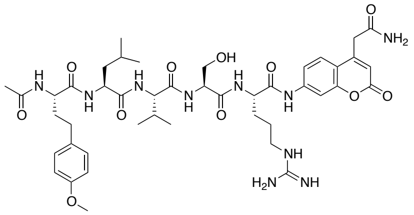

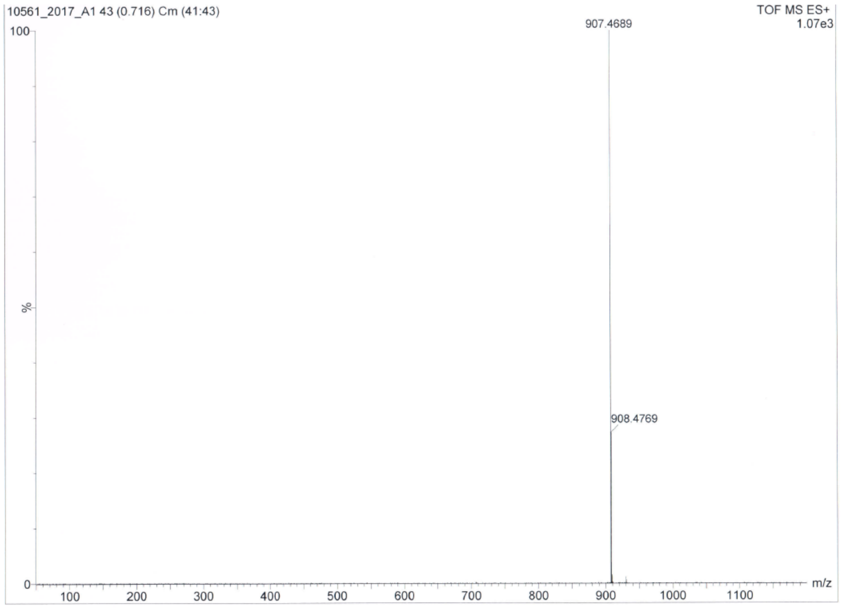

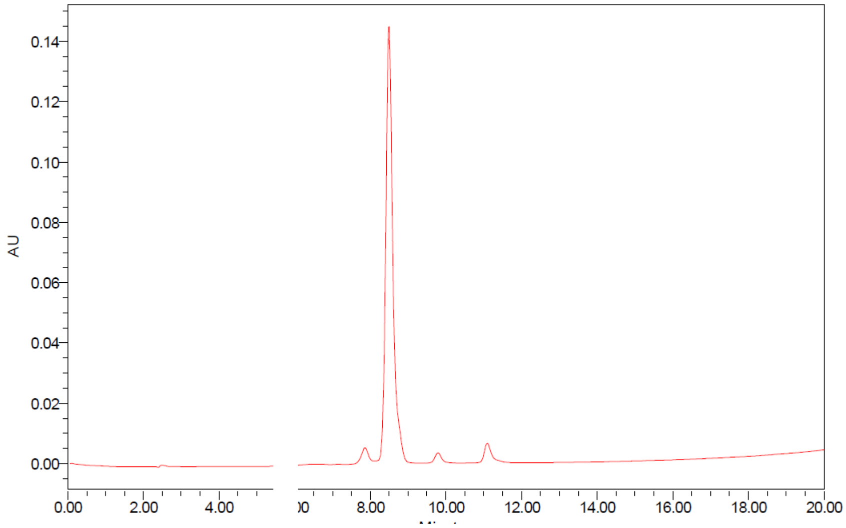

Ac-ChA-Leu-Val-Ser-Arg-ACC

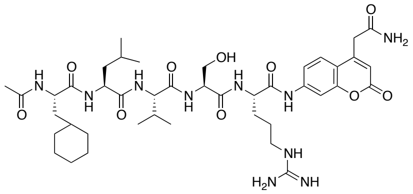

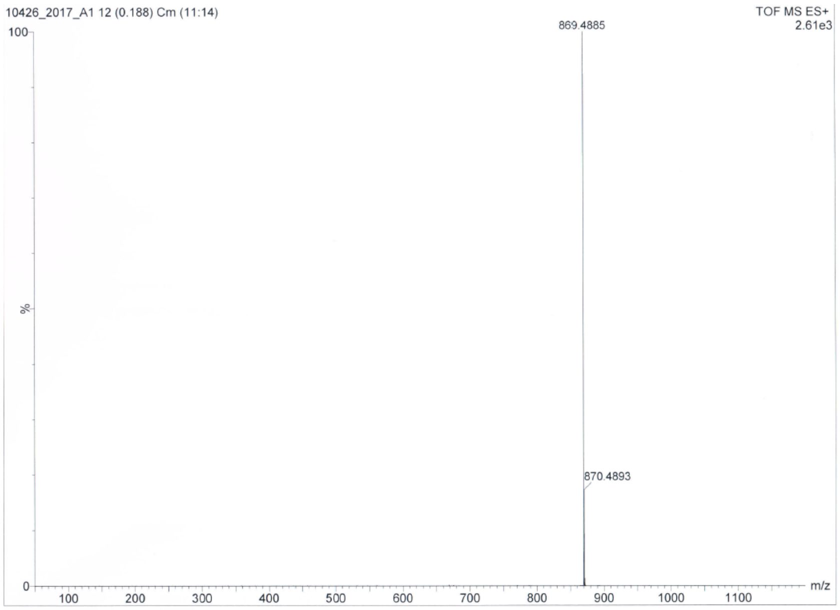

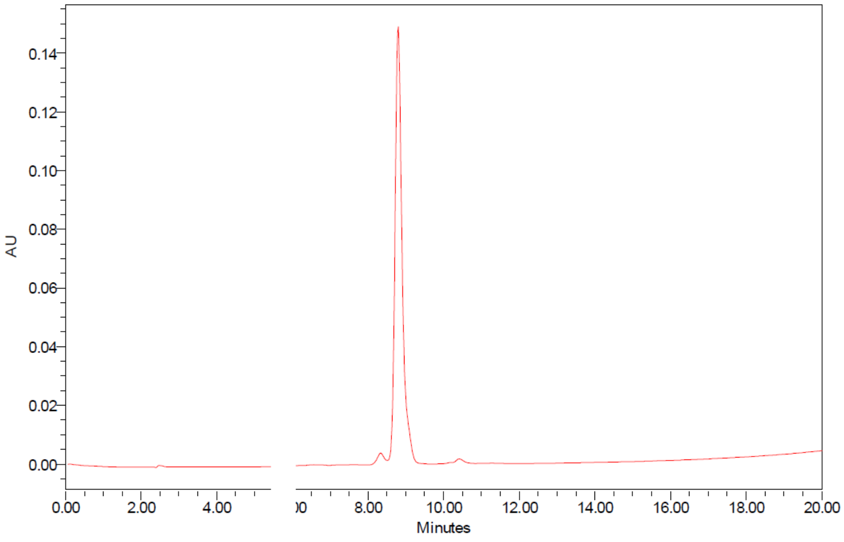

Ac-His(Bzl)-Leu-Val-Ser-Arg-ACC

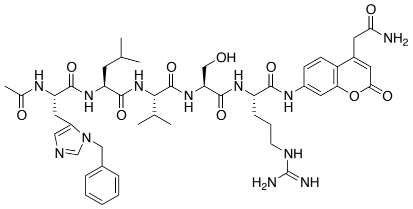

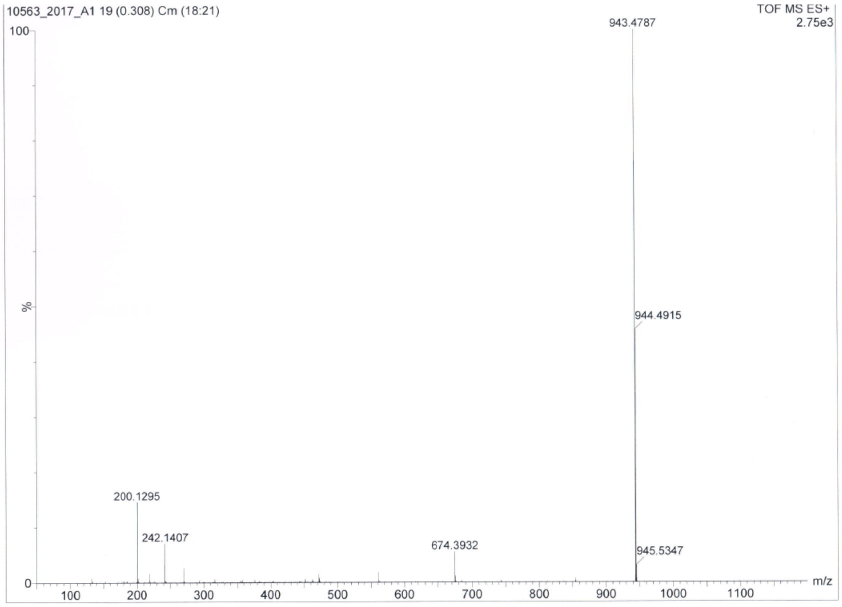

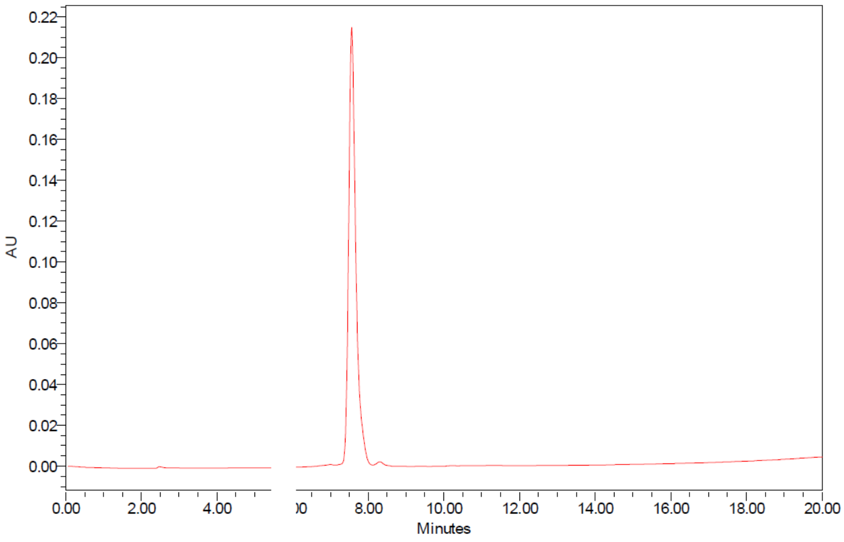

Ac-hArg-Leu-Val-Ser-Arg-ACC

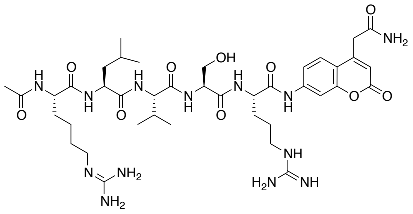

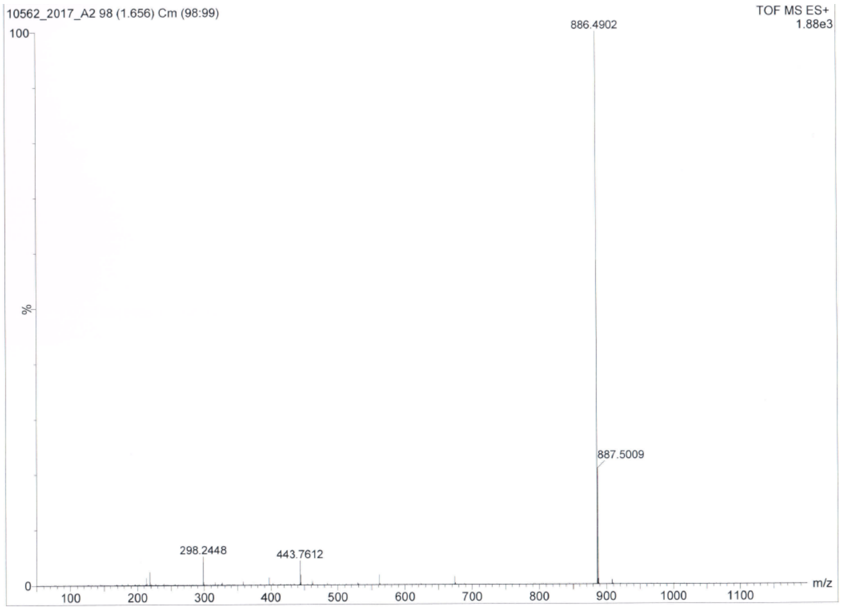

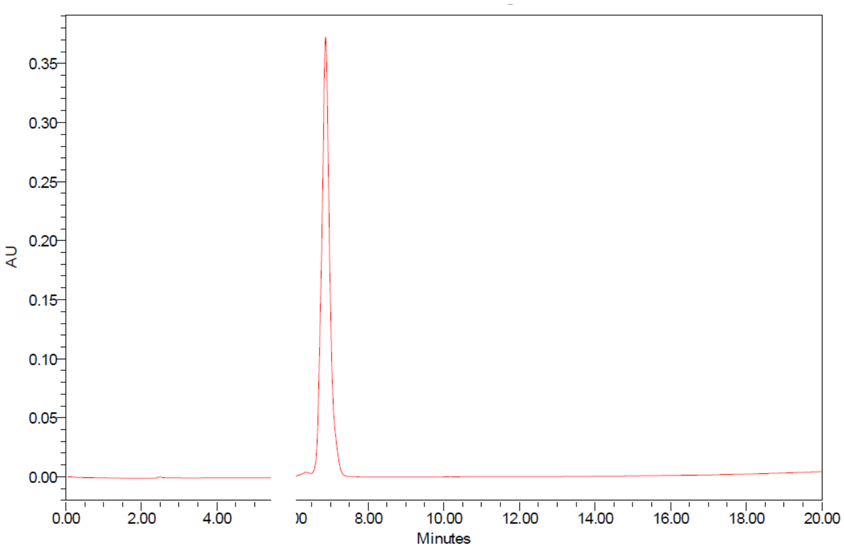

Ac-Agp-Leu-Val-Ser-Arg-ACC

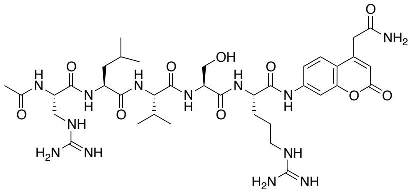

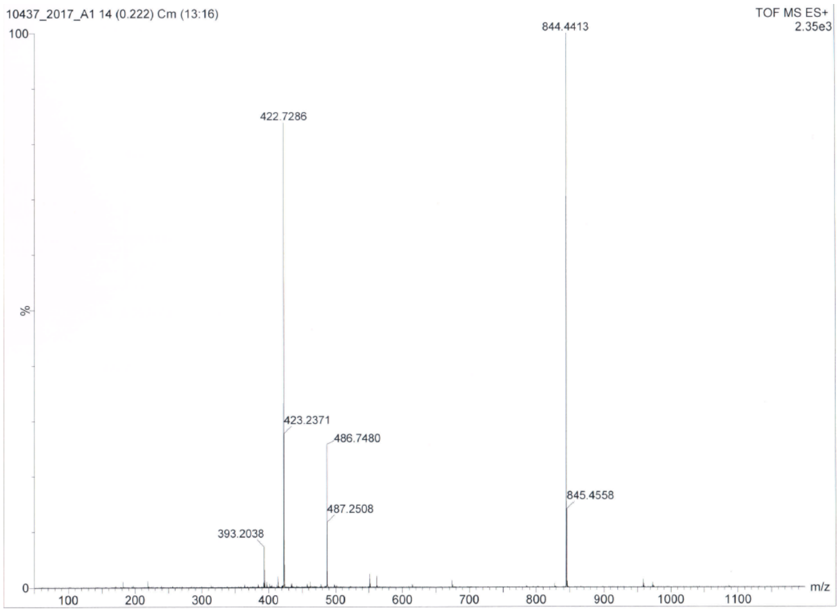

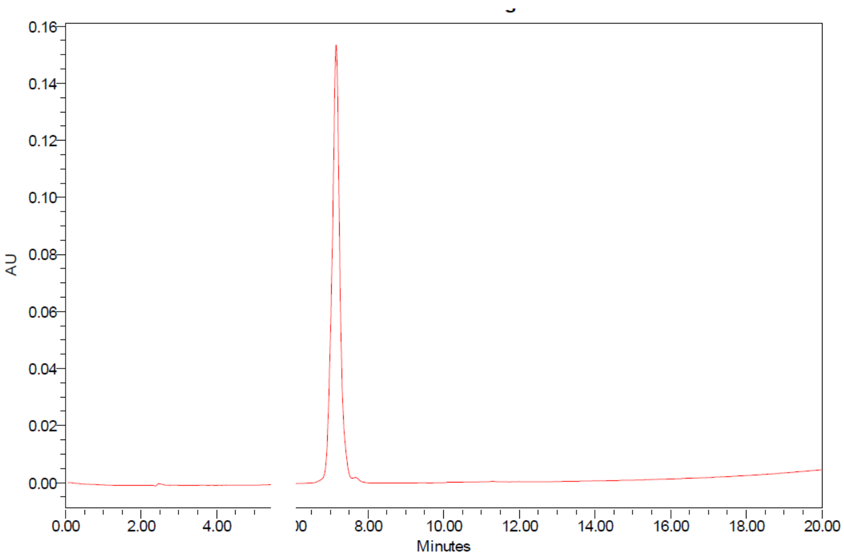

Ac-Lys(2-ClZ)-Leu-Val-Ser-Arg-ACC

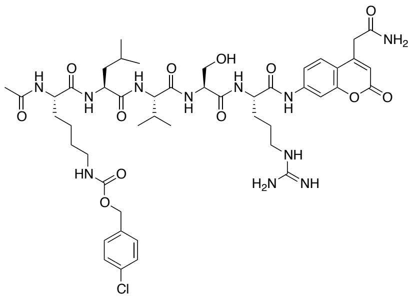

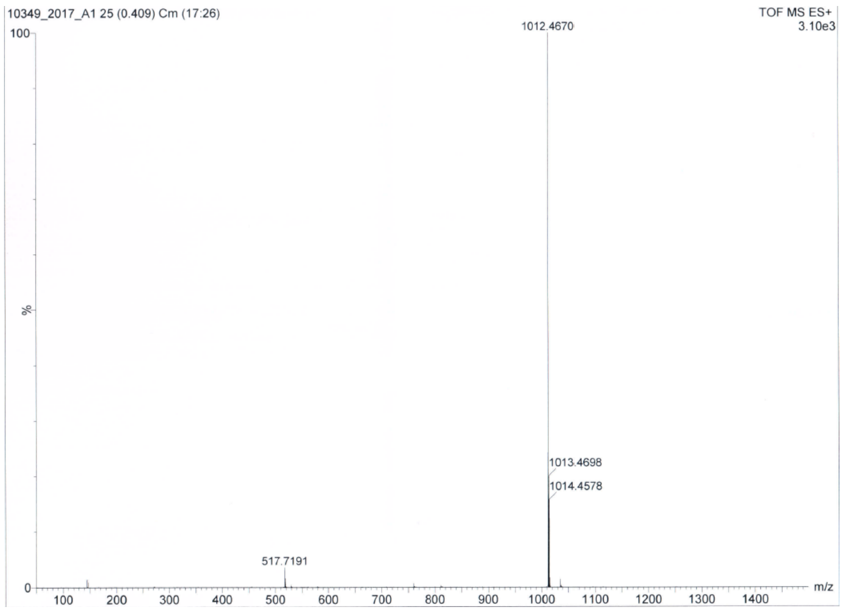

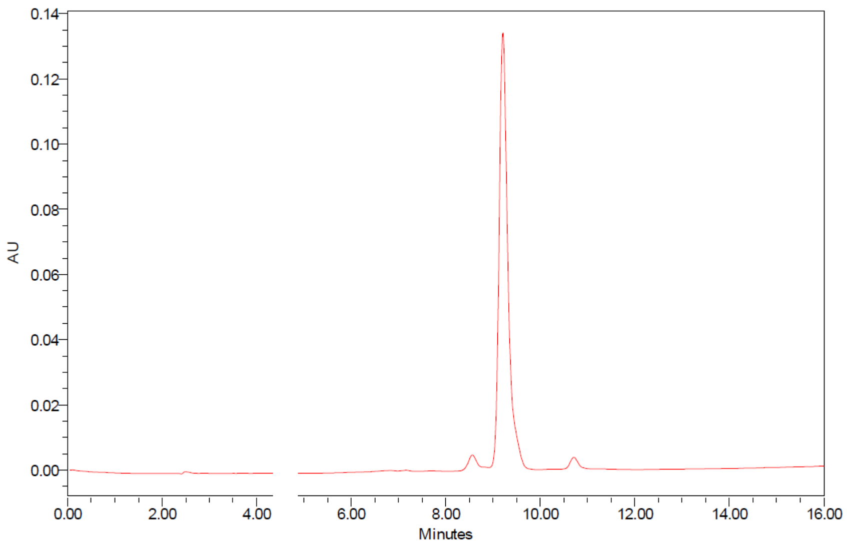

Ac-Lys(Ac)-Leu-Val-Ser-Arg-ACC

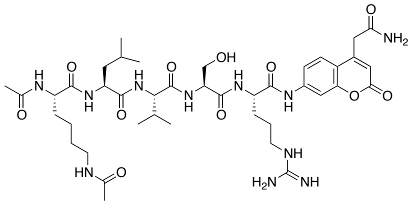

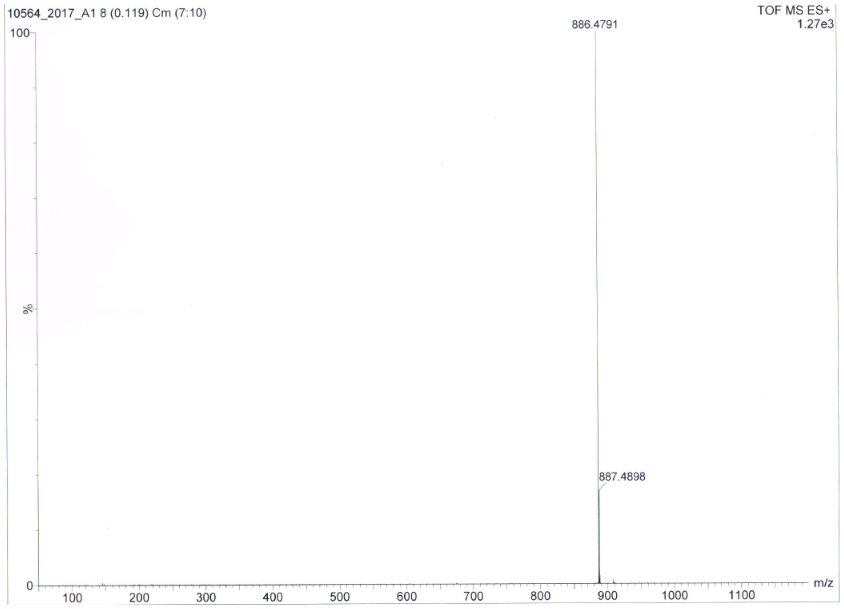

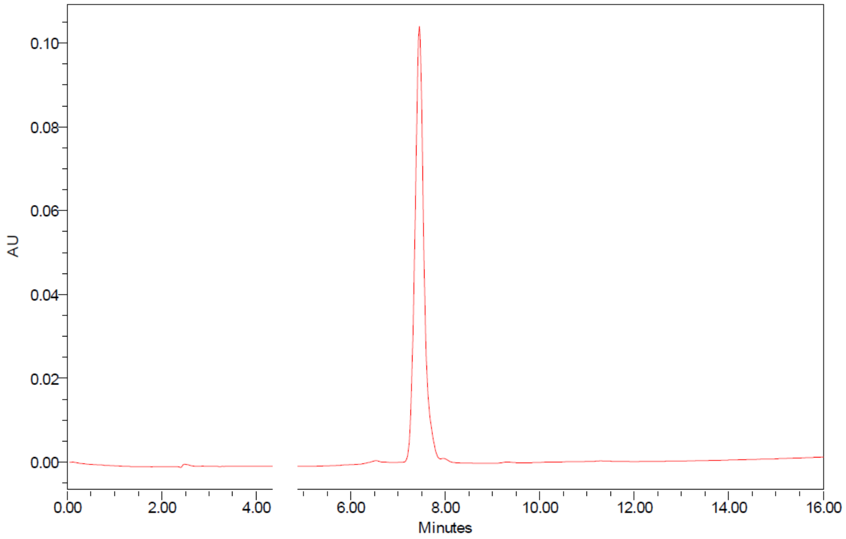

Ac-Lys(TFA)-Leu-Val-Ser-Arg-ACC

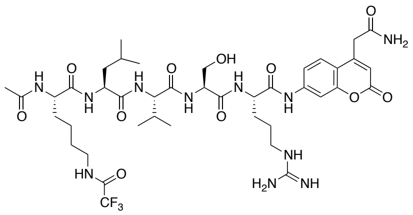

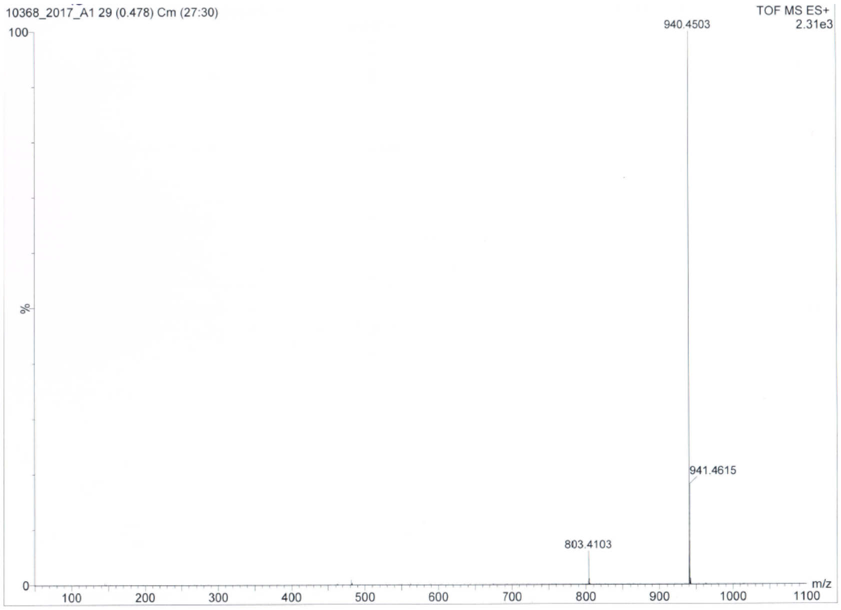

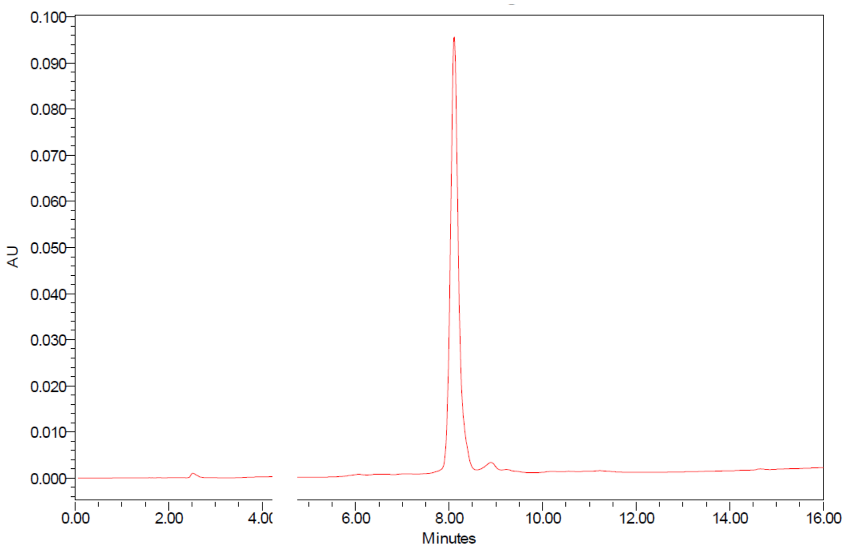

Ac-Orn-Leu-Val-Ser-Arg-ACC

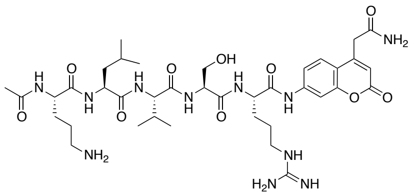

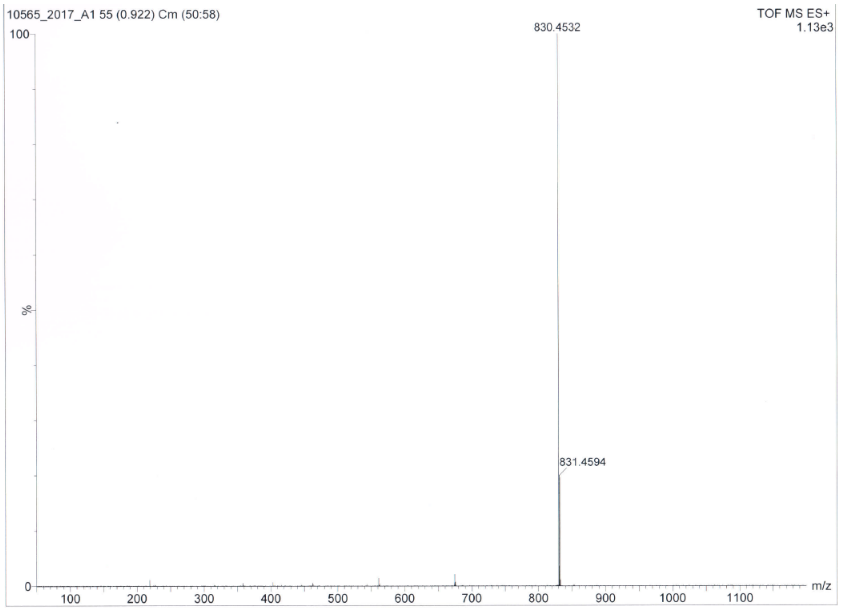

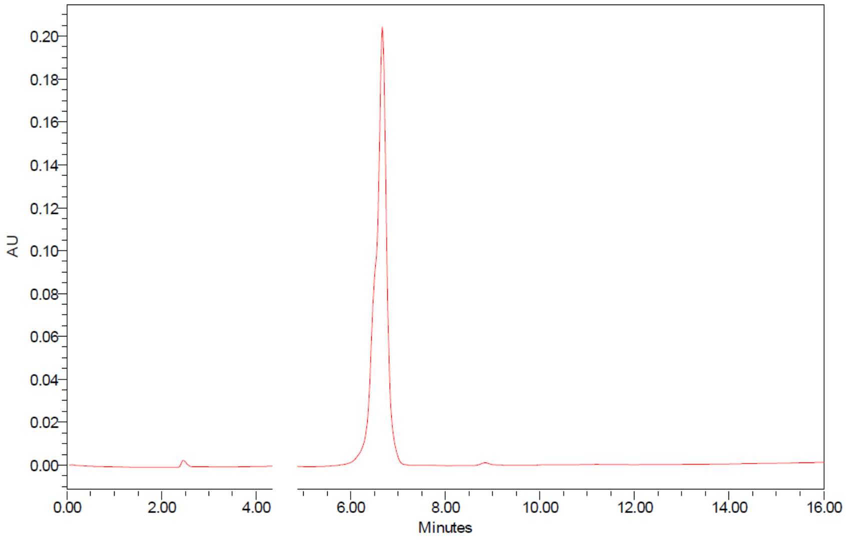

Ac-hCit-Leu-Val-Ser-Arg-ACC

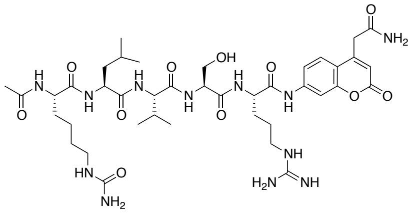

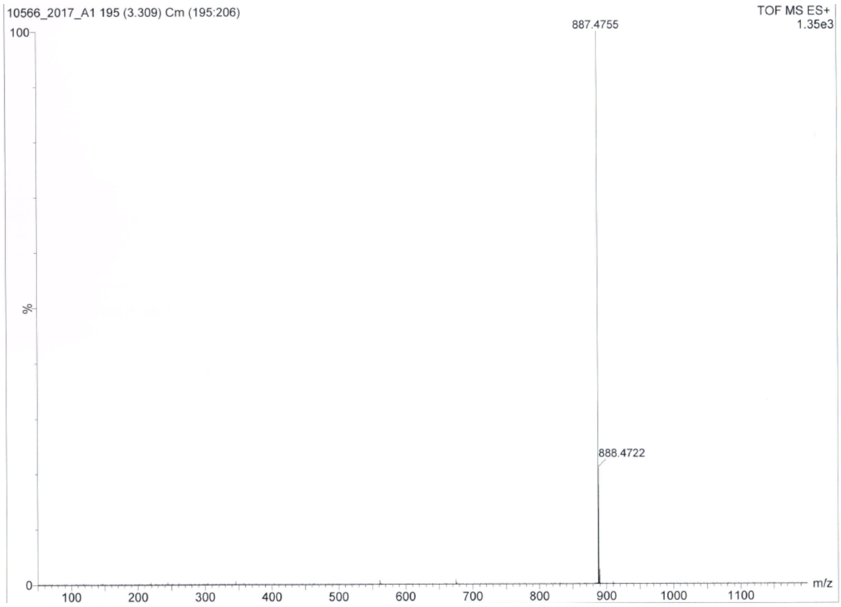

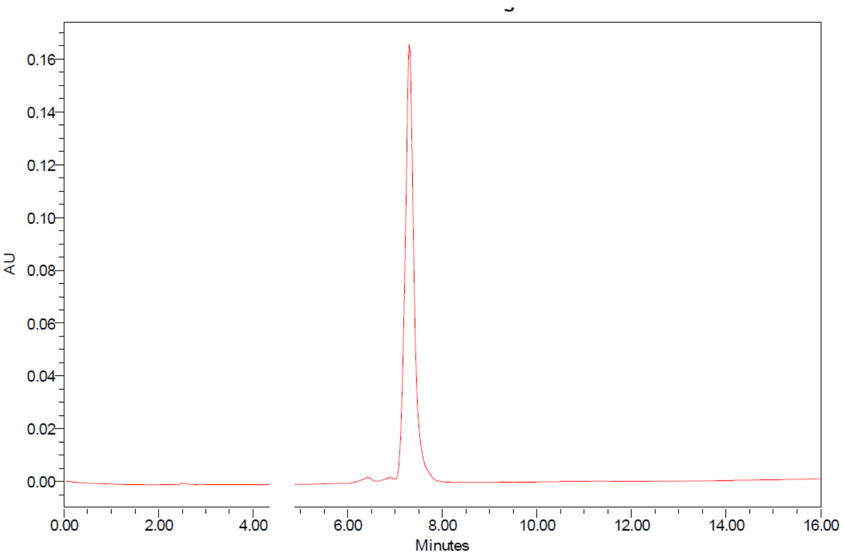

Ac-Cit-Leu-Val-Ser-Arg-ACC

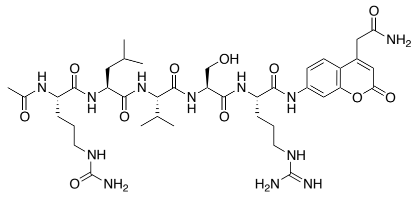

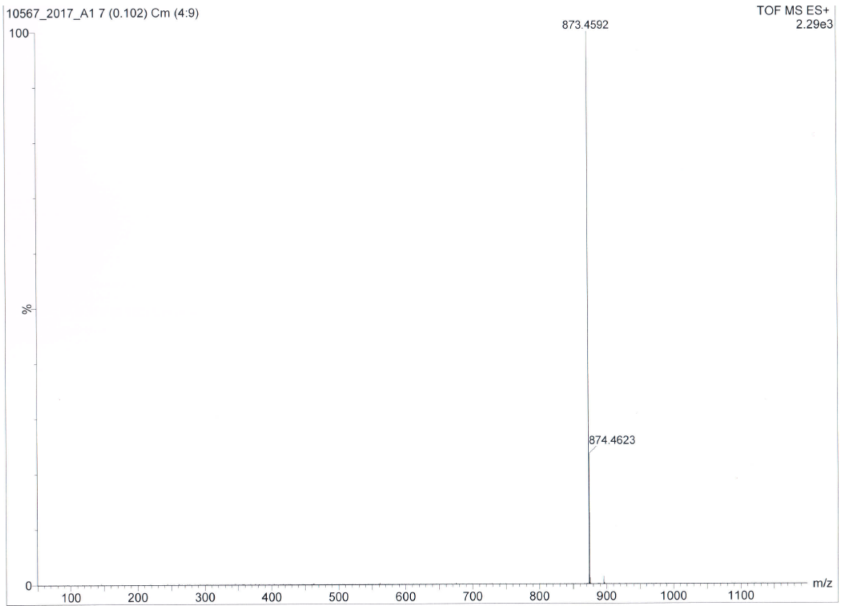

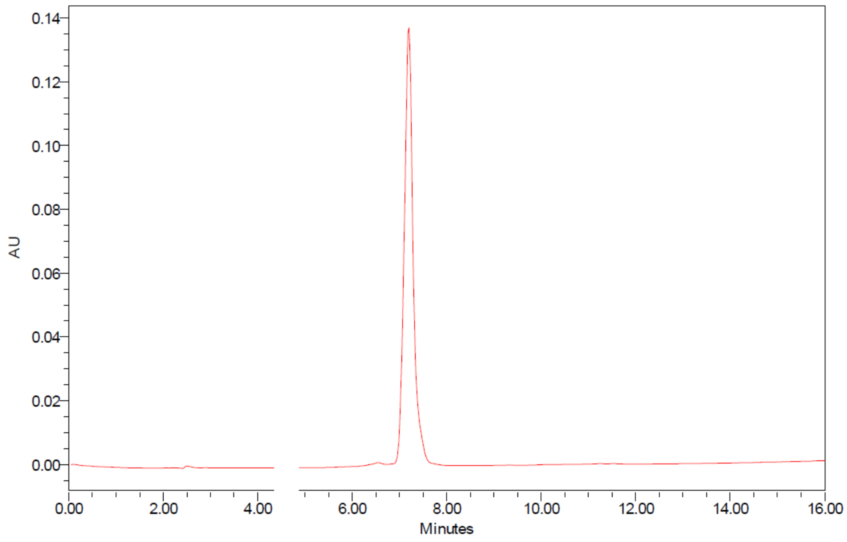

Ac-Dab(Z)-Leu-Val-Ser-Arg-ACC

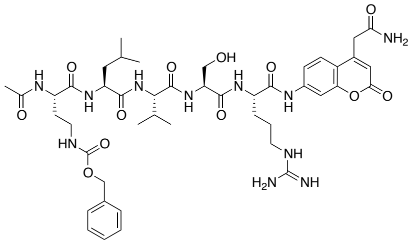

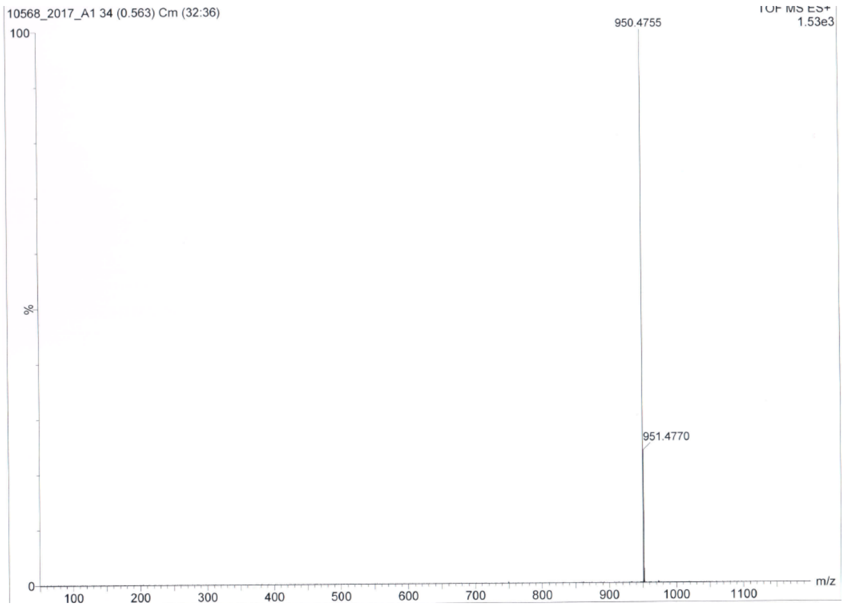

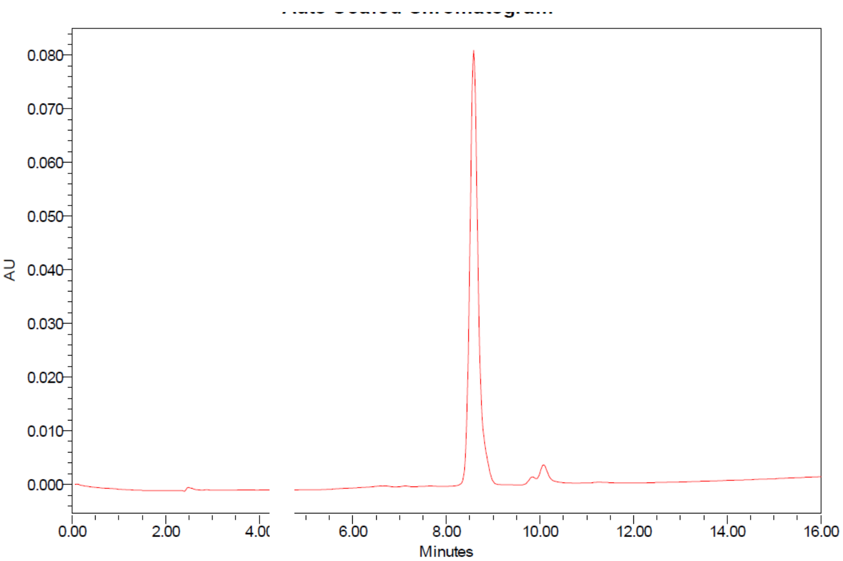

Ac-Dab-Leu-Val-Ser-Arg-ACC

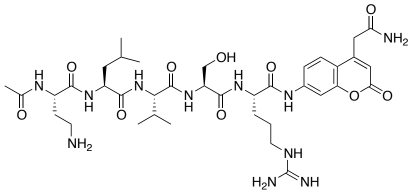

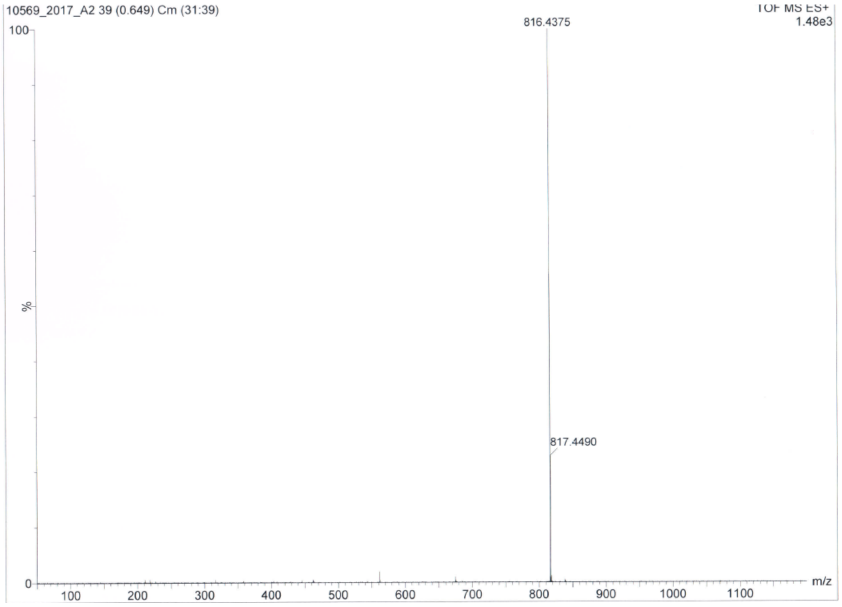

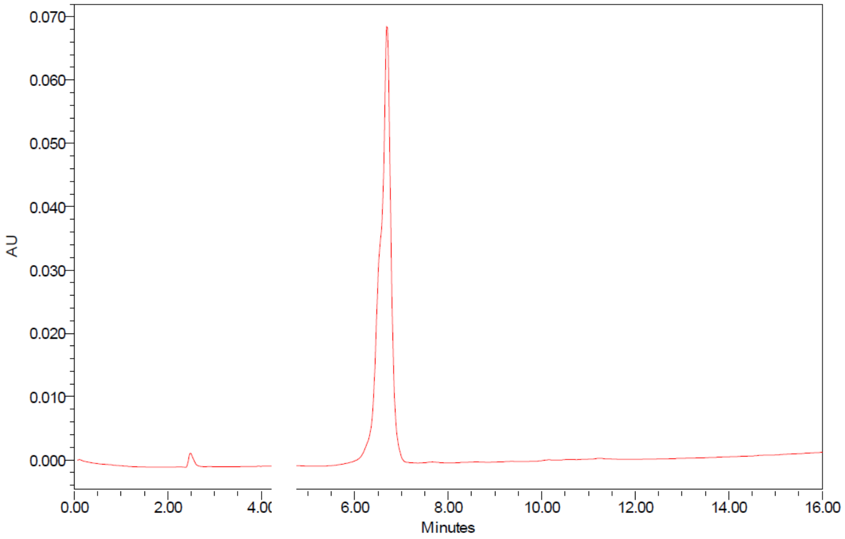

Ac-Dap-Leu-Val-Ser-Arg-ACC

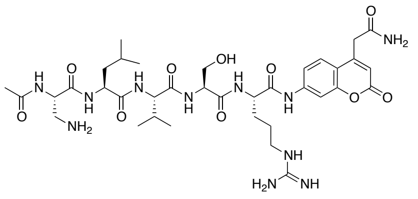

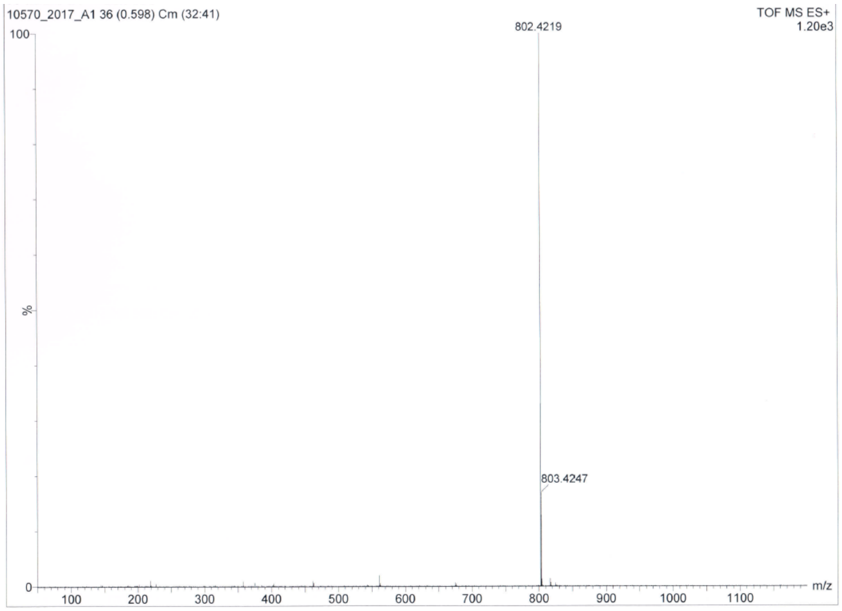

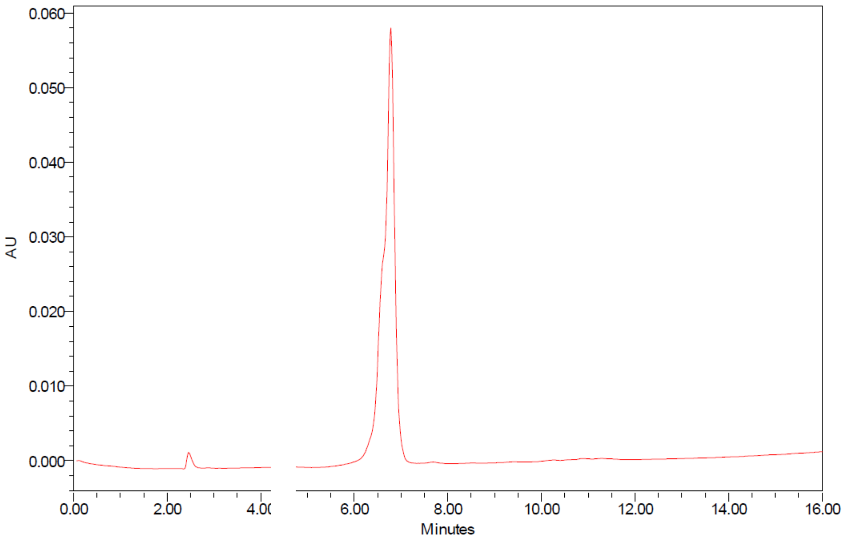

PKKG3 Ac-Ala-2-Aoc-Chg-Ser-Arg-ACC

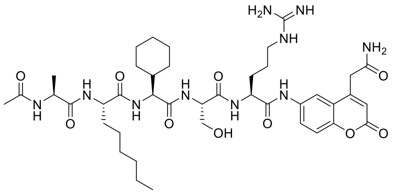

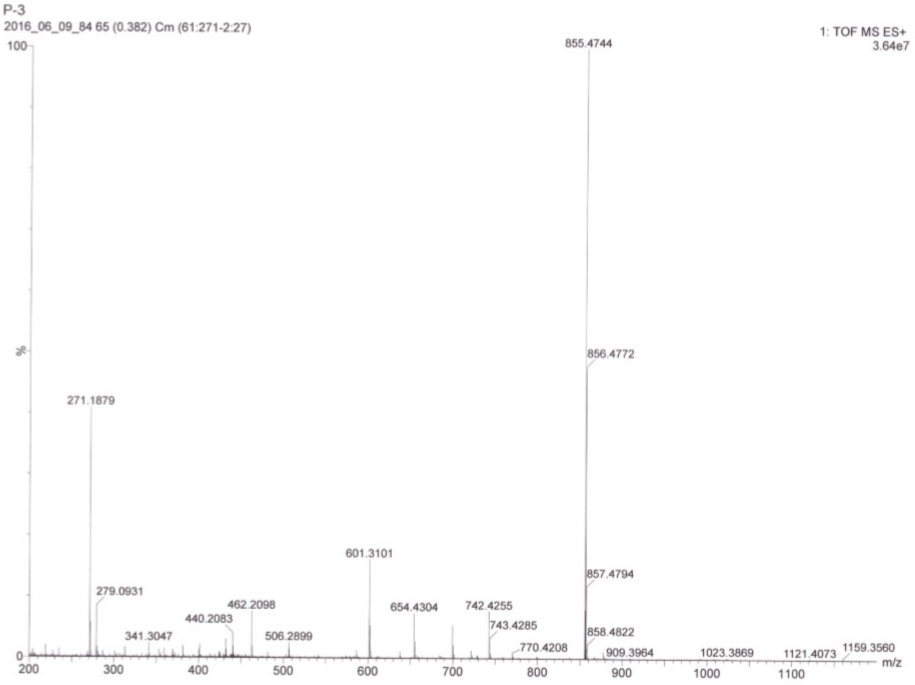

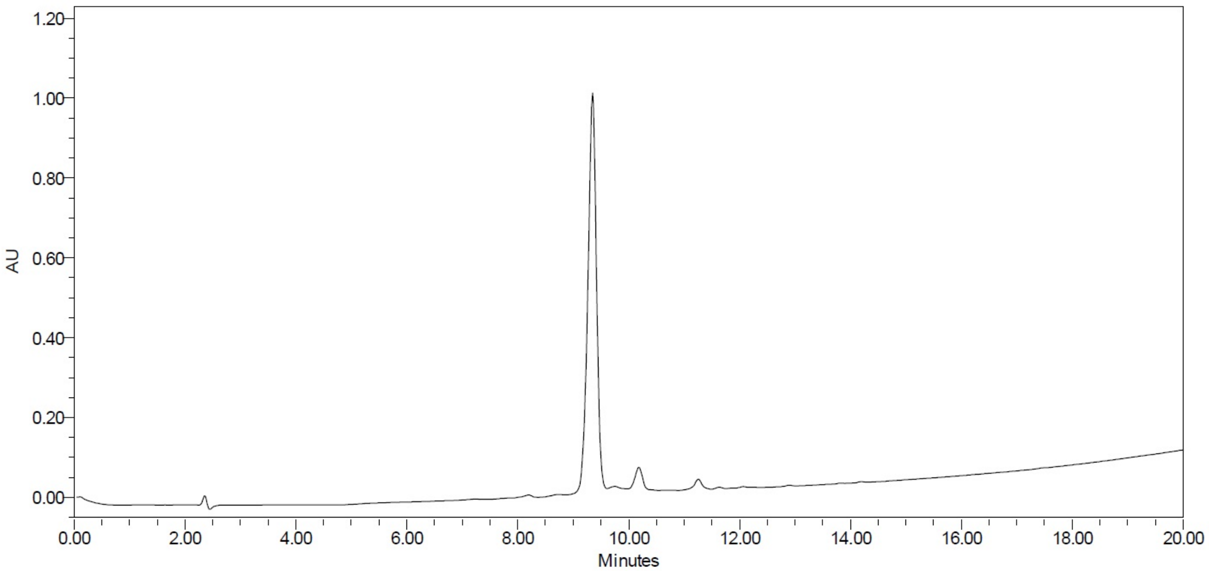

PKKG4 Ac-Ala-Leu-Chg-Ser-Arg-ACC

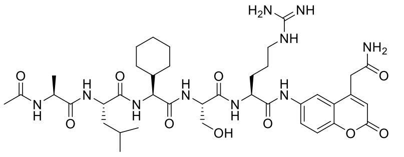

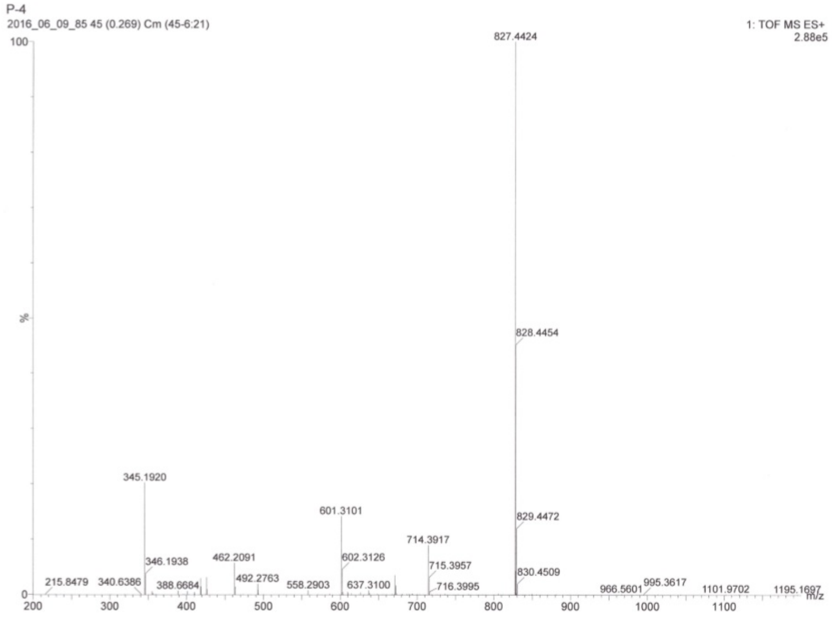

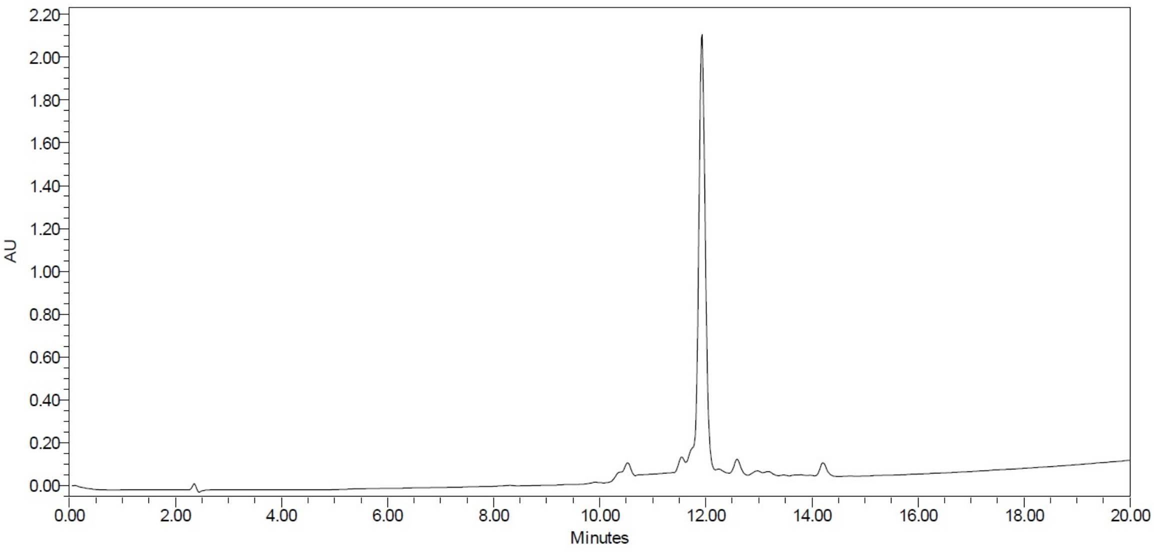

PKKG5 Ac-hCha-Val-Ser-Arg-ACC

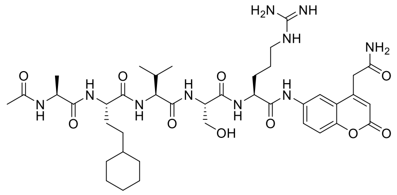

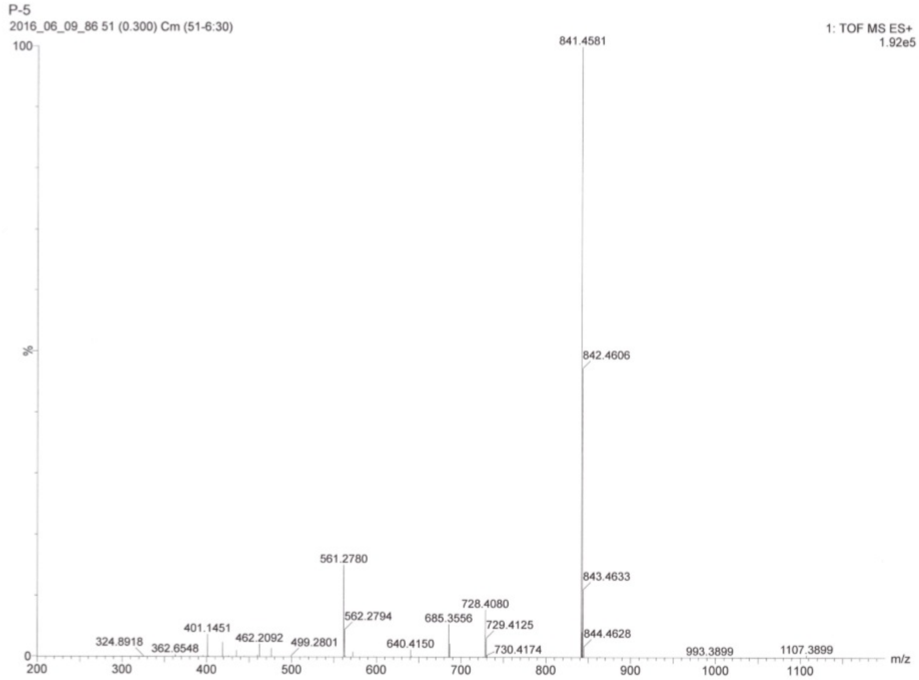

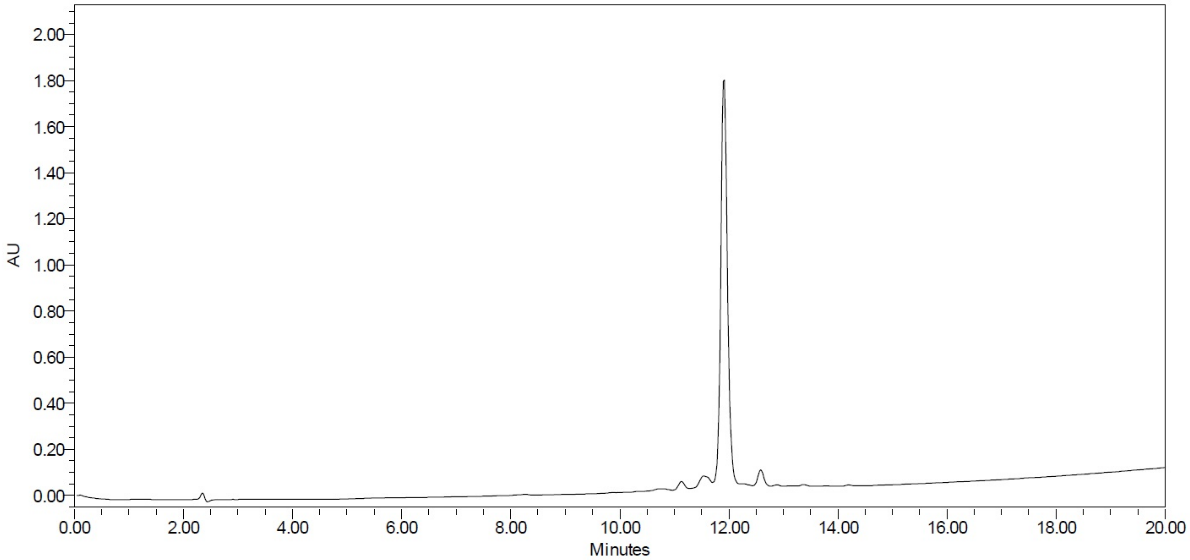

PKKG7 Ac-hLeu-Leu-Val-Ser-Arg-ACC

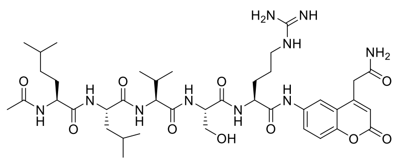

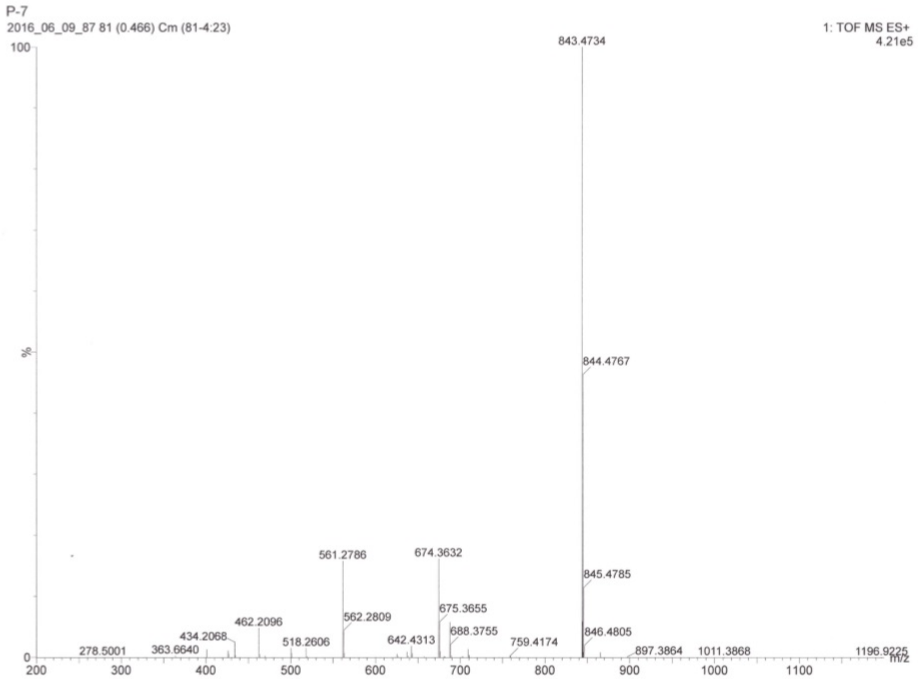

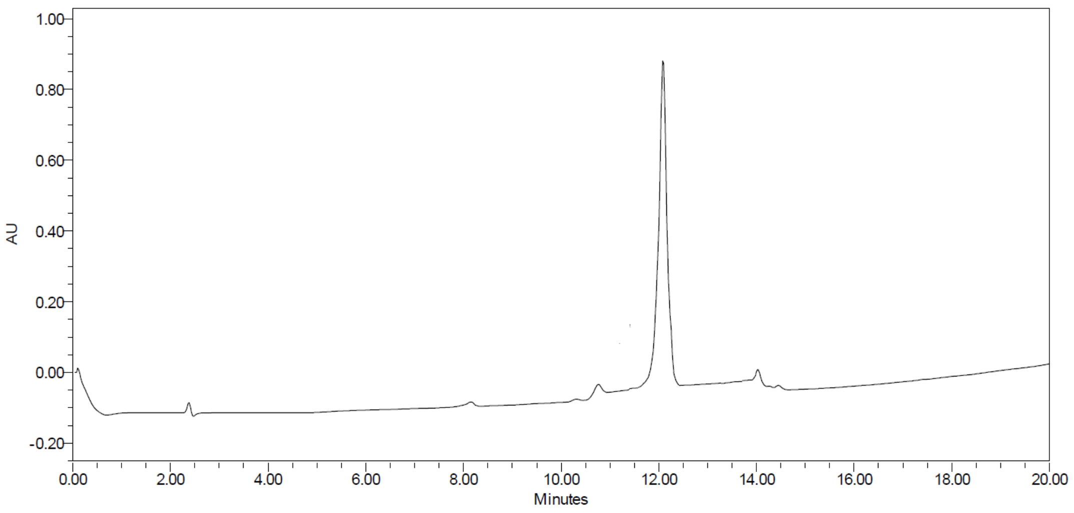

PKKG8 Ac-hTyr(Me)-Leu-Val-Ser-Arg-ACC

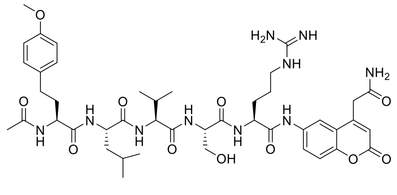

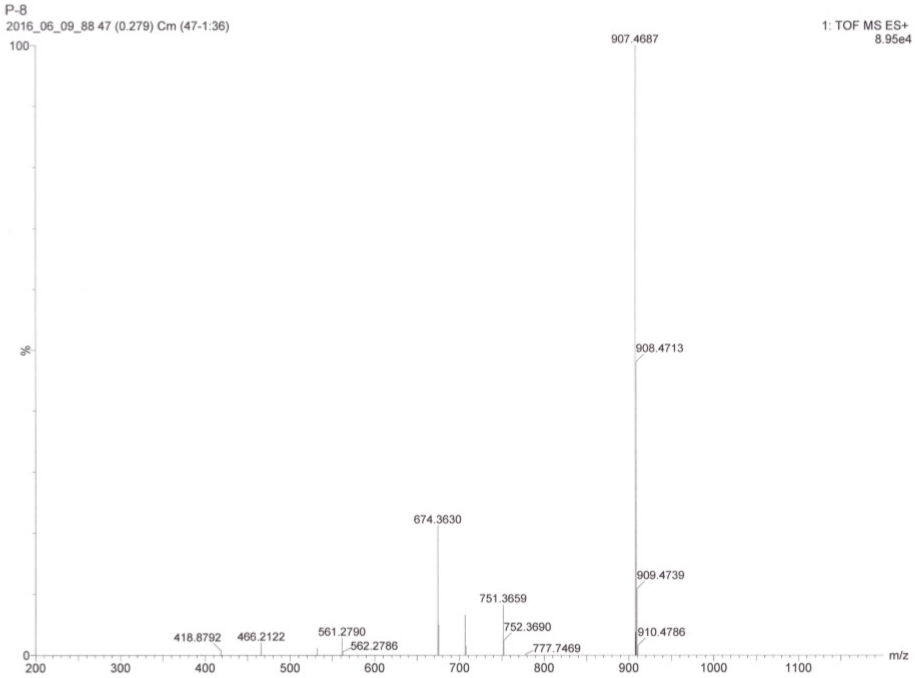

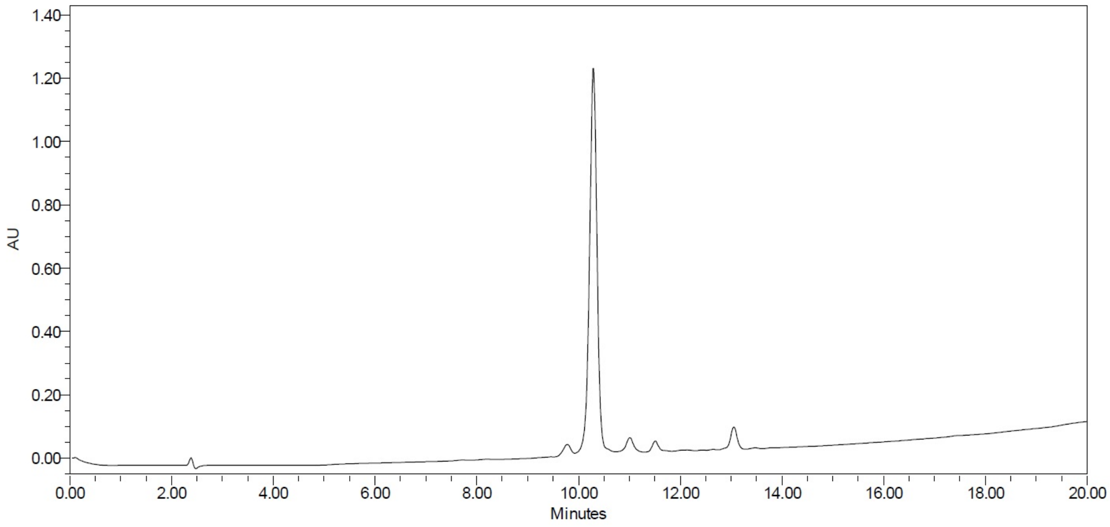

PKKG9 Ac-Ile-Leu-Val-Ser-Arg-ACC

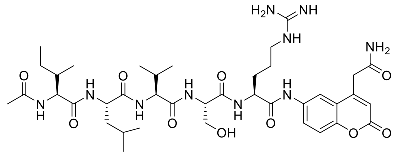

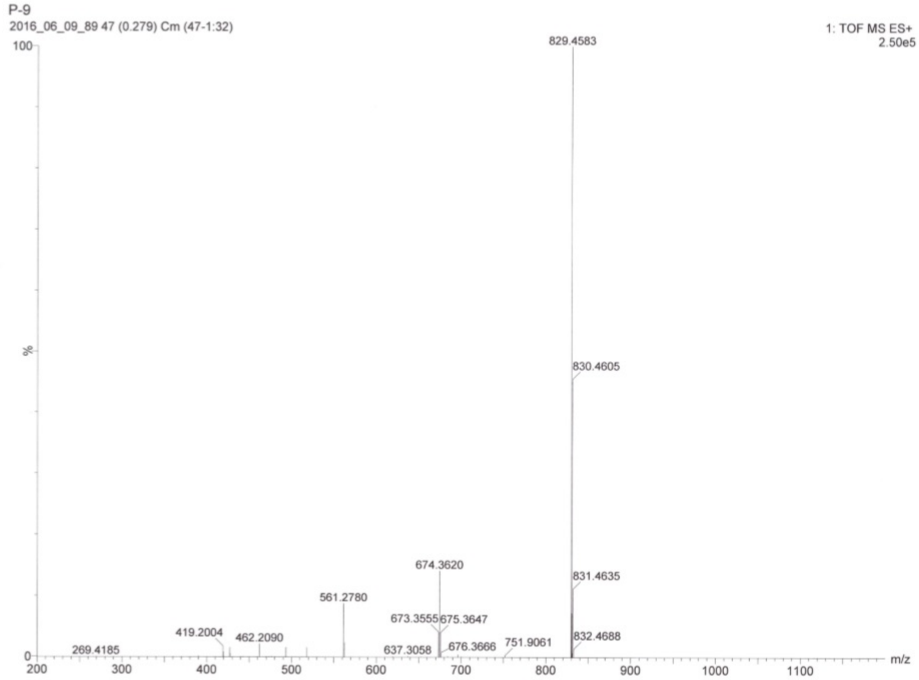

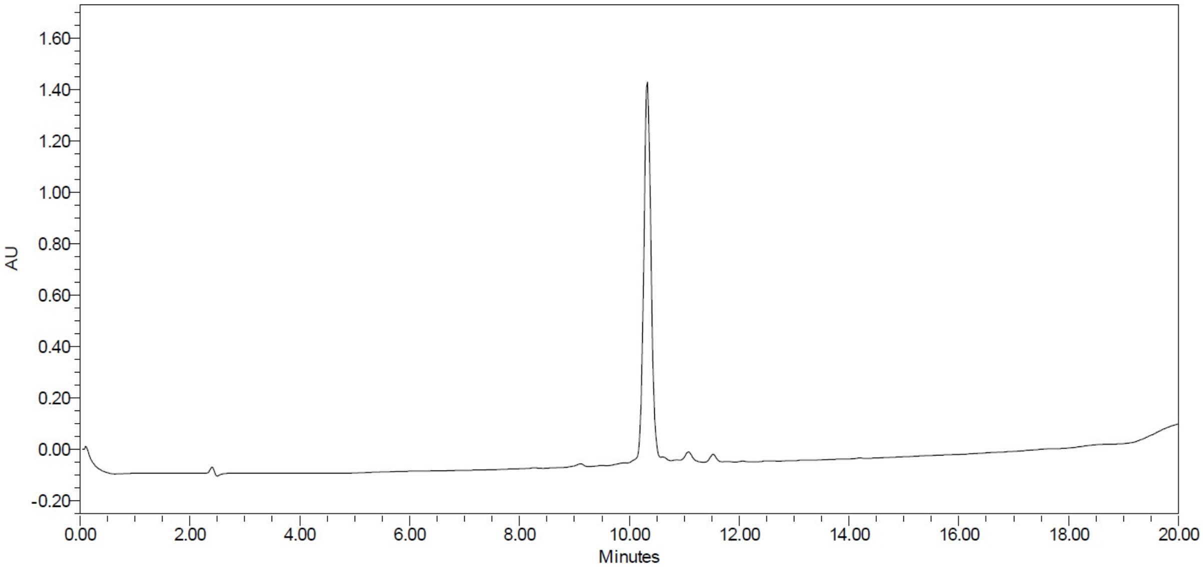

PKKG10 Ac-Nle-Leu-Val-Ser-Arg-ACC

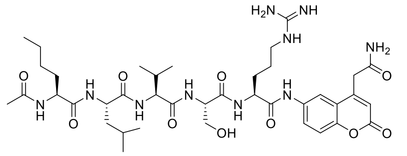

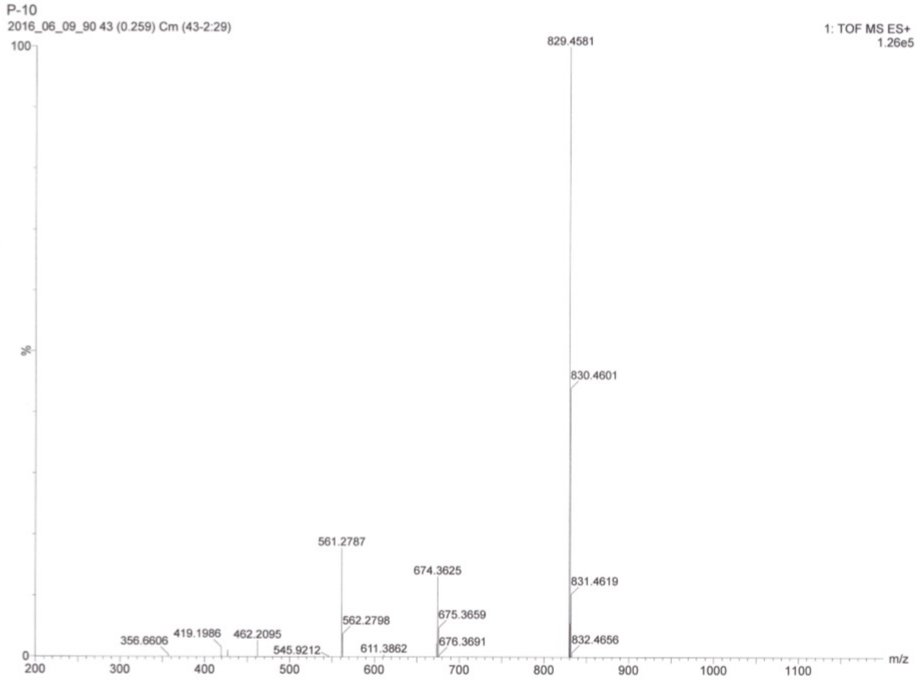

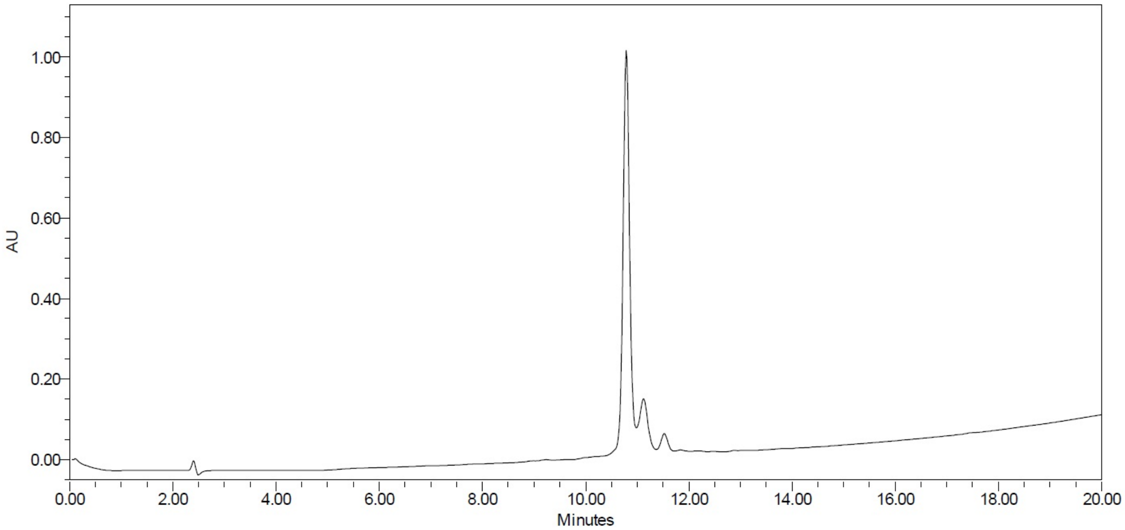

PKKG11 Ac-Tyr(Bzl)-Ser-Val-Ser-Arg-ACC

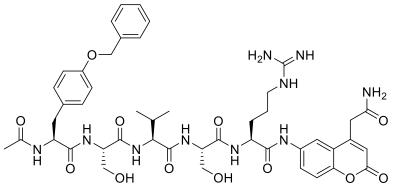

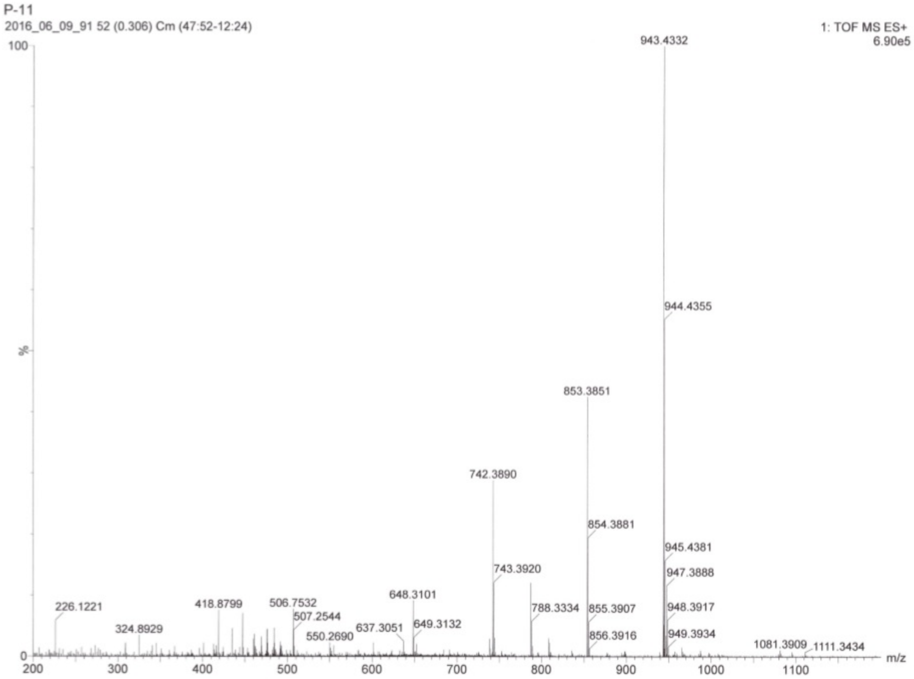

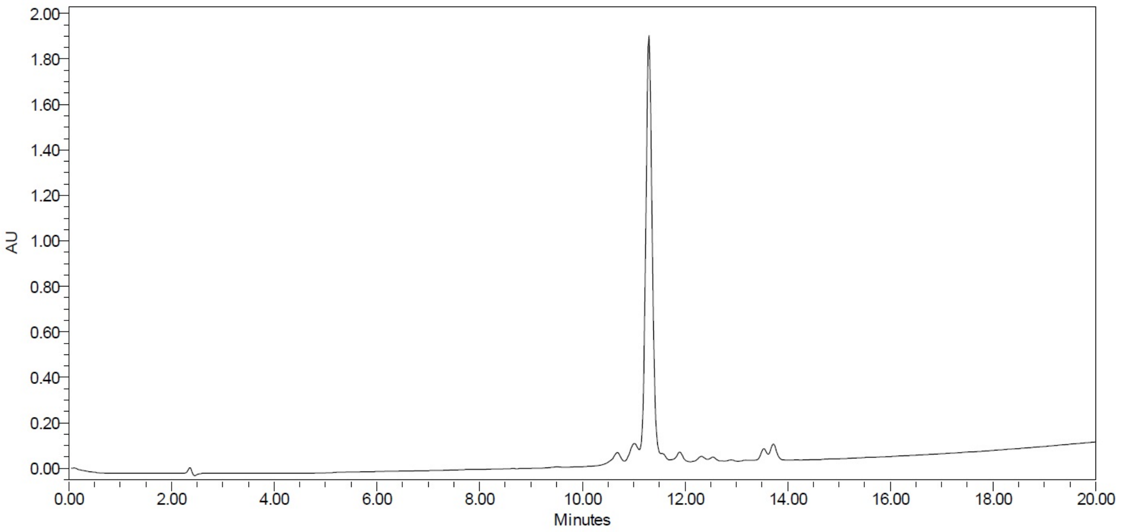

PKKG12 Ac-hTyr(Bzl)-hLeu-Lys(TFA)-Pip-Arg-ACC

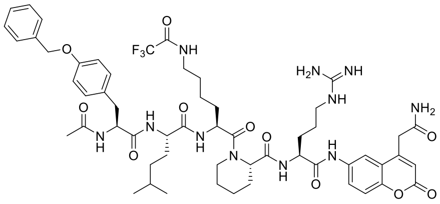

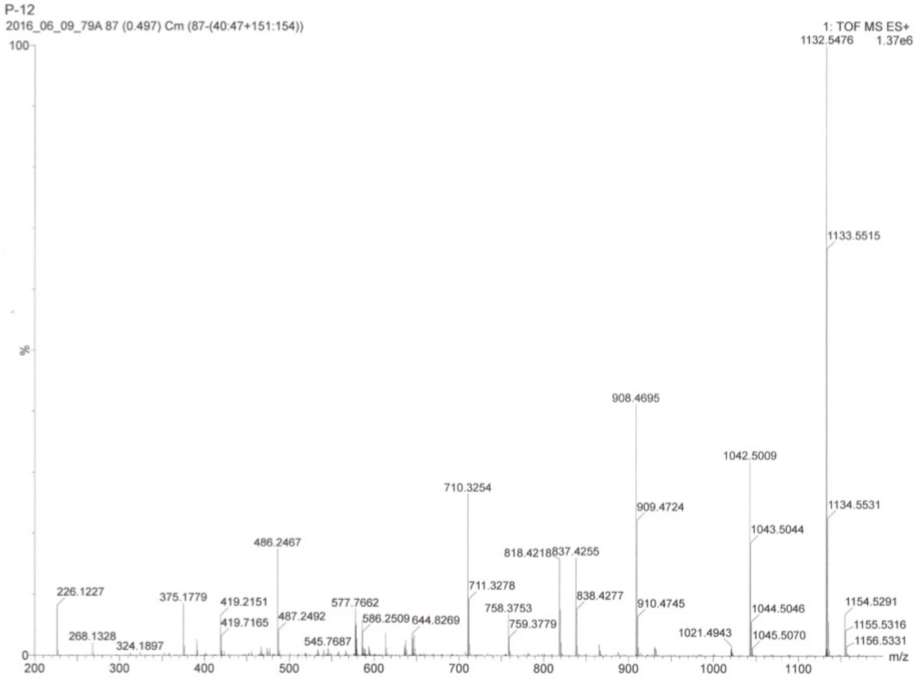

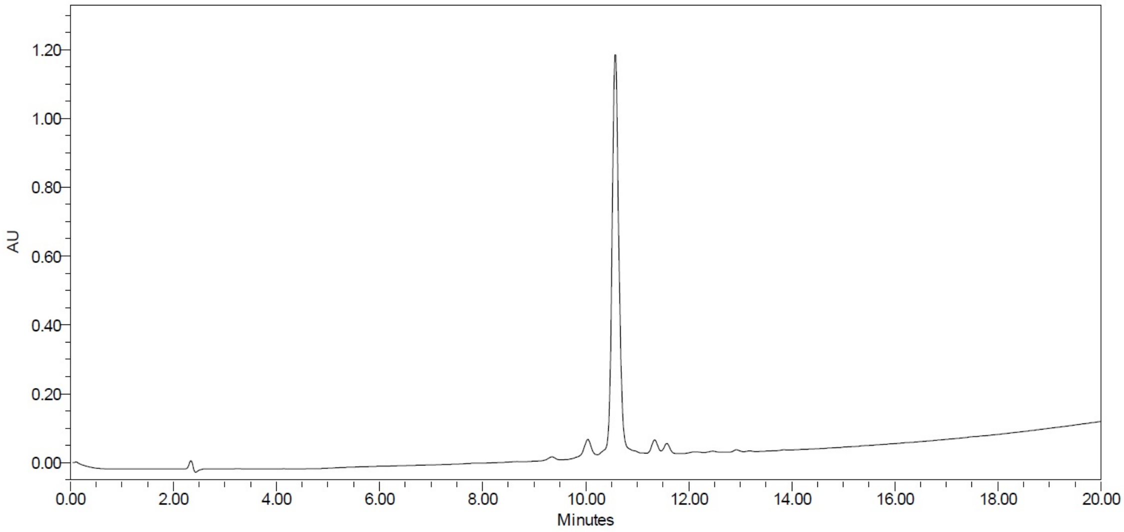

PKKG13 Ac-hTyr(Bzl)-Lys-Val-Ser-Arg-ACC

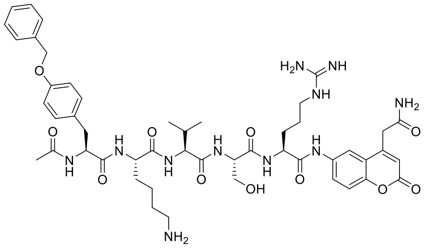

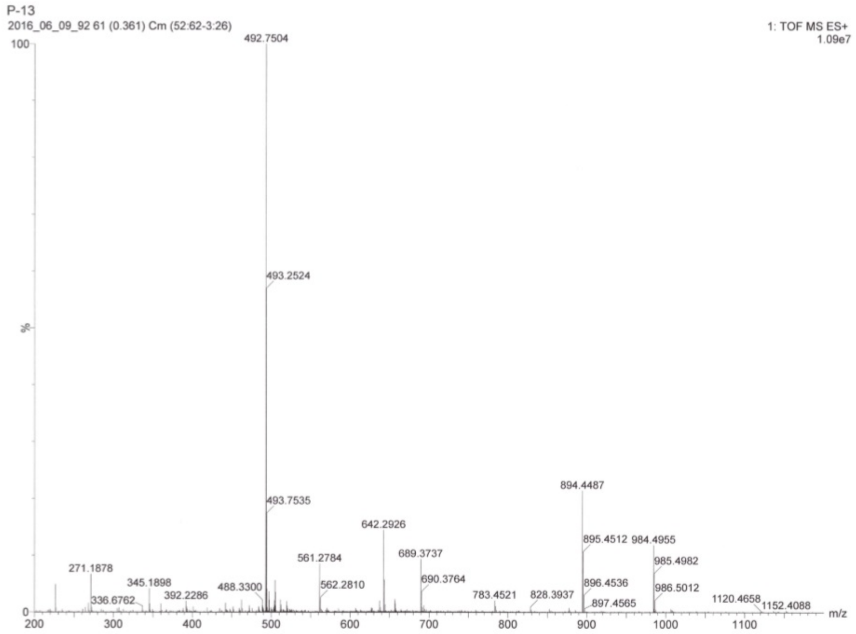

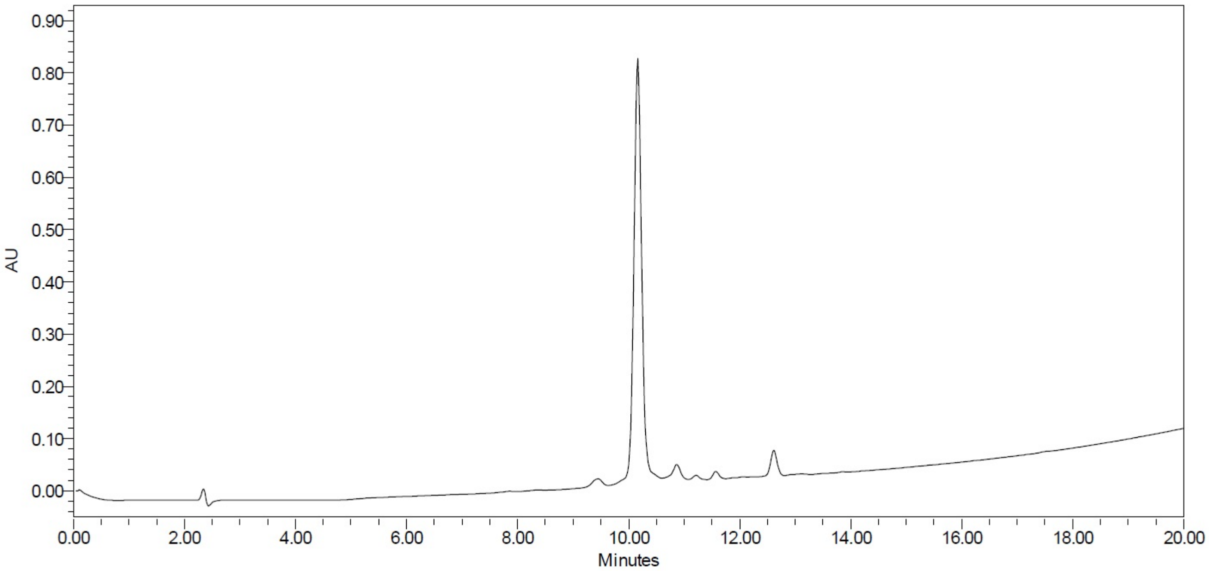

PKKG14 Ac-hTyr(Bzl)-Thr-Val-Ser-Arg-ACC

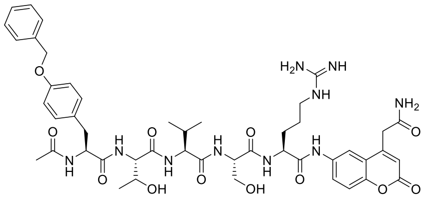

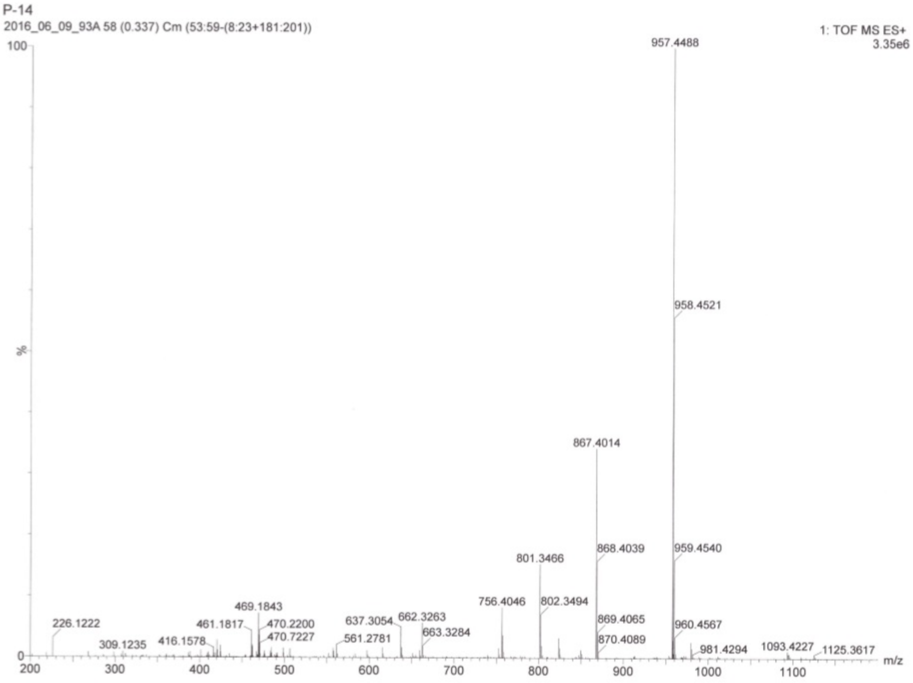

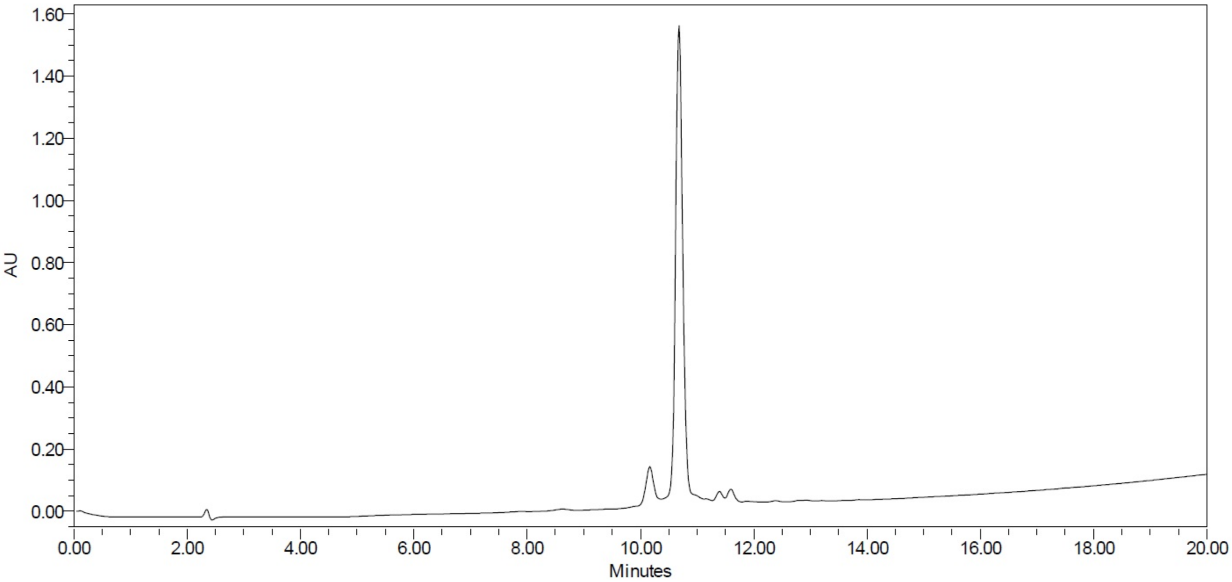

PKKG15 Ac-hTyr(Bzl)-Asp-Lys(TFA)-Pro-Arg-ACC

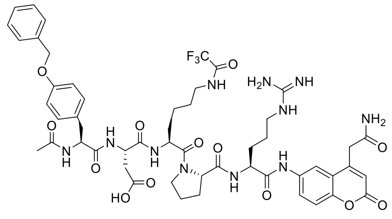

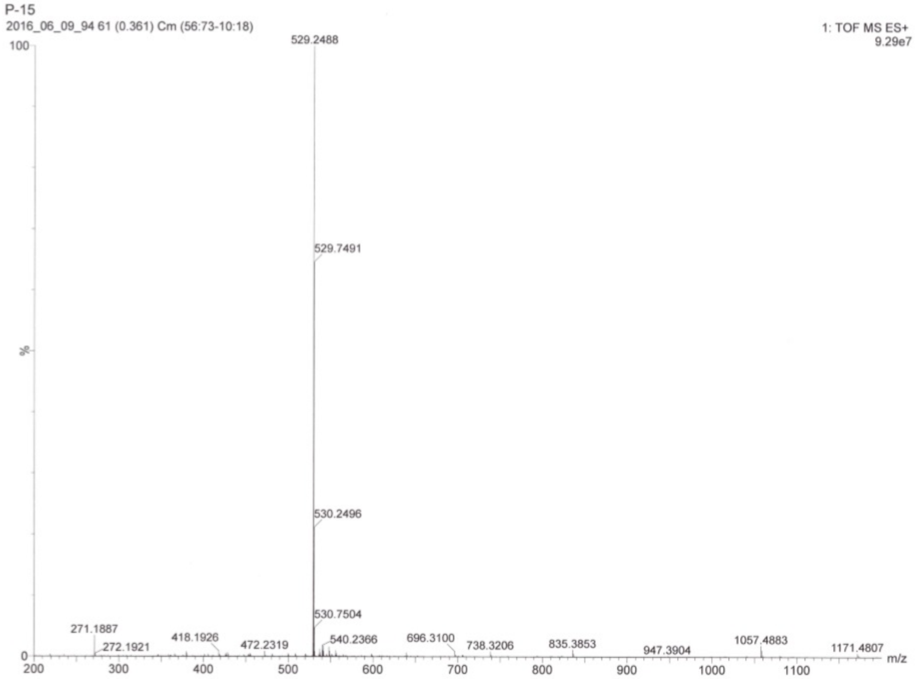

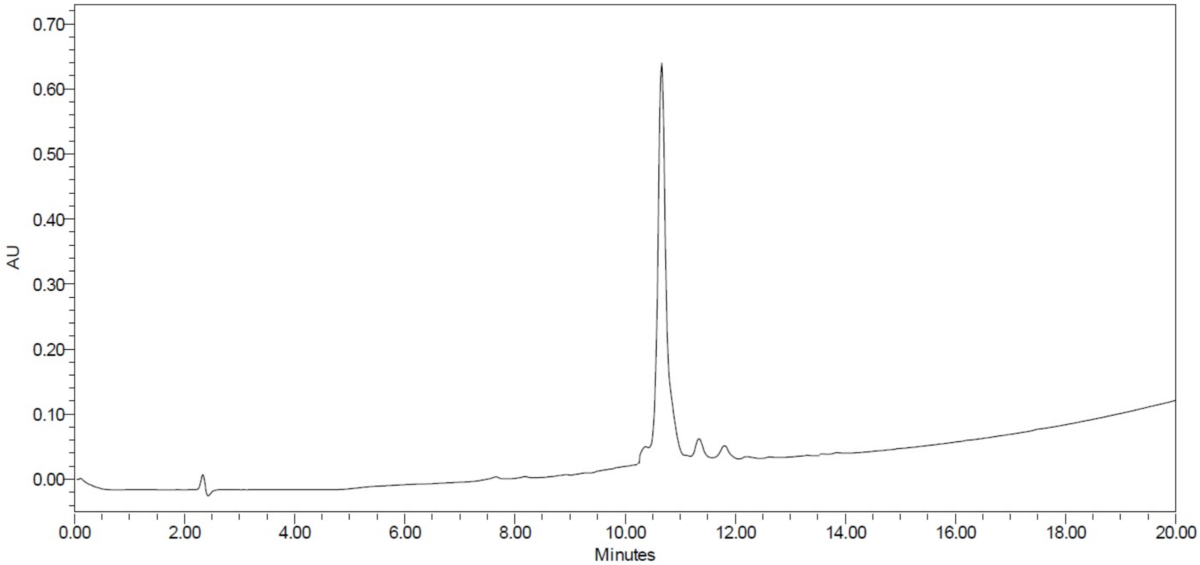

PKKG16 Ac-Phe(guan)-Leu-Val-Pro-Arg-ACC

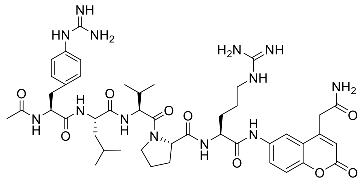

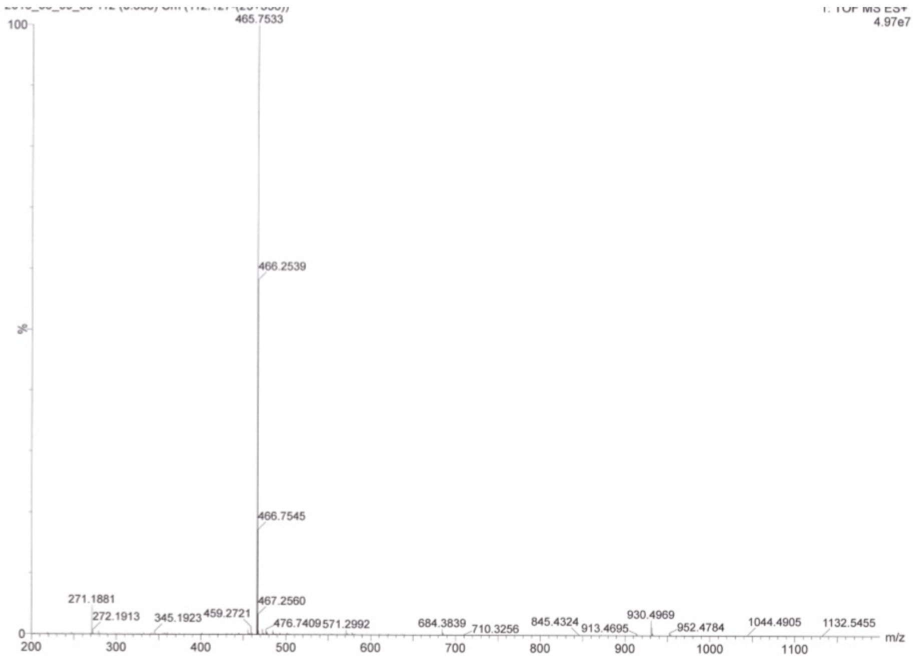

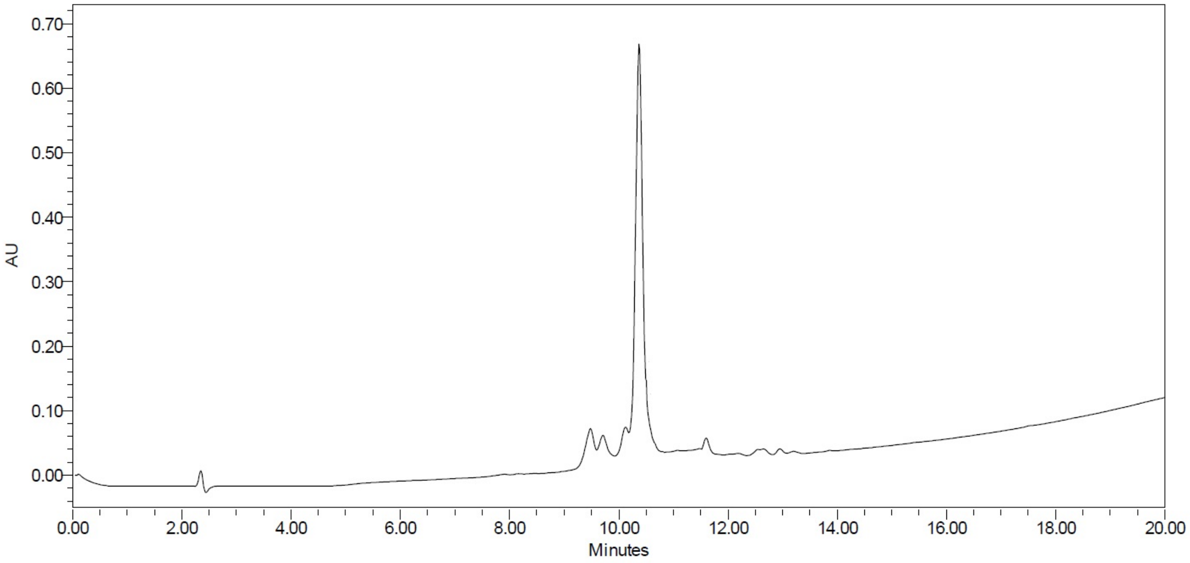

PKKG17 Ac-hTyr(Bzl)-Leu-Val-Pro-Arg-ACC

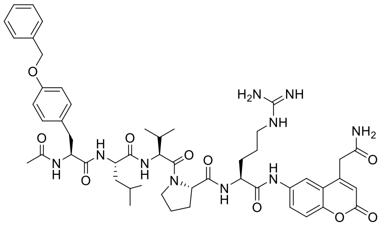

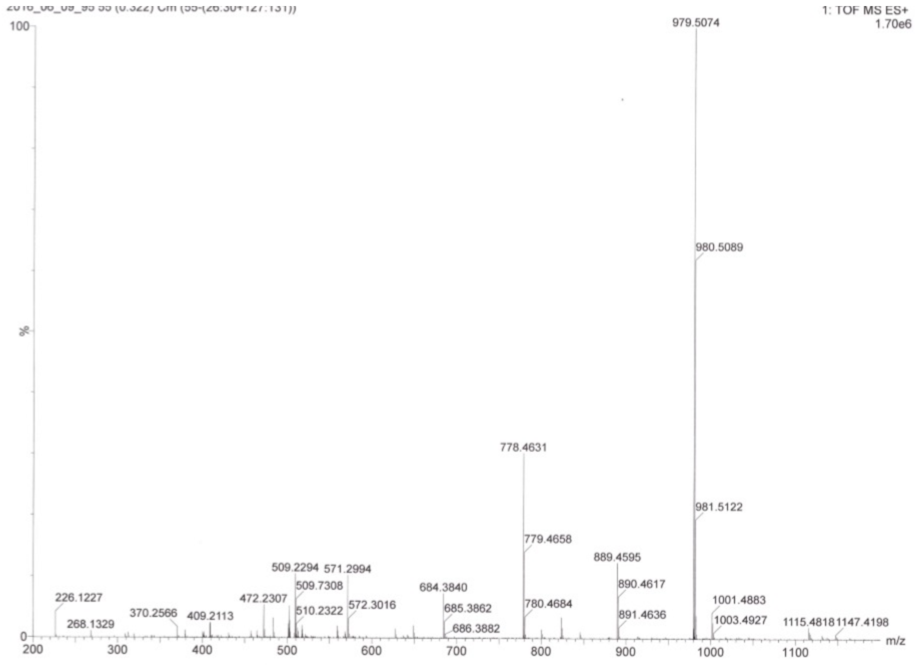

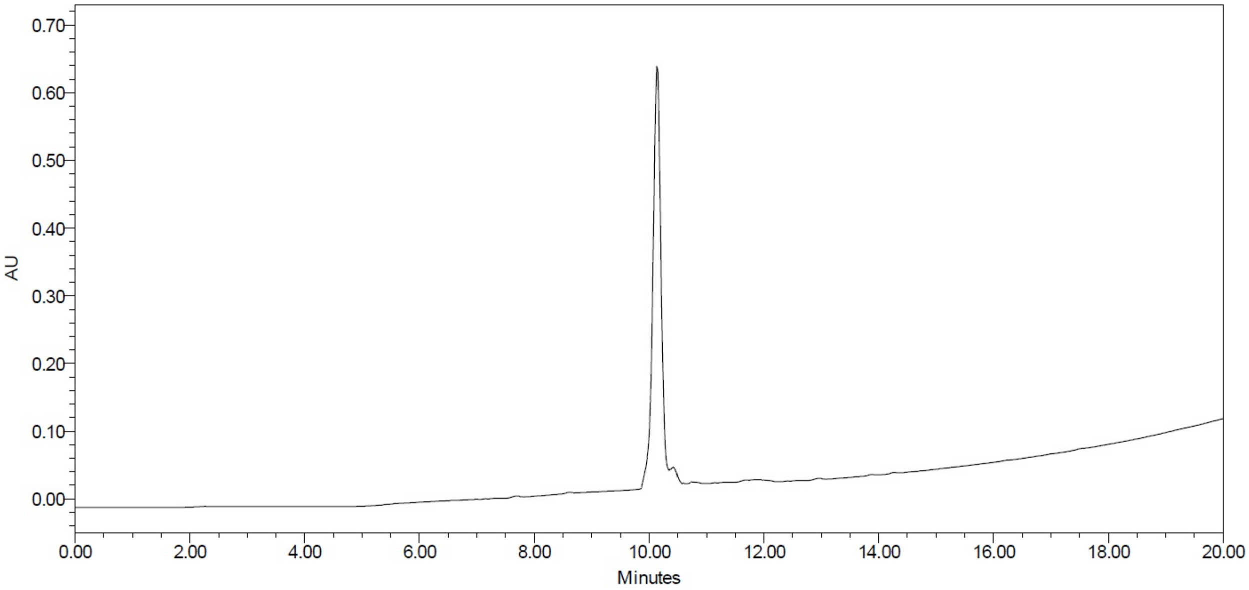

ACC-Ahx-Ala-Leu-Val-Ser-Arg-Gly-Ala-Lys(Dnp)-Gly

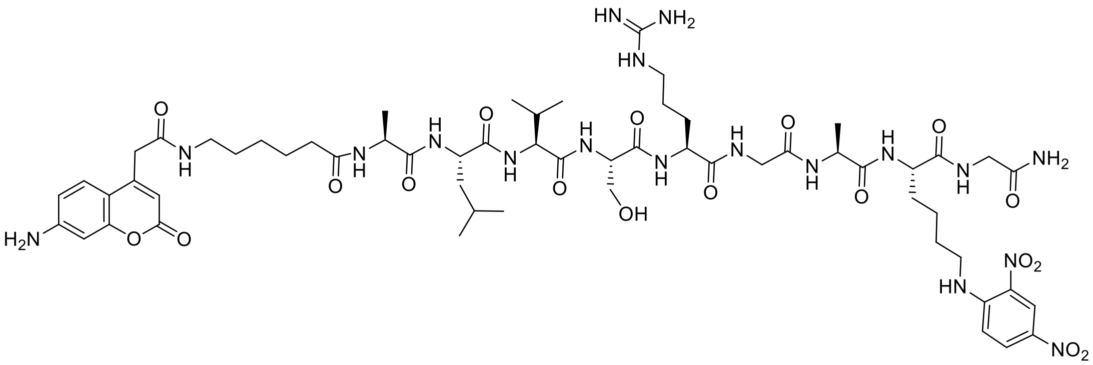

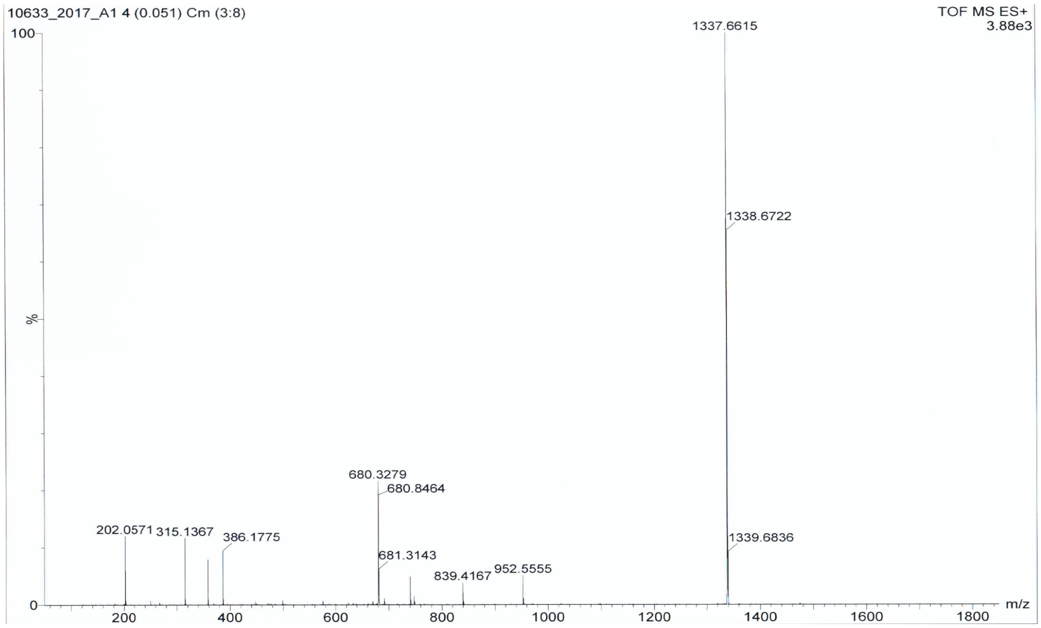

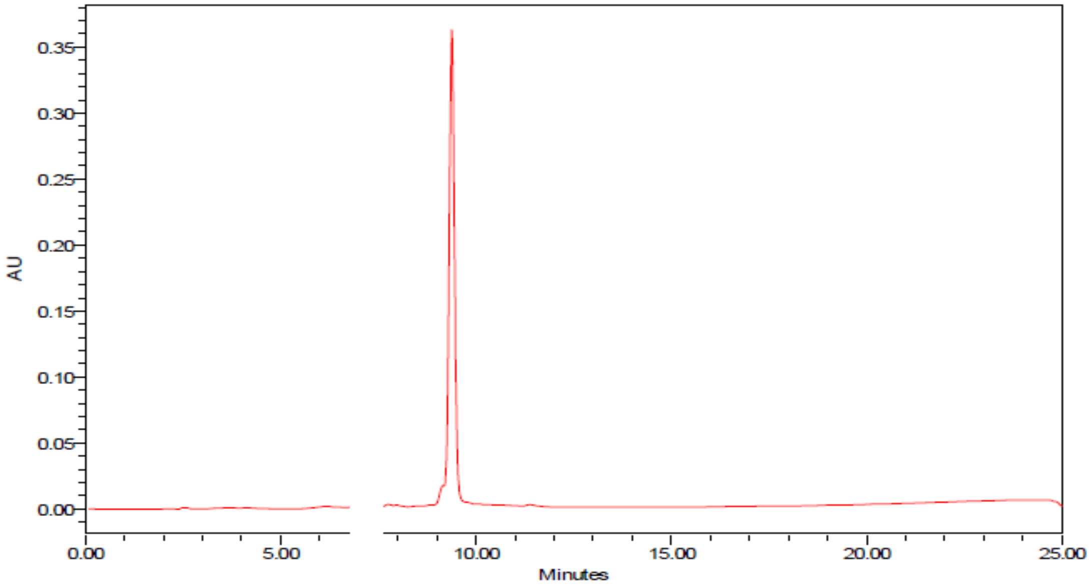

ACC-Ahx-Ala-Leu-Val-Ser-Arg-Gly-Arg-Lys(Dnp)-Gly

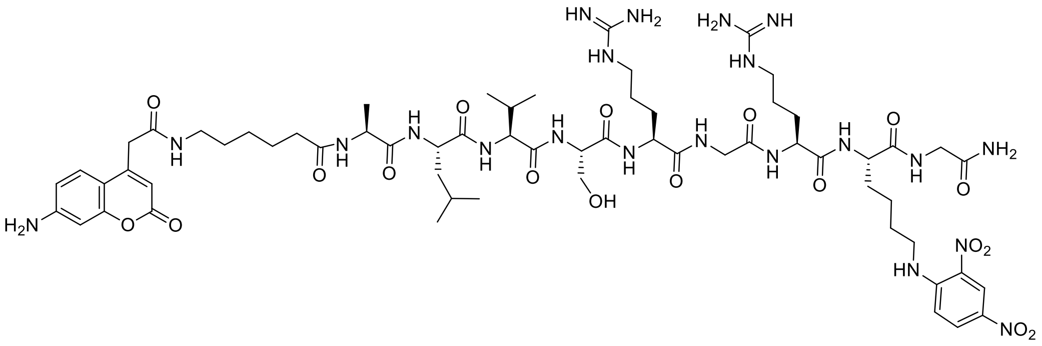

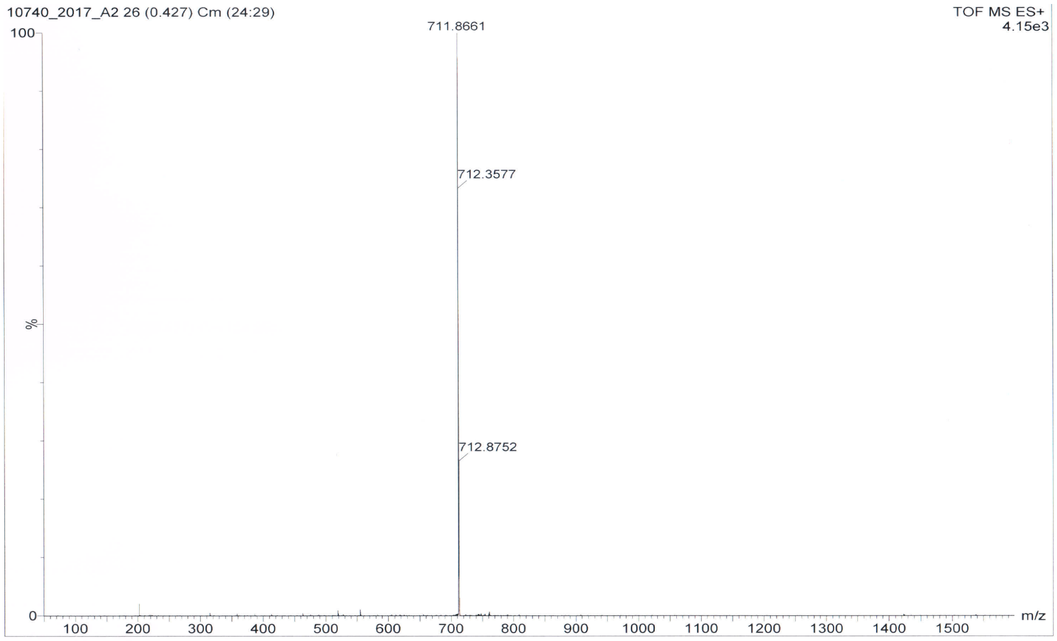

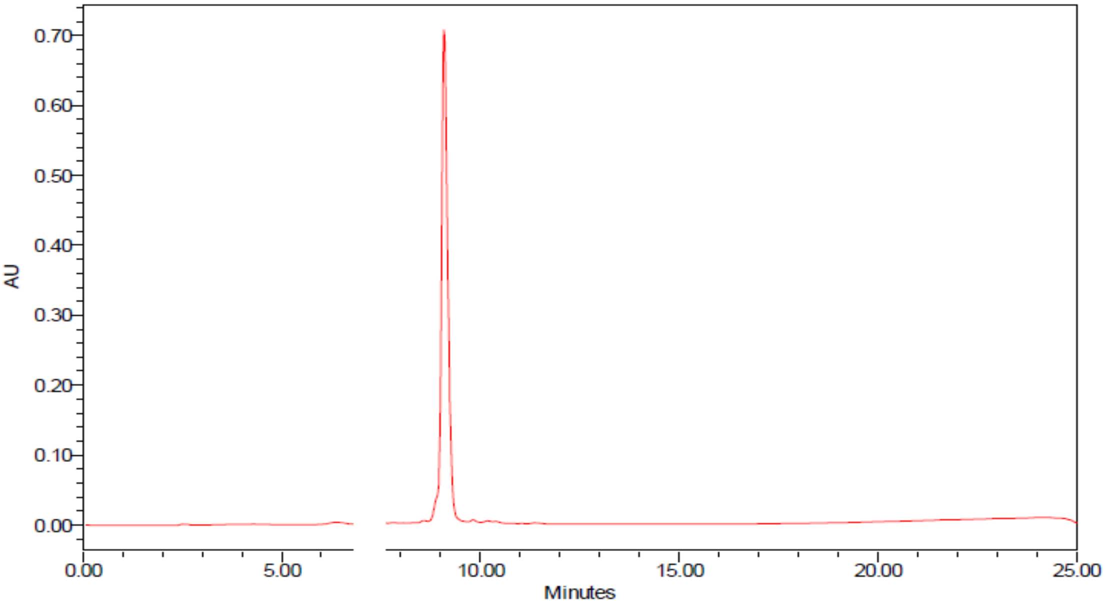

ACC-Ahx-Ala-Leu-Val-Ser-Arg-Gly-Asn-Lys(Dnp)-Gly

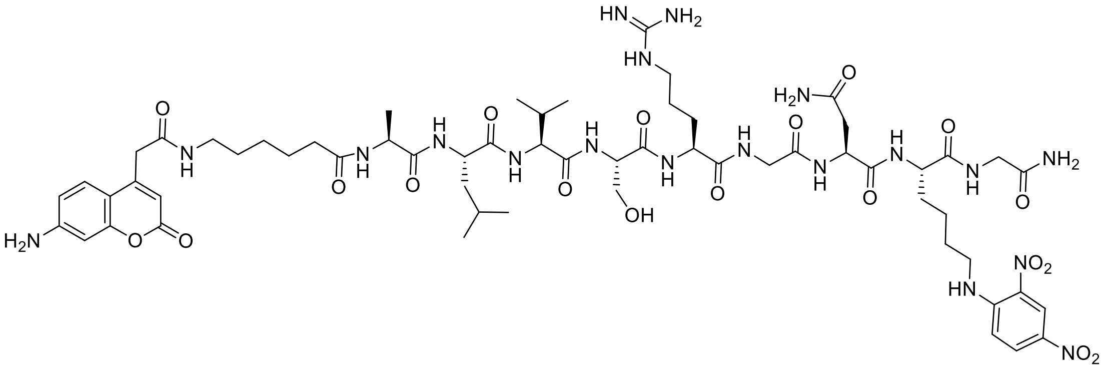

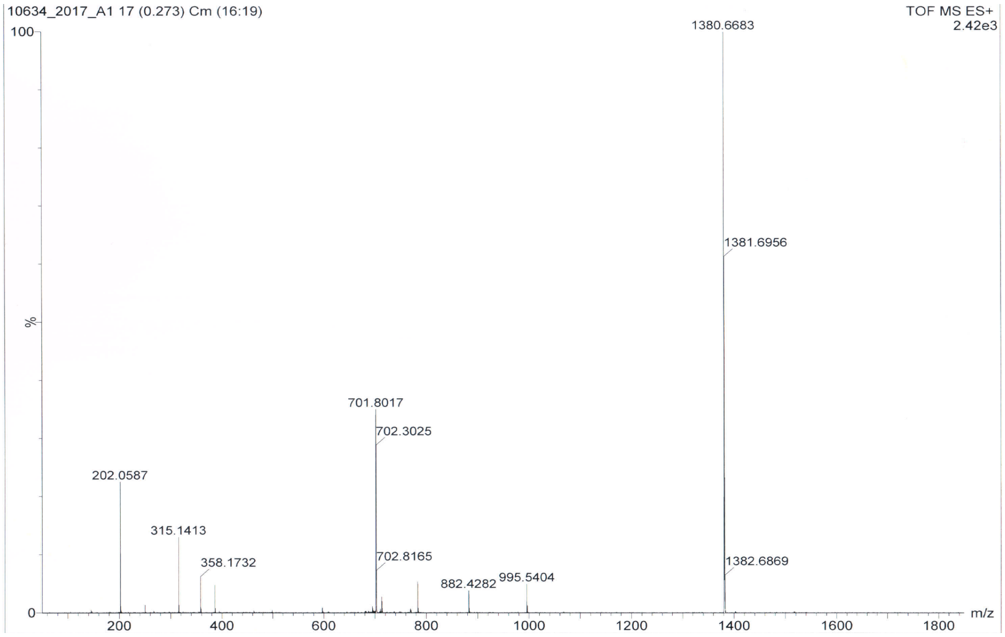

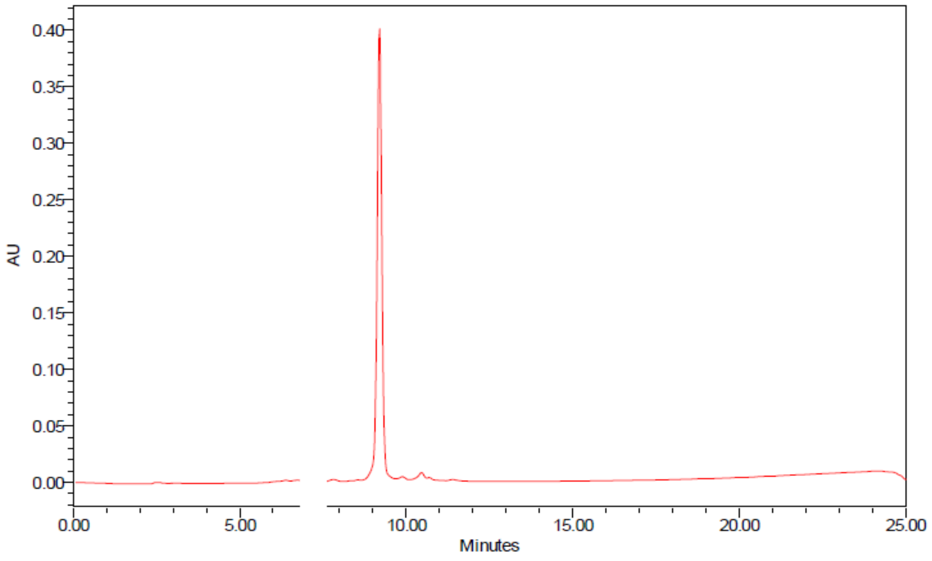

ACC-Ahx-Ala-Leu-Val-Ser-Arg-Gly-Asp-Lys(Dnp)-Gly

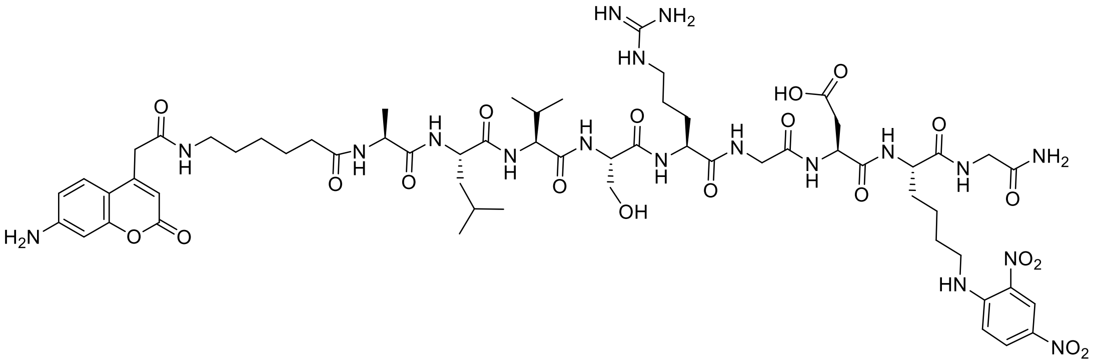

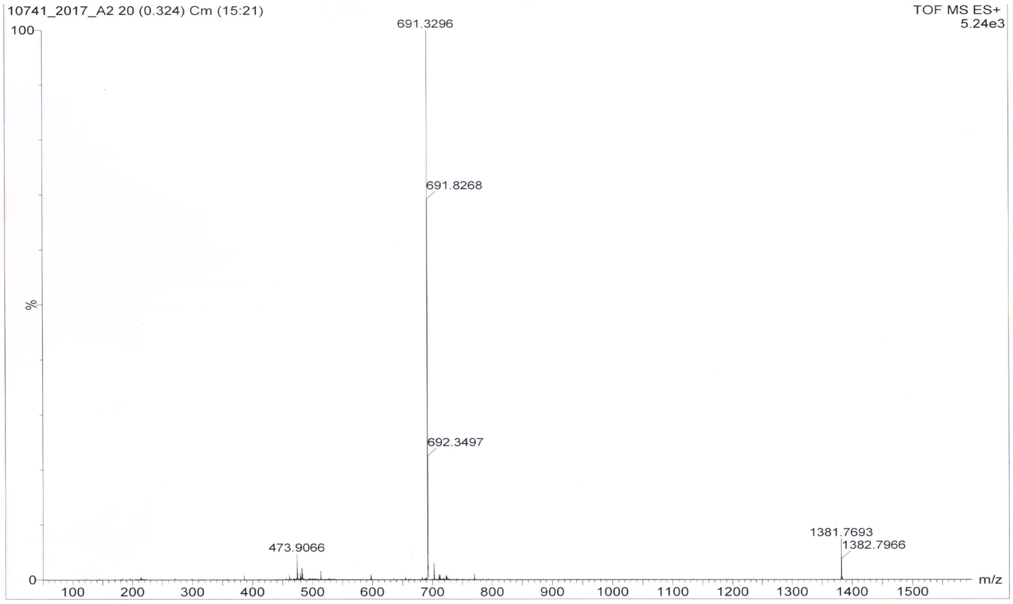

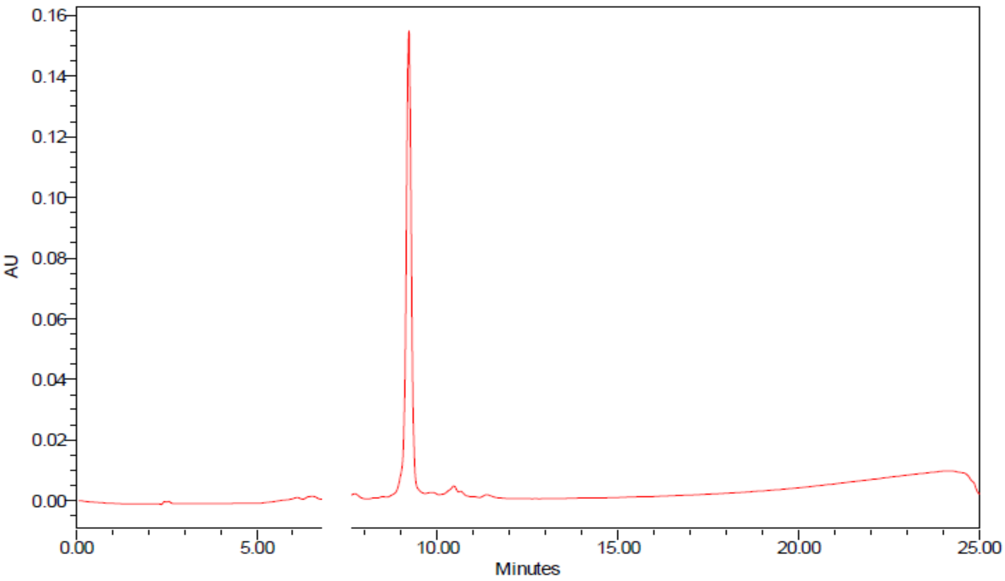

ACC-Ahx-Ala-Leu-Val-Ser-Arg-Gly-Glu-Lys(Dnp)-Gly

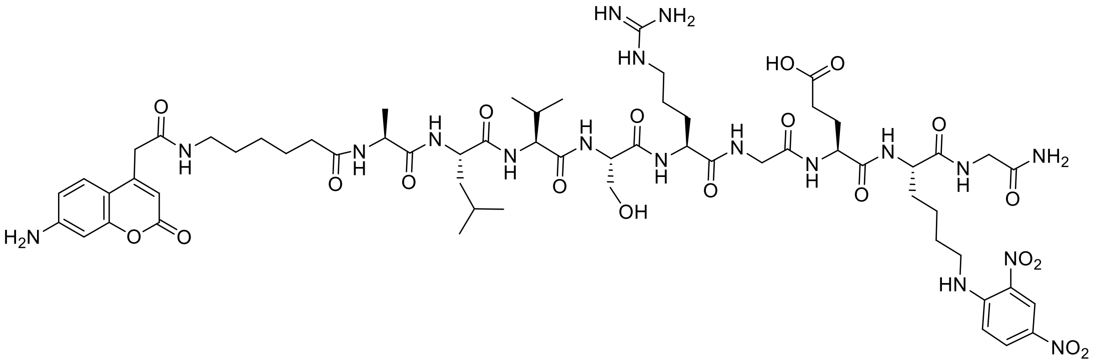

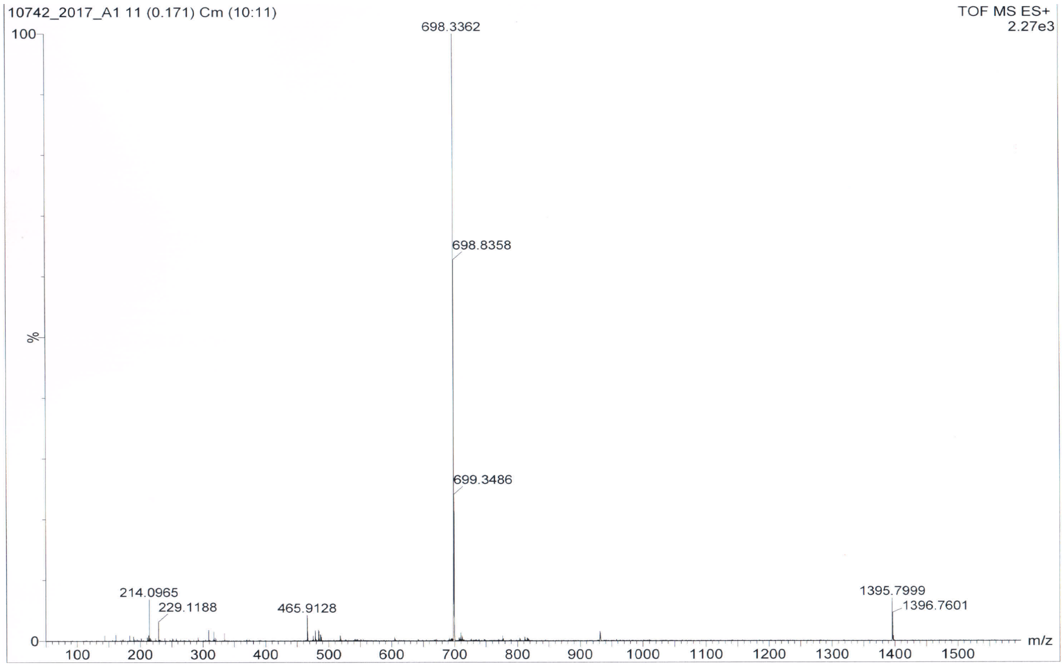

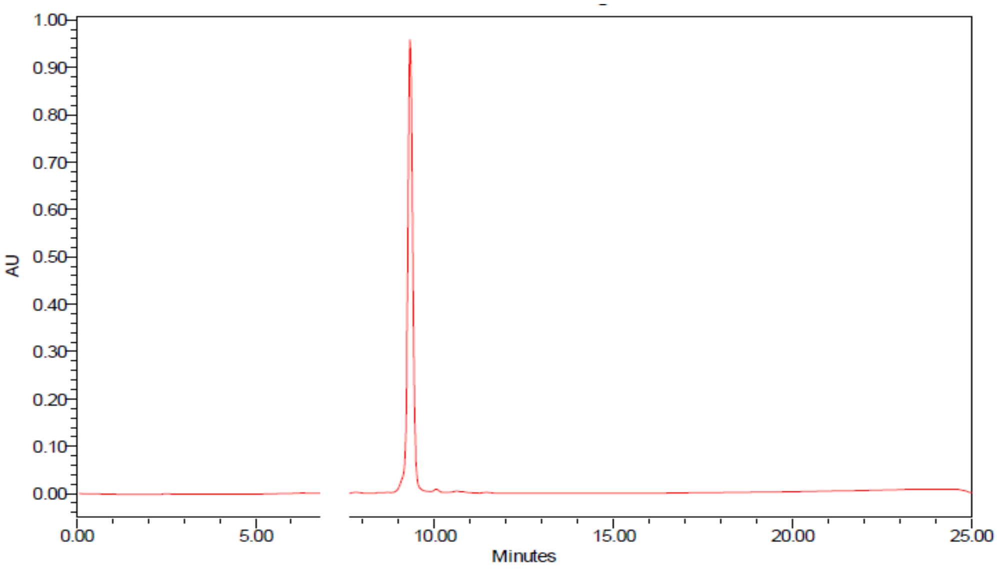

ACC-Ahx-Ala-Leu-Val-Ser-Arg-Gly-Gln-Lys(Dnp)-Gly

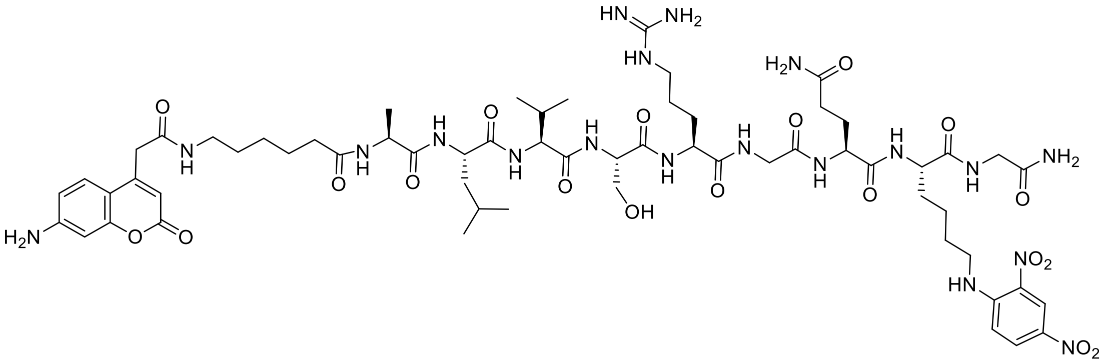

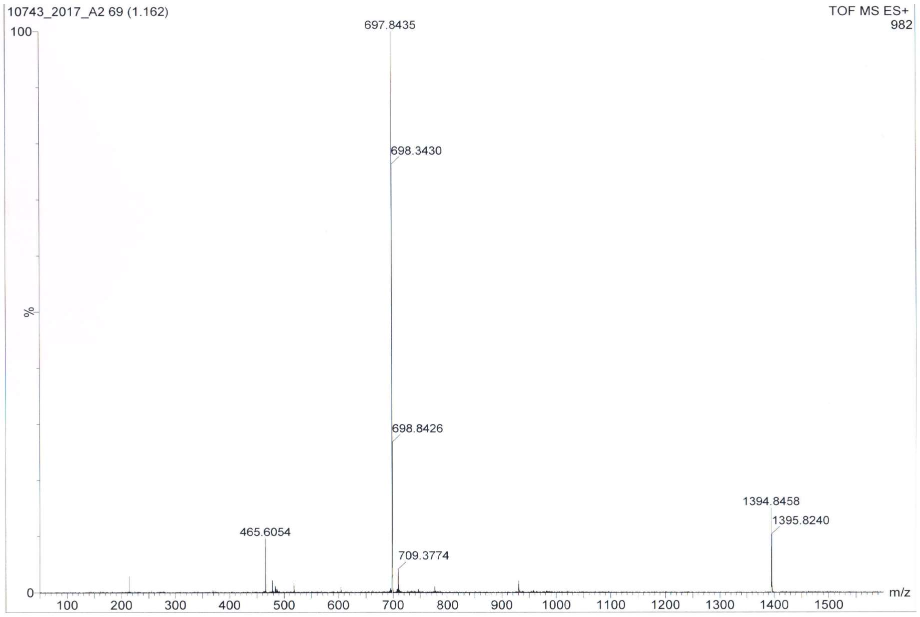

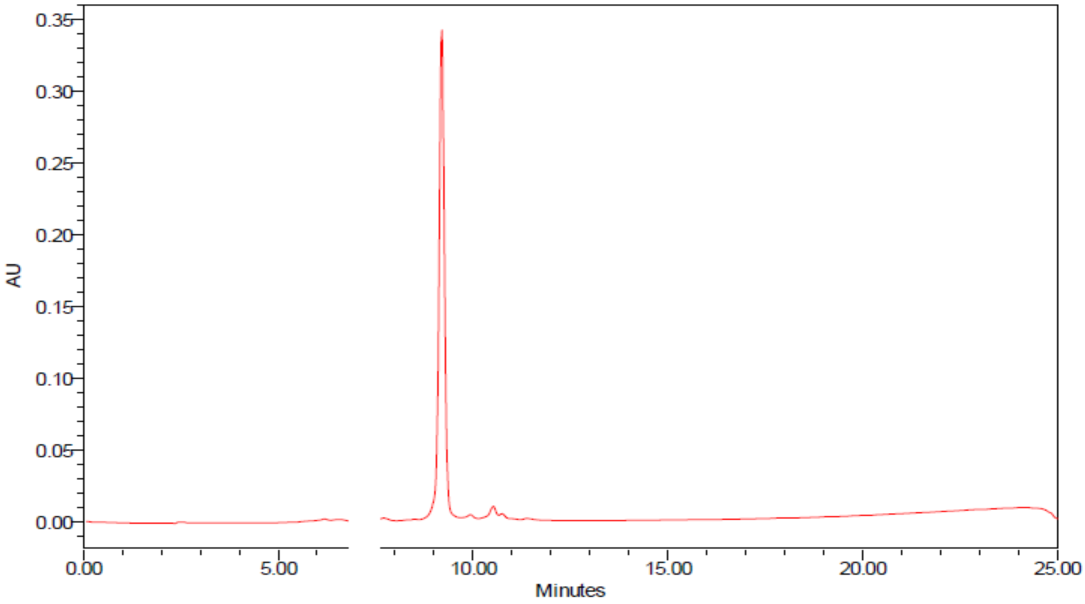

ACC-Ahx-Ala-Leu-Val-Ser-Arg-Gly-Gly-Lys(Dnp)-Gly

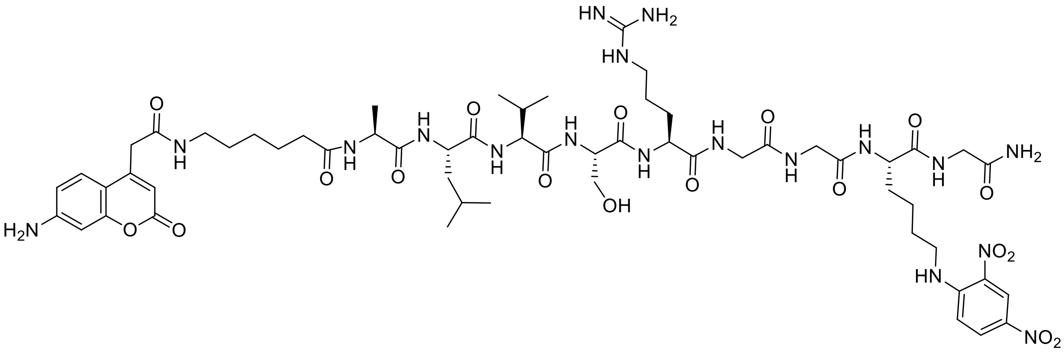

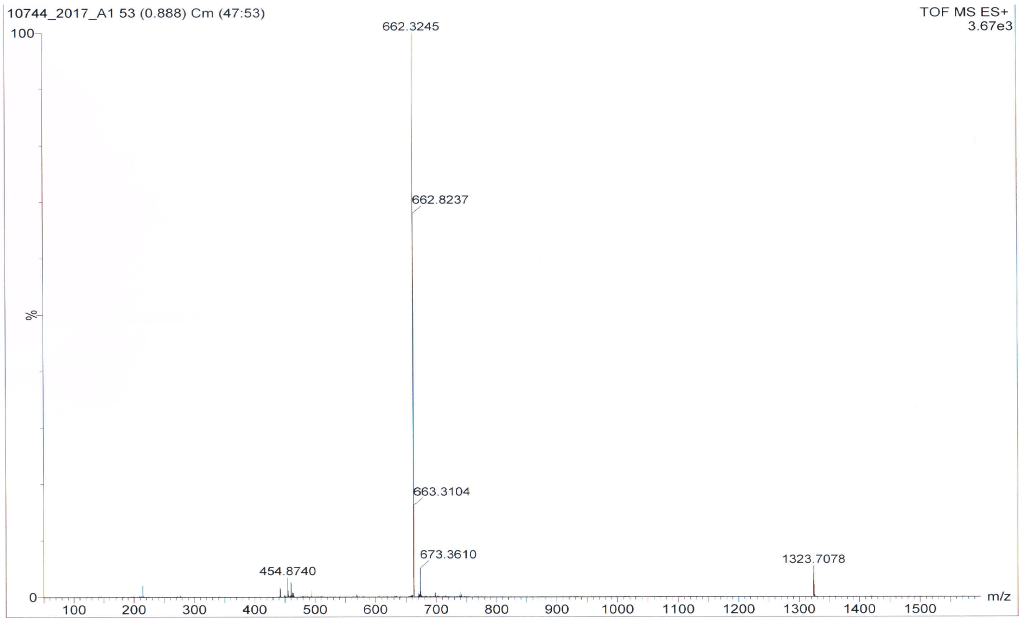

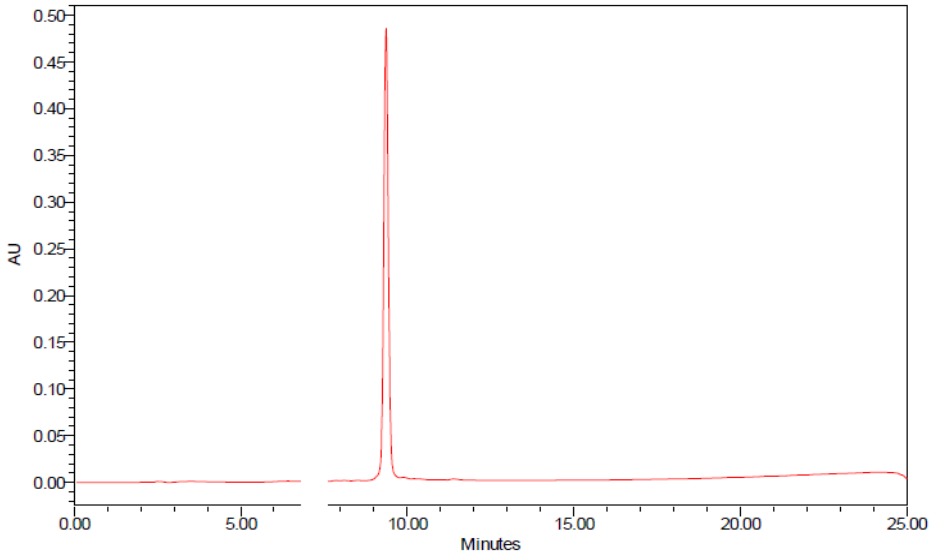

ACC-Ahx-Ala-Leu-Val-Ser-Arg-Gly-His-Lys(Dnp)-Gly

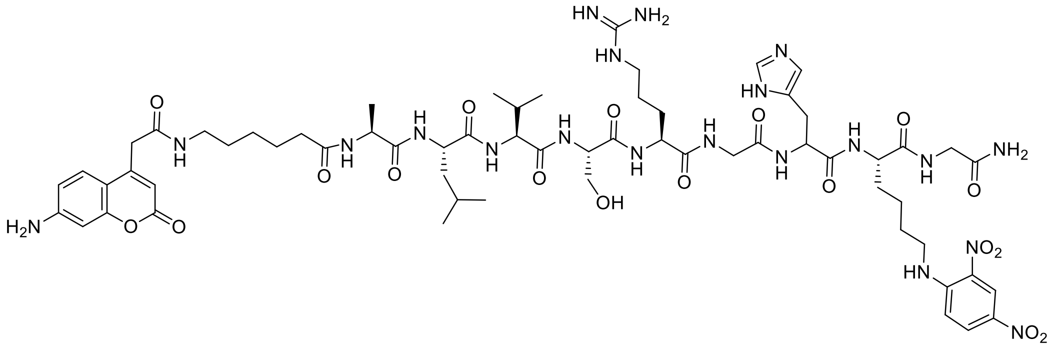

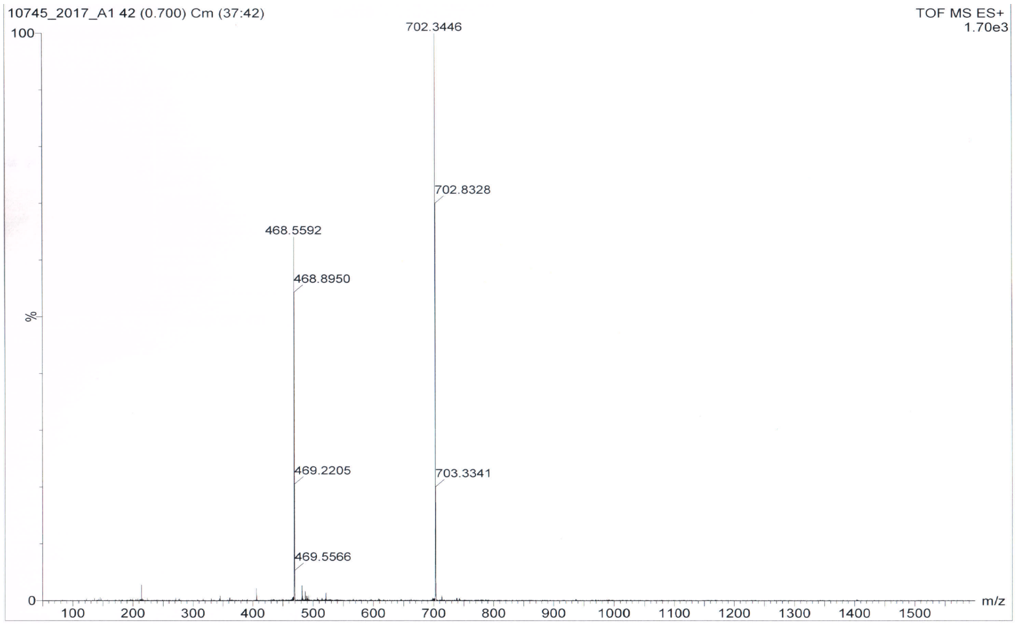

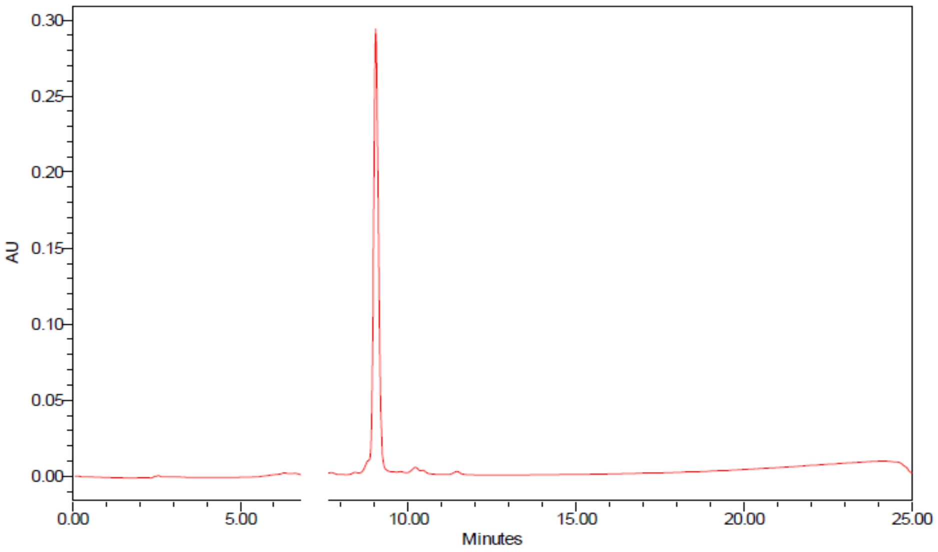

ACC-Ahx-Ala-Leu-Val-Ser-Arg-Gly-Ile-Lys(Dnp)-Gly

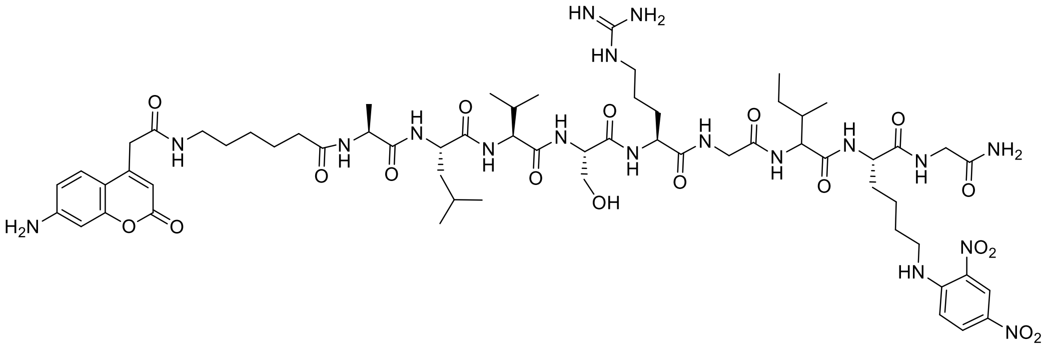

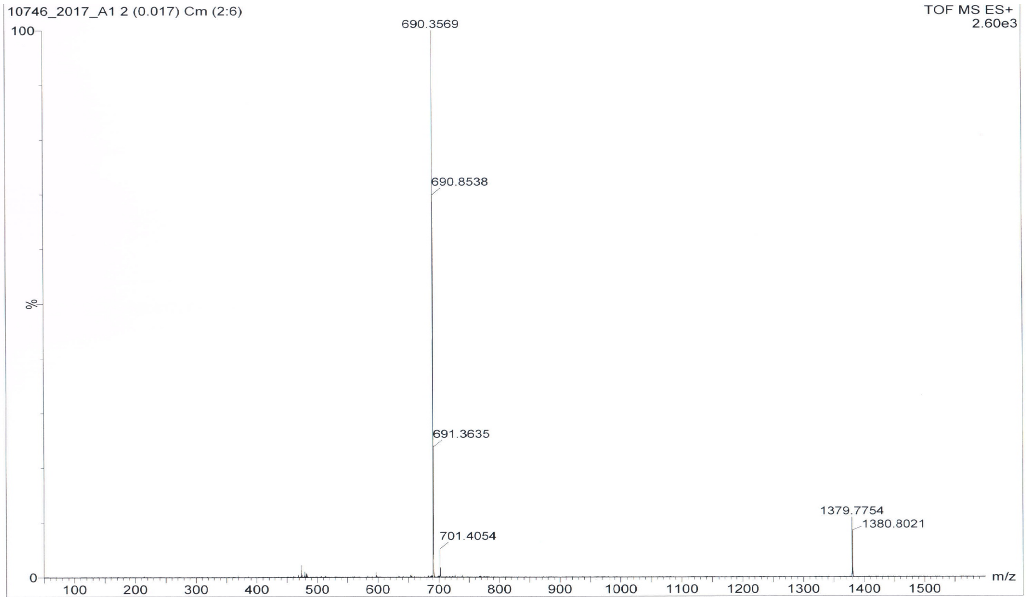

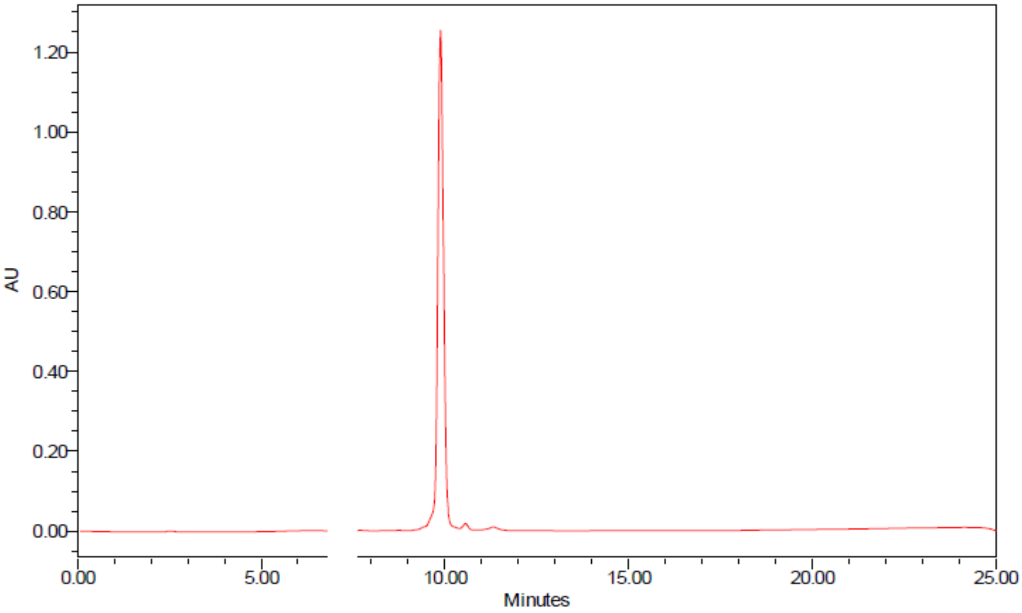

ACC-Ahx-Ala-Leu-Val-Ser-Arg-Gly-Leu-Lys(Dnp)-Gly

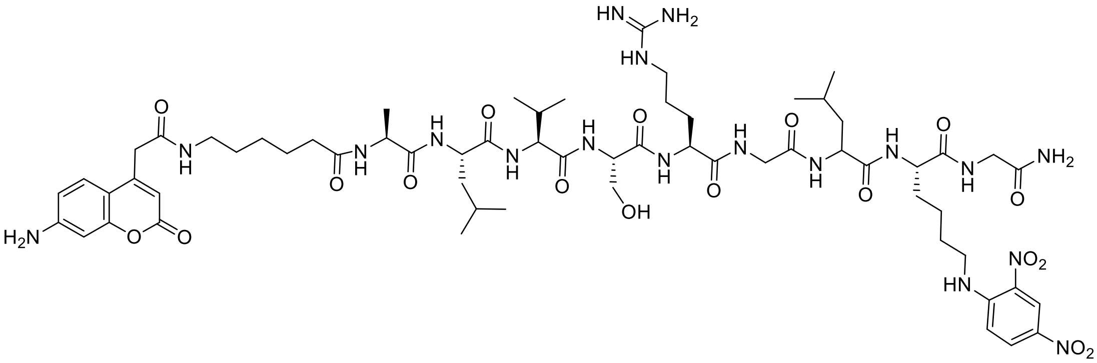

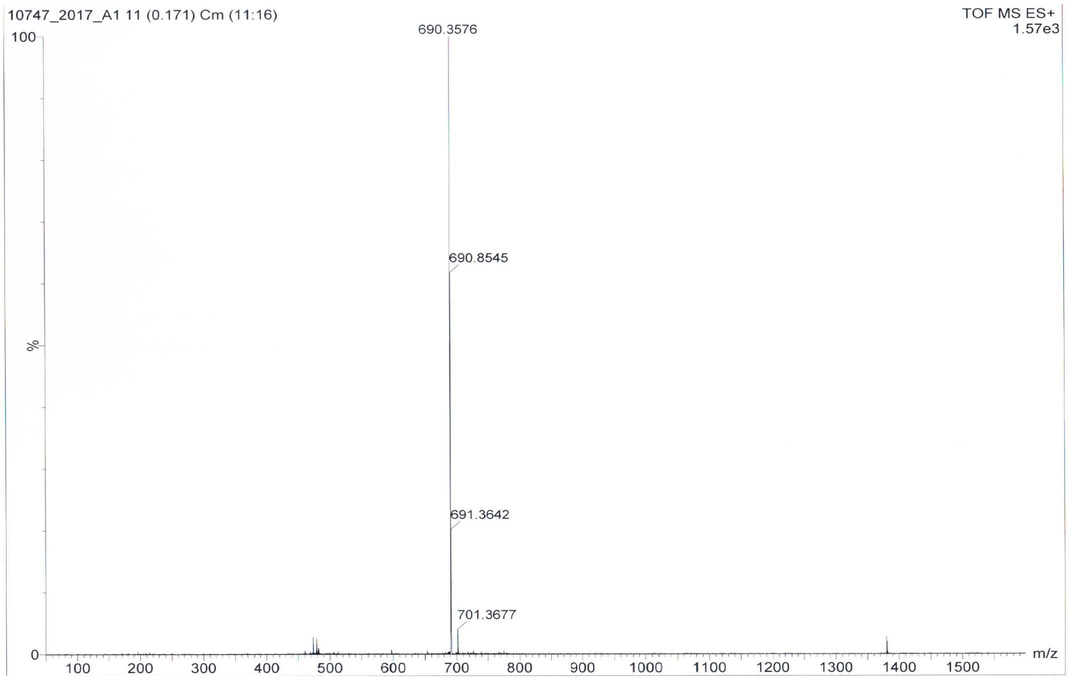

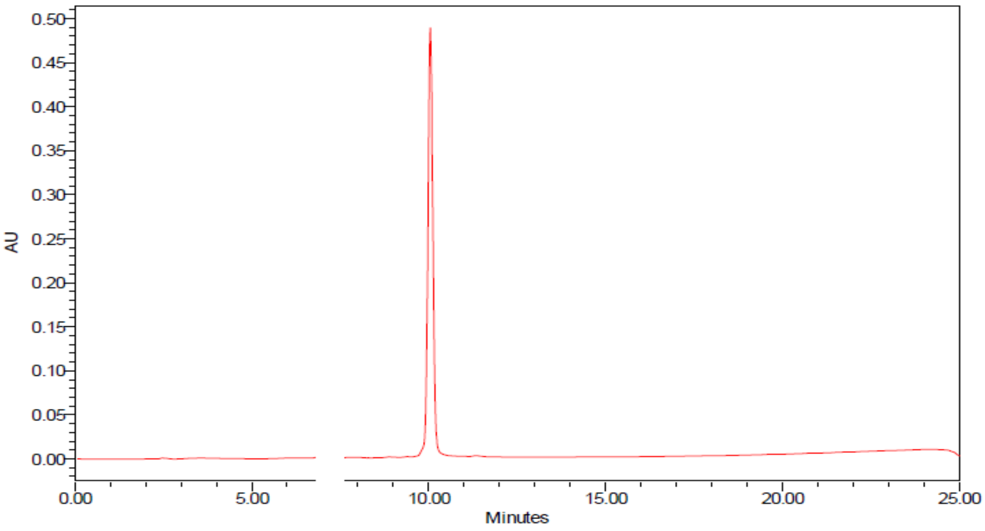

ACC-Ahx-Ala-Leu-Val-Ser-Arg-Gly-Lys-Lys(Dnp)-Gly

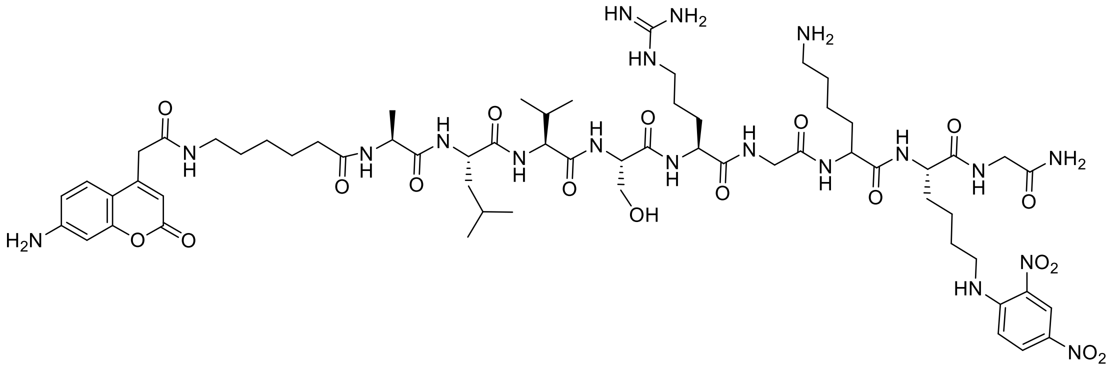

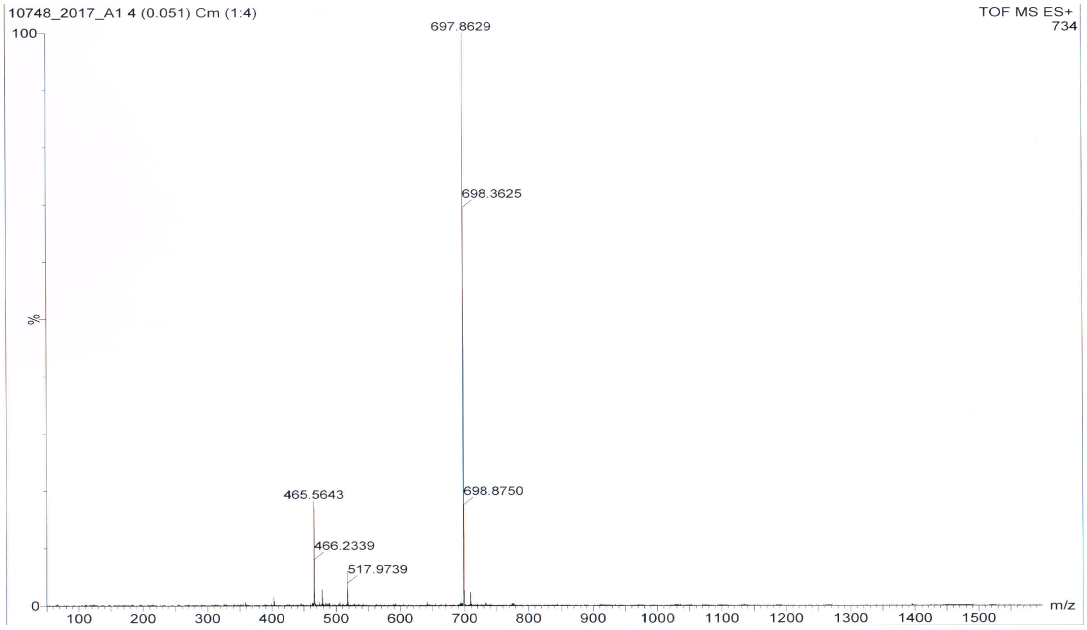

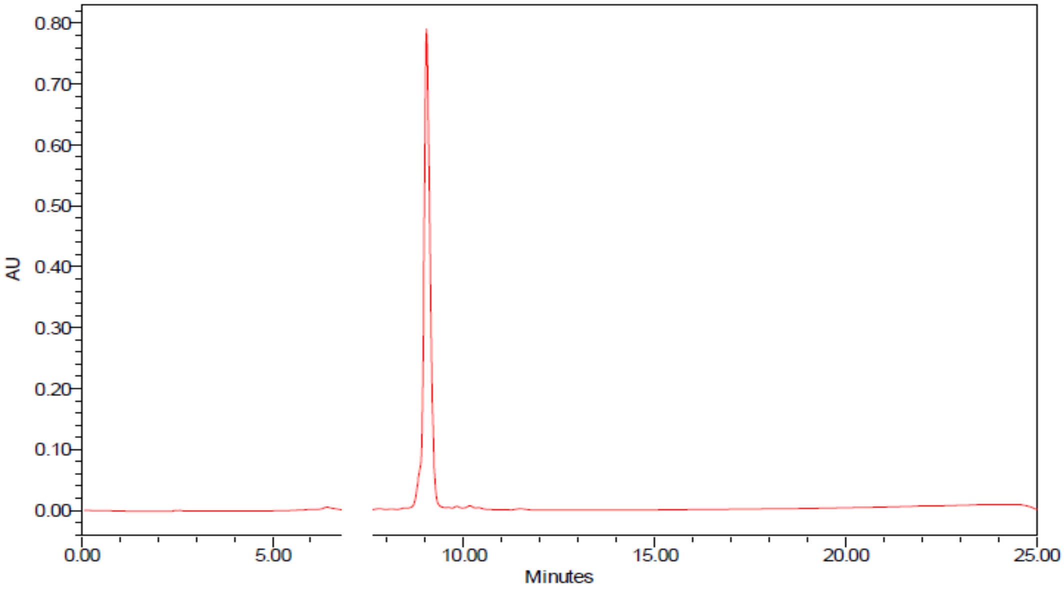

ACC-Ahx-Ala-Leu-Val-Ser-Arg-Gly-Nle-Lys(Dnp)-Gly

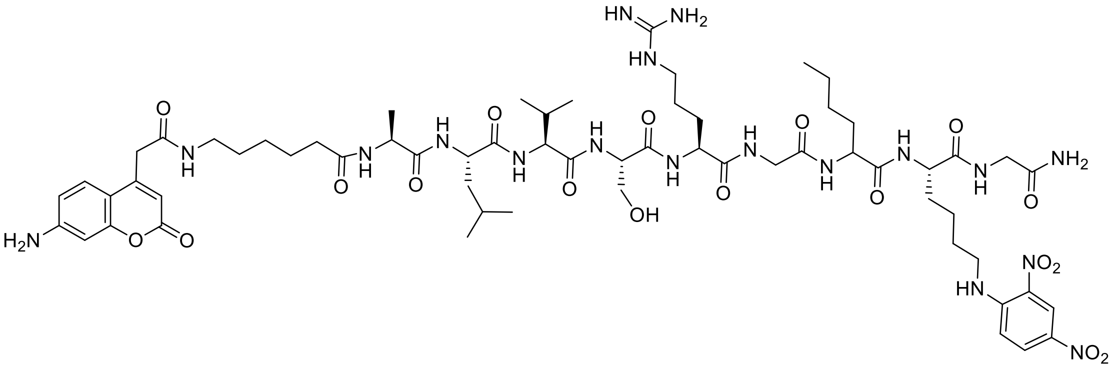

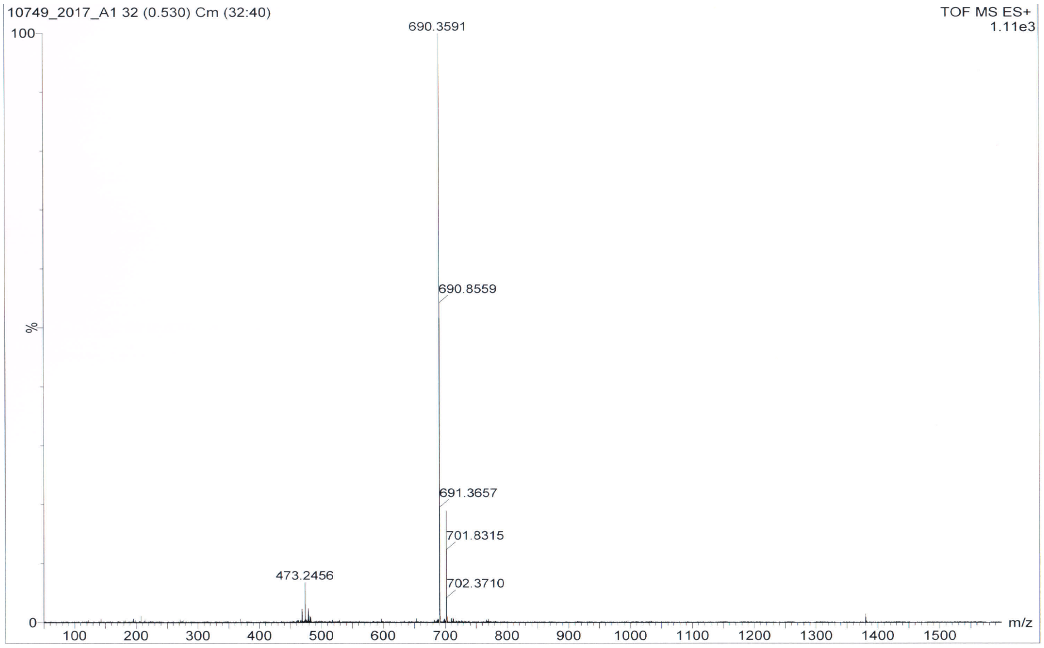

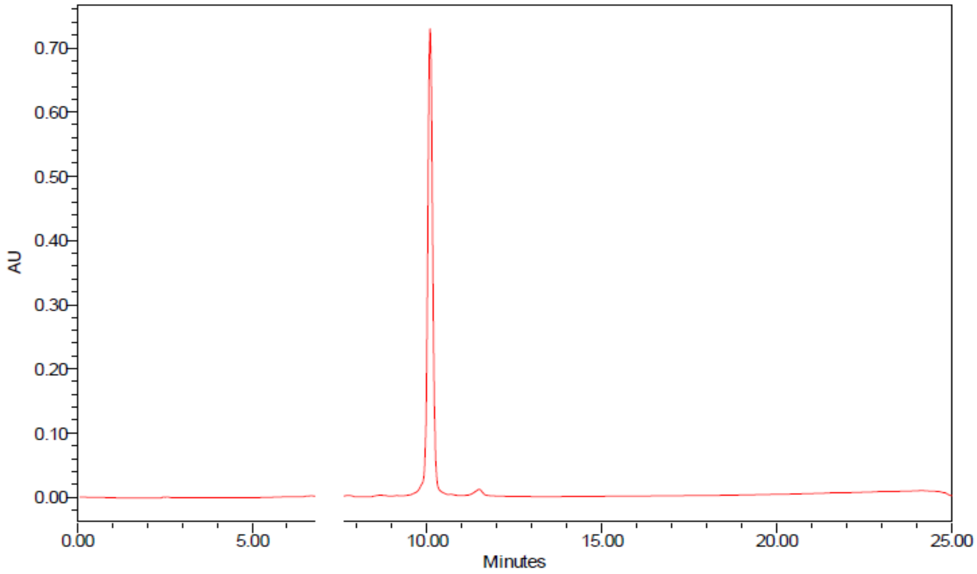

ACC-Ahx-Ala-Leu-Val-Ser-Arg-Gly-Phe-Lys(Dnp)-Gly

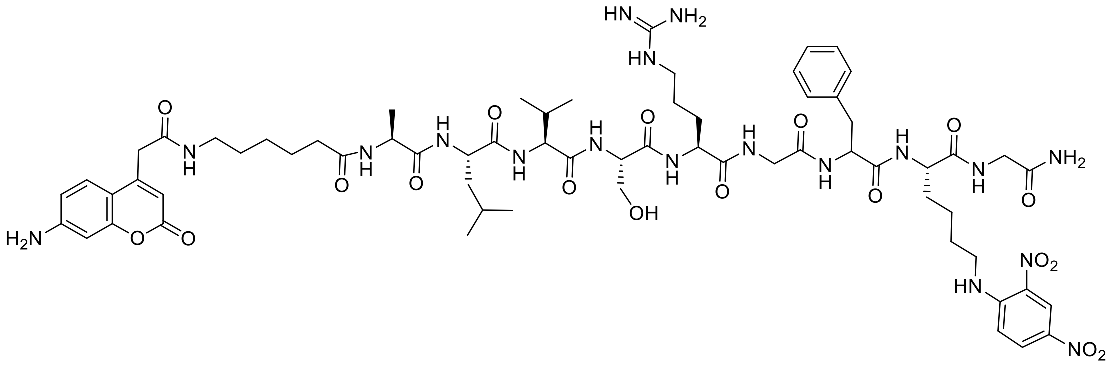

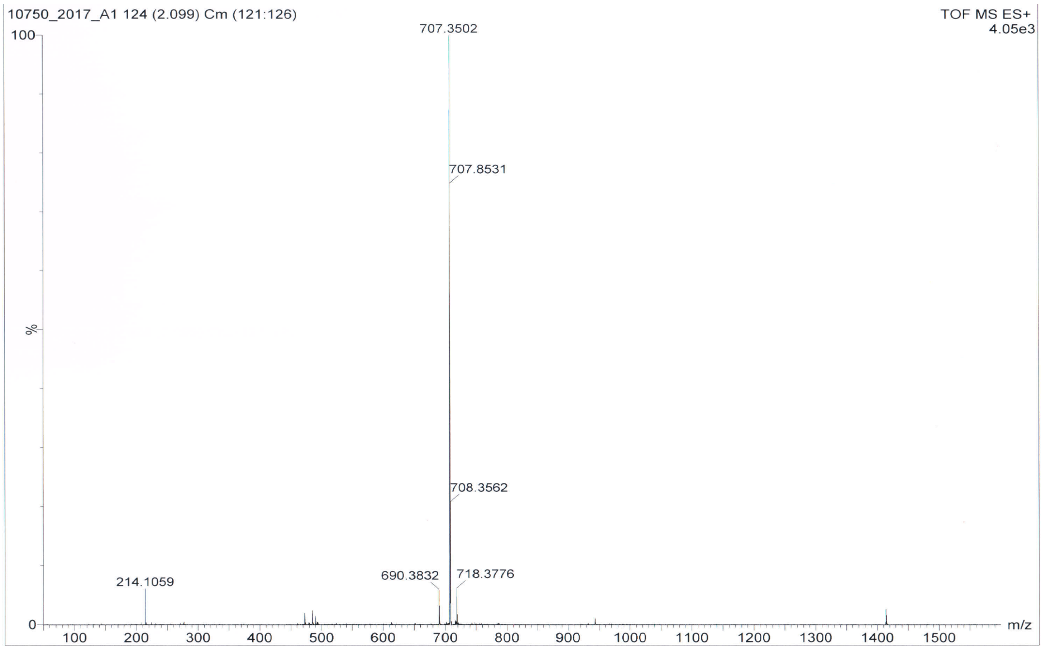

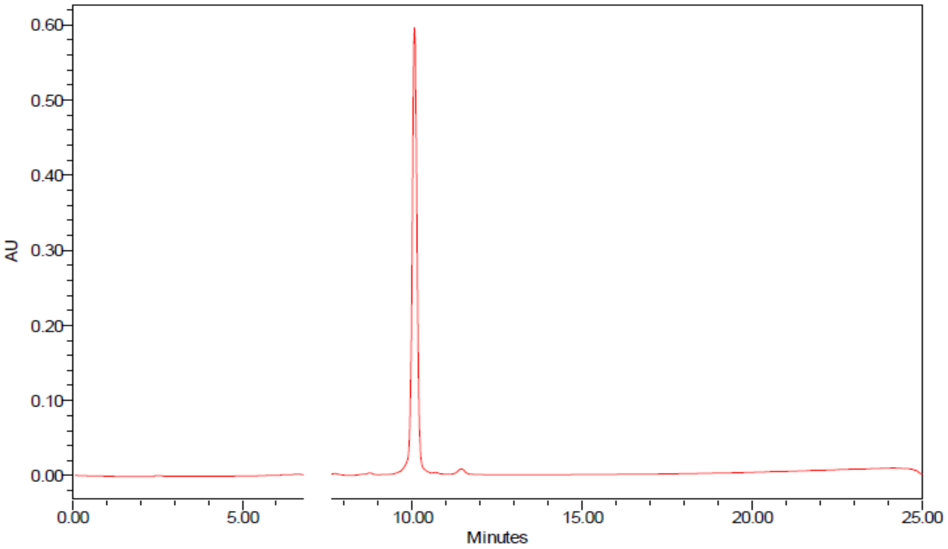

ACC-Ahx-Ala-Leu-Val-Ser-Arg-Gly-Pro-Lys(Dnp)-Gly

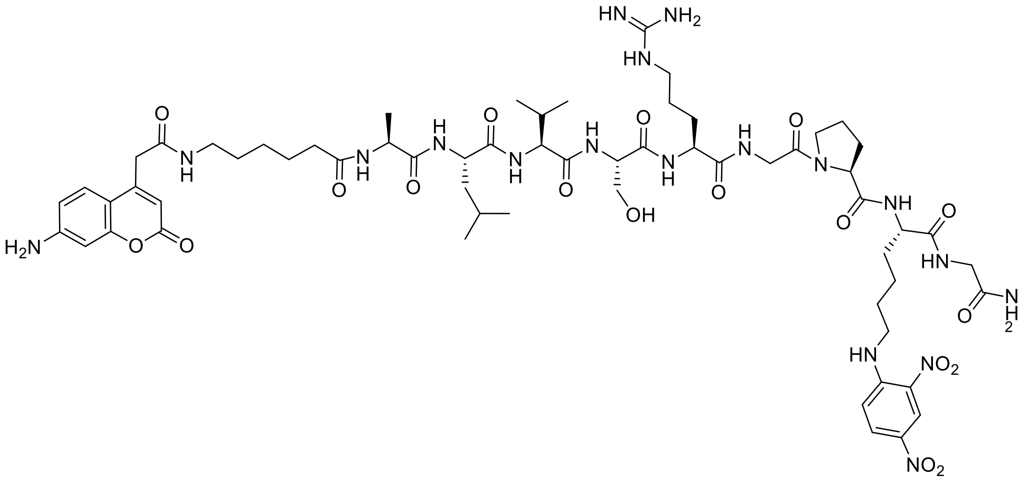

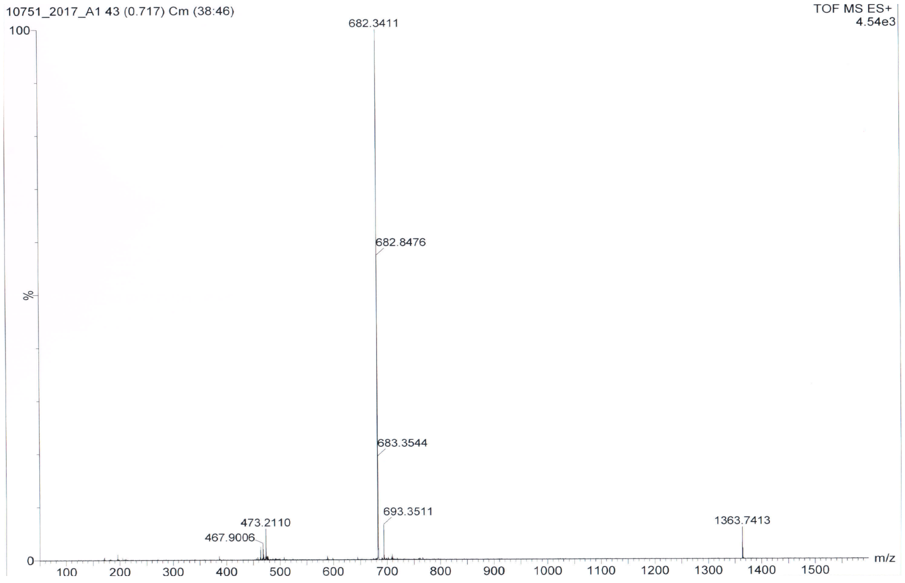

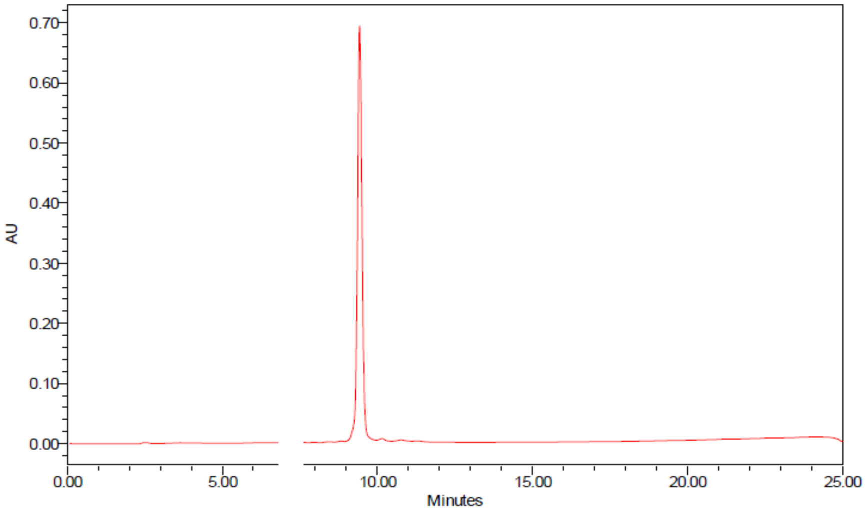

ACC-Ahx-Ala-Leu-Val-Ser-Arg-Gly-Ser-Lys(Dnp)-Gly

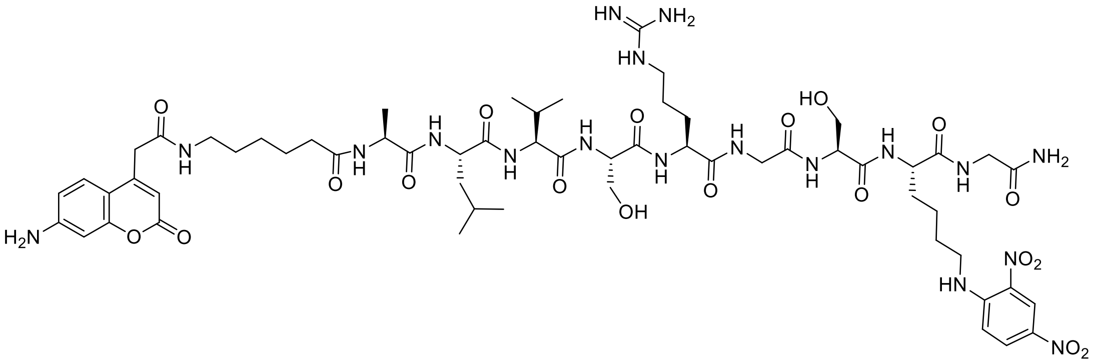

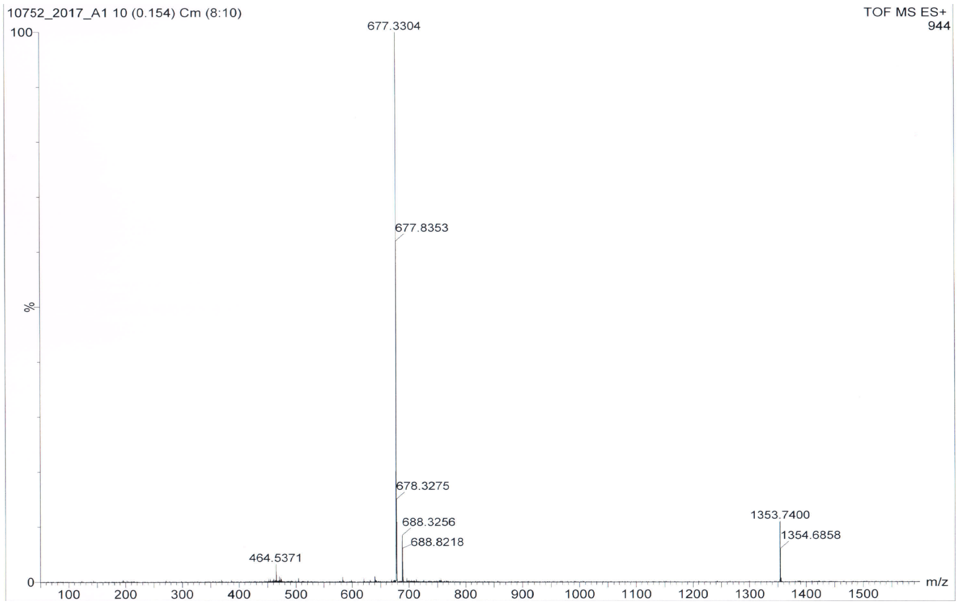

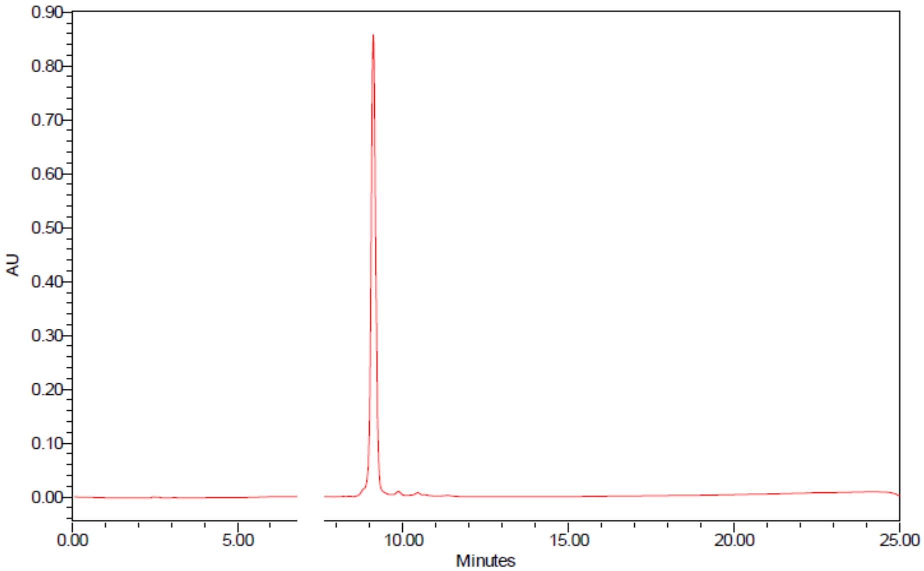

ACC-Ahx-Ala-Leu-Val-Ser-Arg-Gly-Thr-Lys(Dnp)-Gly

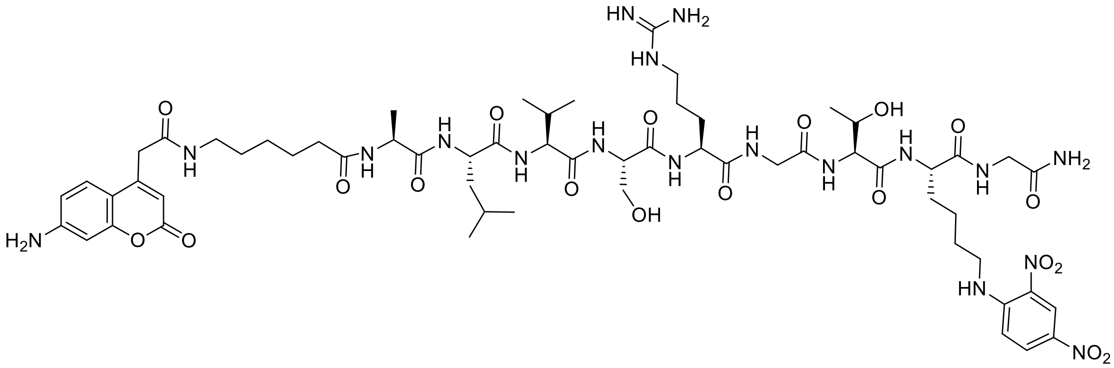

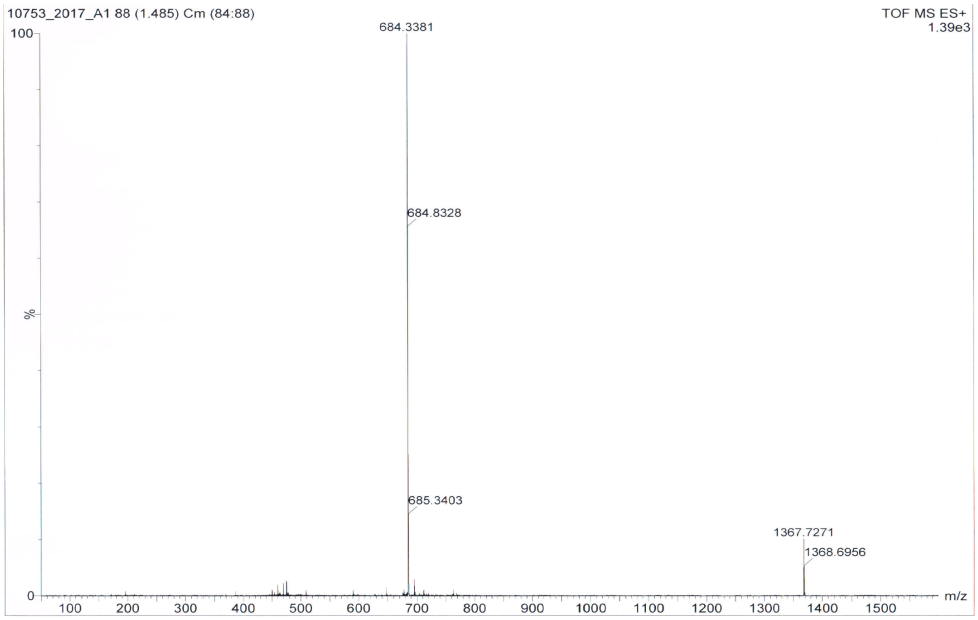

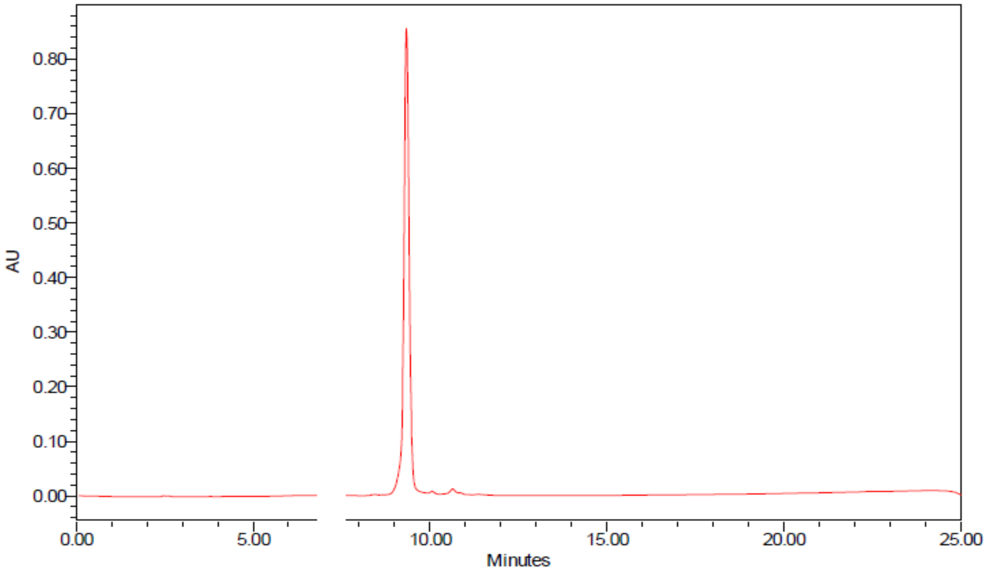

ACC-Ahx-Ala-Leu-Val-Ser-Arg-Gly-Trp-Lys(Dnp)-Gly

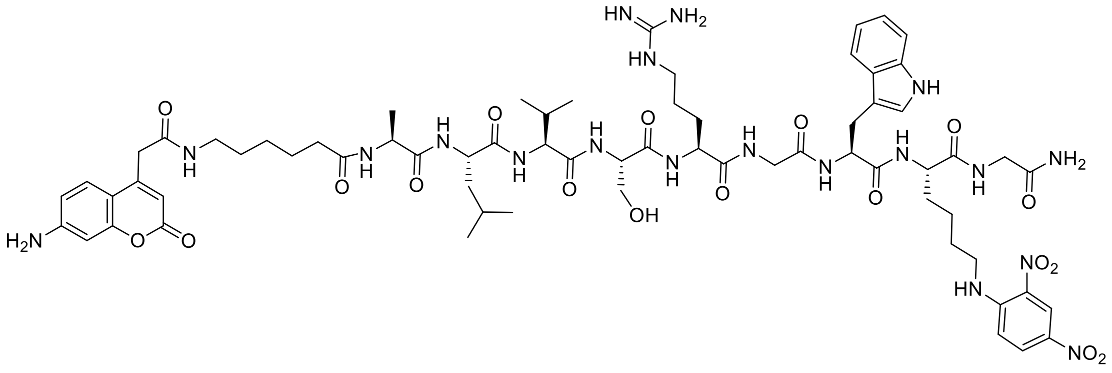

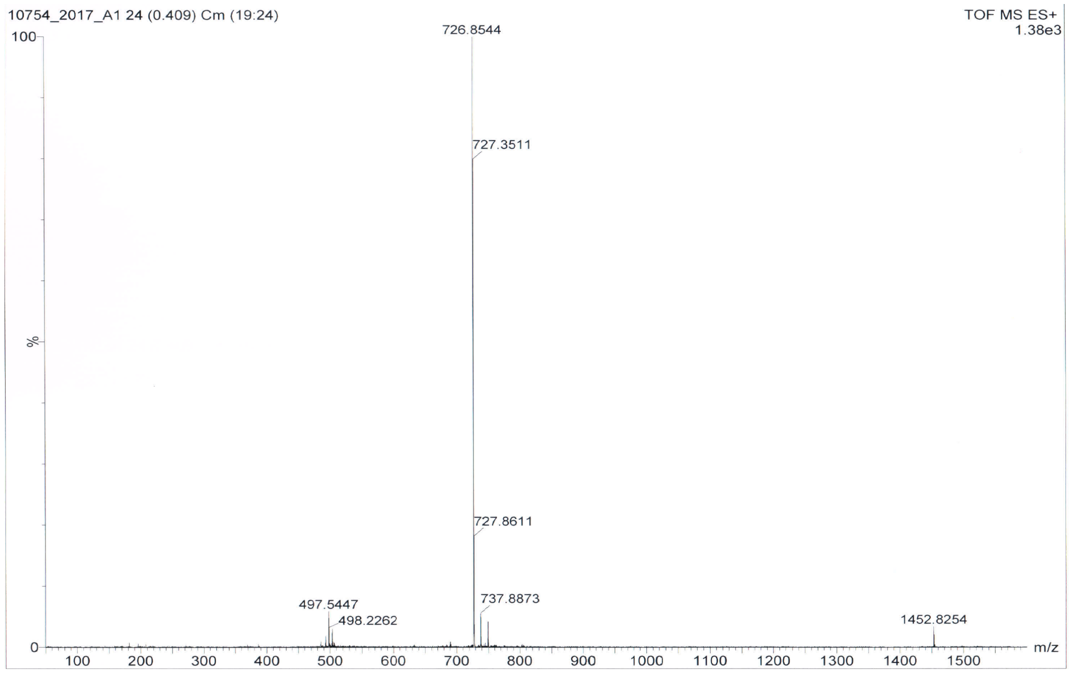

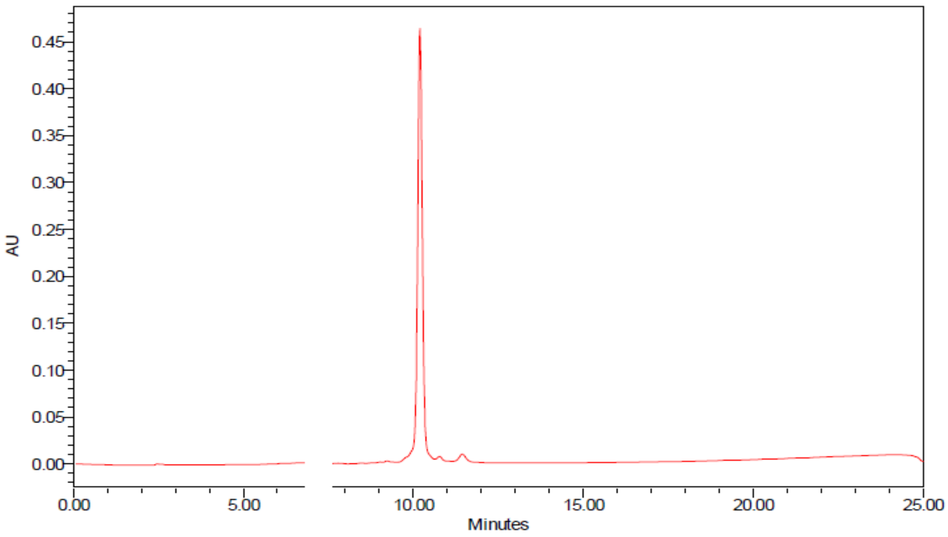

ACC-Ahx-Ala-Leu-Val-Ser-Arg-Gly-Tyr-Lys(Dnp)-Gly

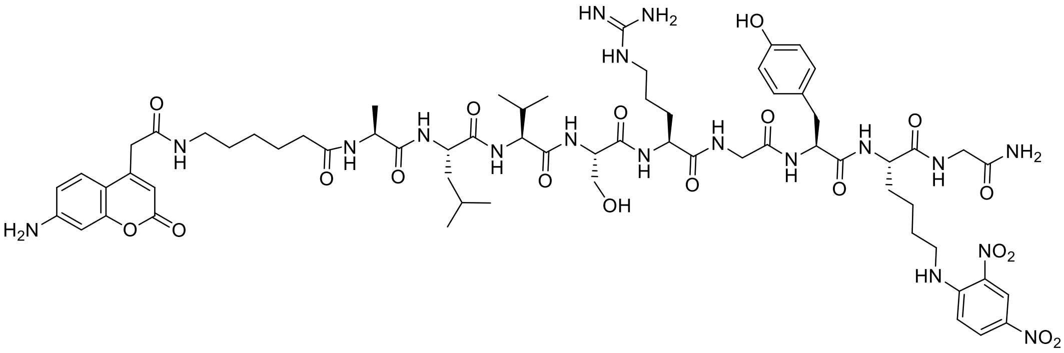

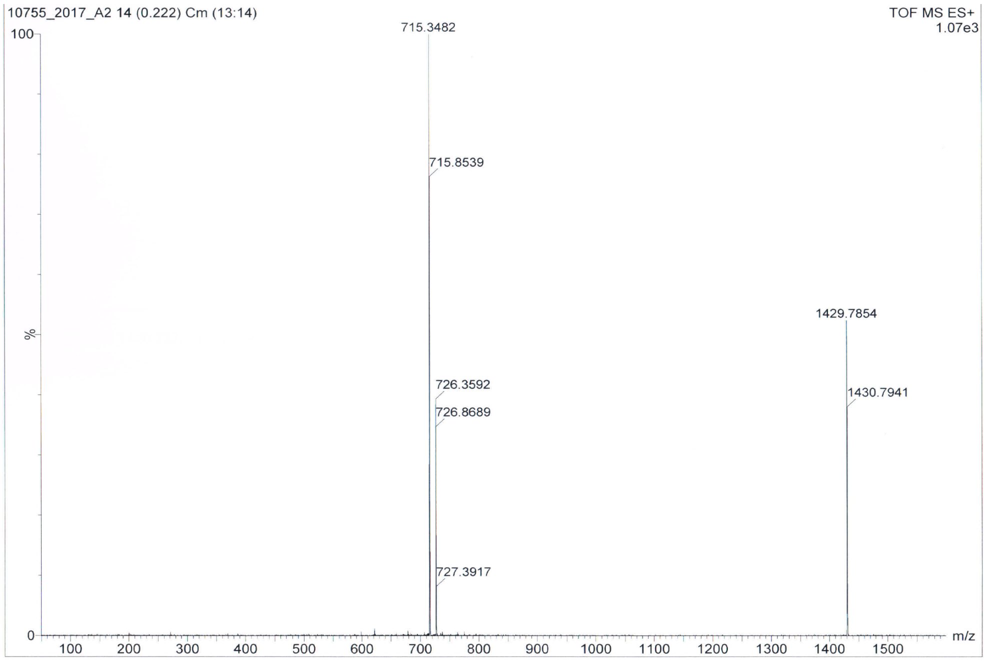

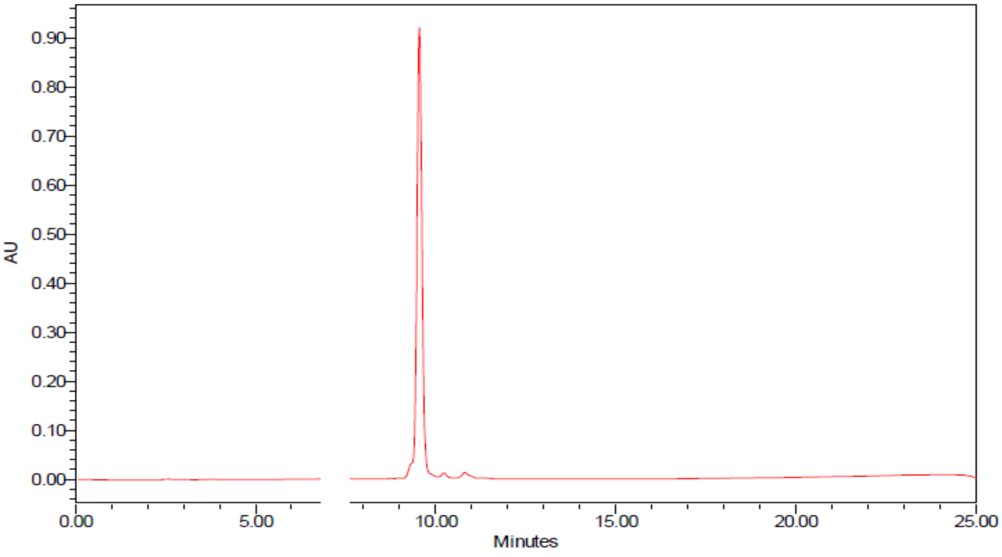

ACC-Ahx-Ala-Leu-Val-Ser-Arg-Gly-Val-Lys(Dnp)-Gly

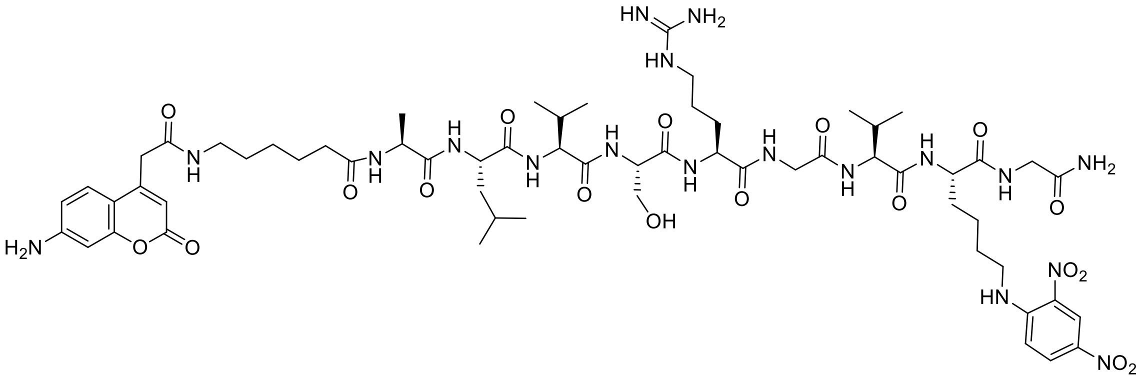

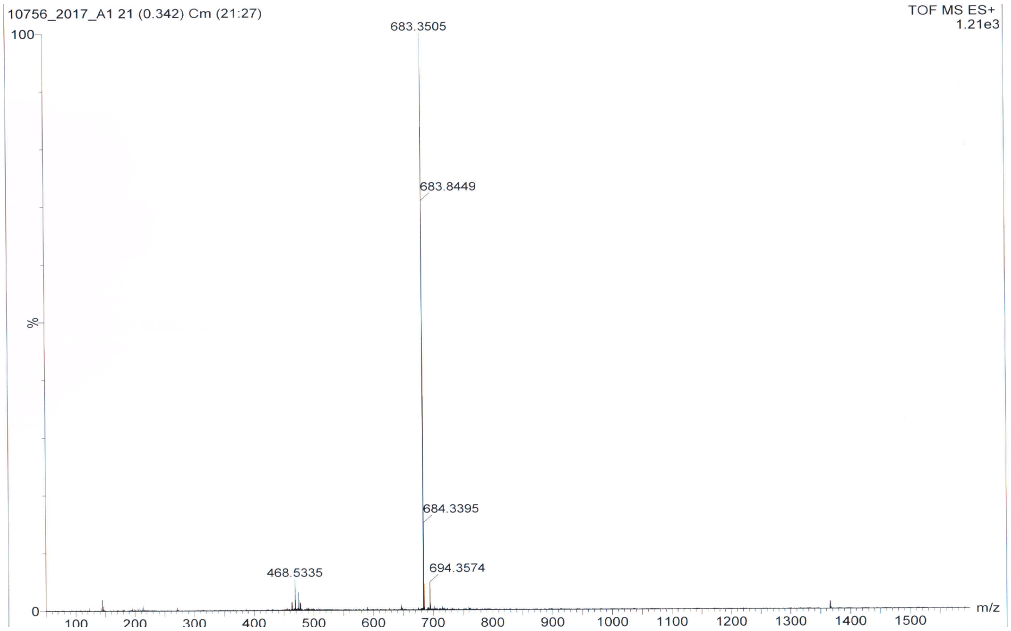

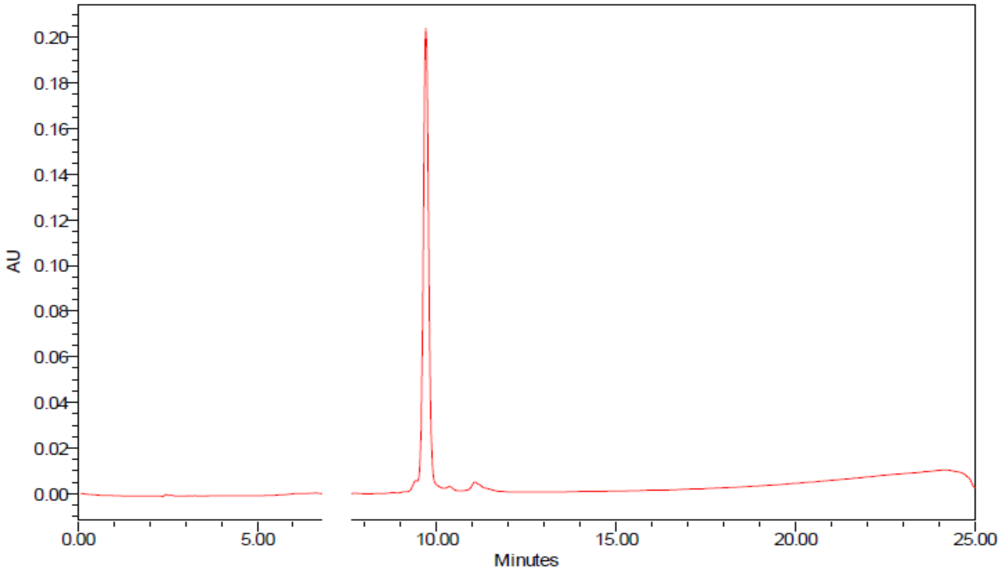

ACC-Ahx-Ala-Leu-Val-Ser-Arg-Gly-Met-Lys(Dnp)-Gly

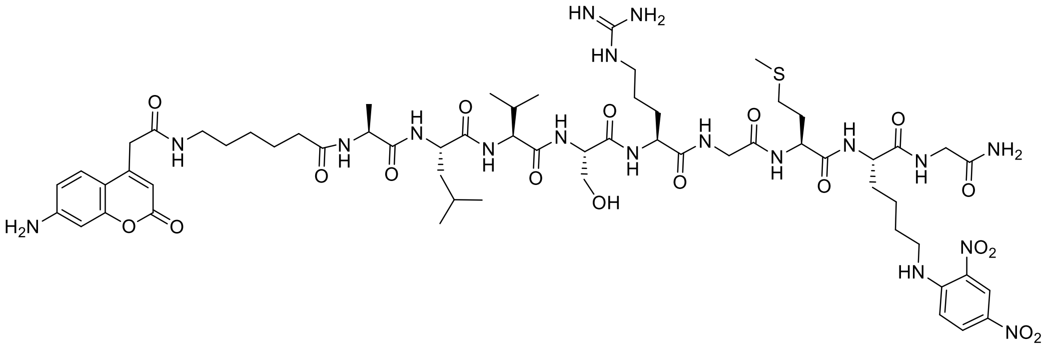

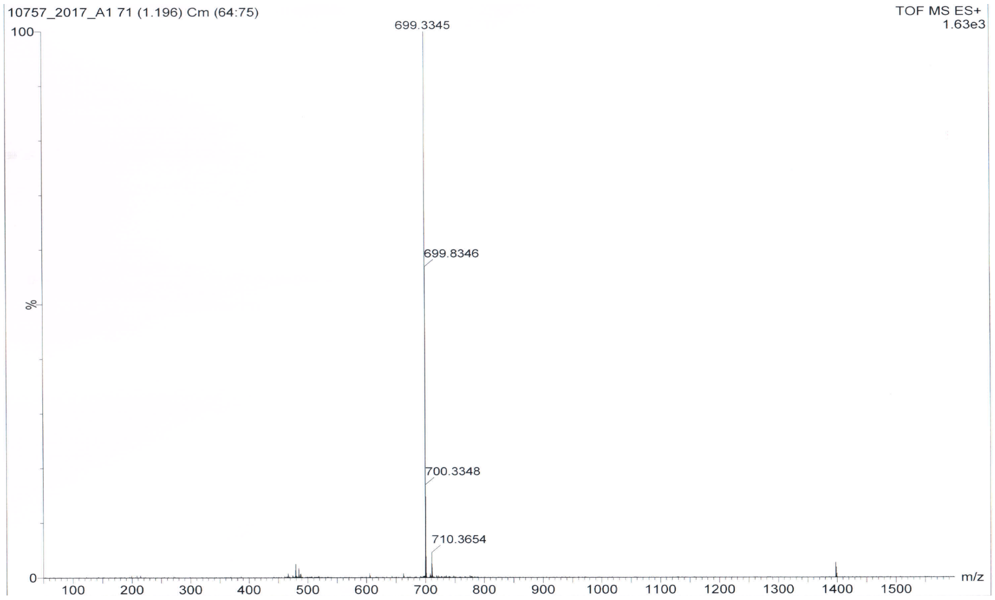

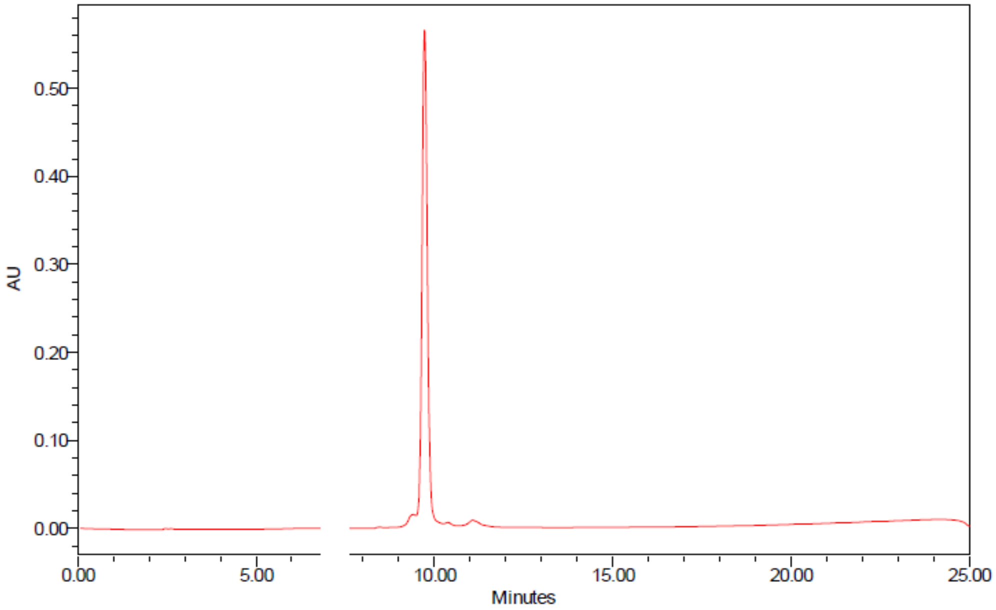

ACC-Ahx-Ala-Leu-Val-Ser-Arg-Gly-hSer-Lys(Dnp)-Gly

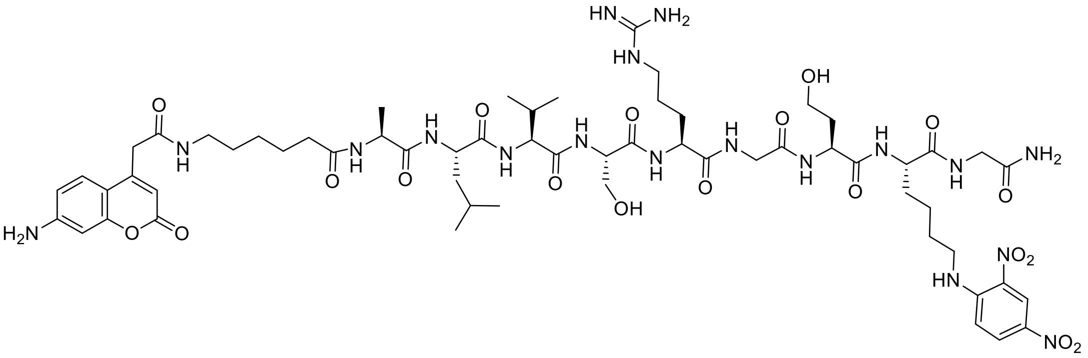

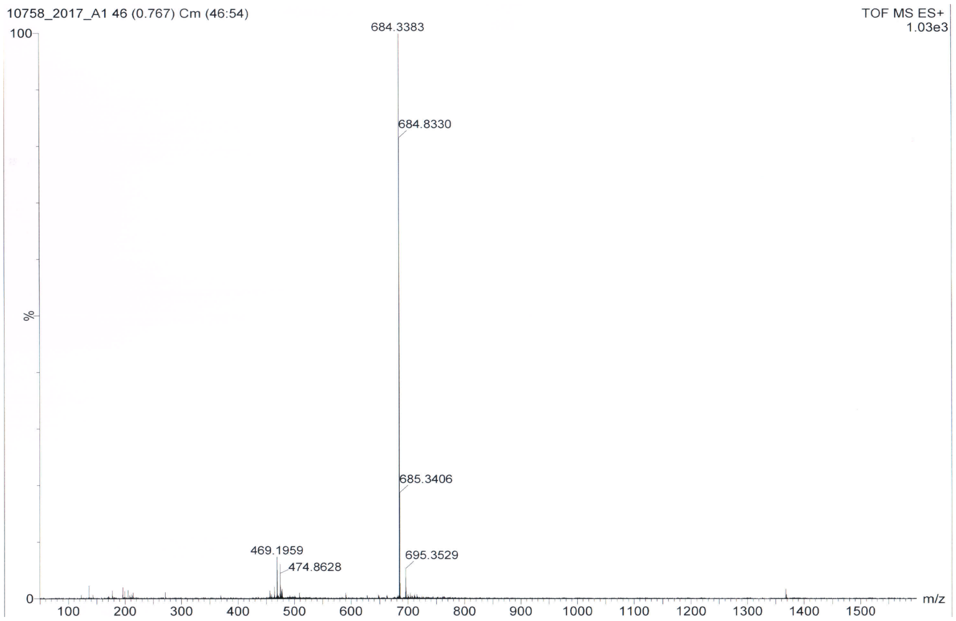

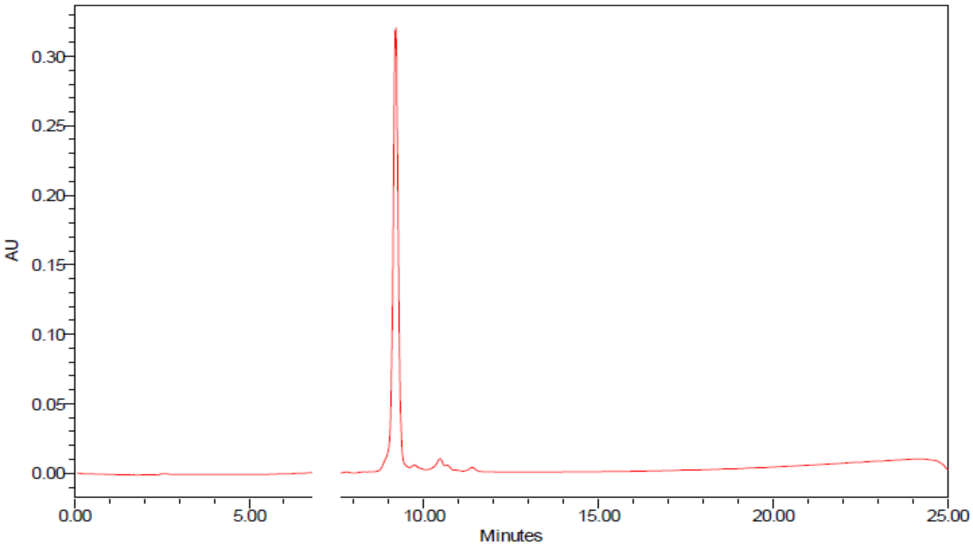

ACC-Ahx-Ala-Leu-Val-Ser-Arg-Gly-Abu-Lys(Dnp)-Gly

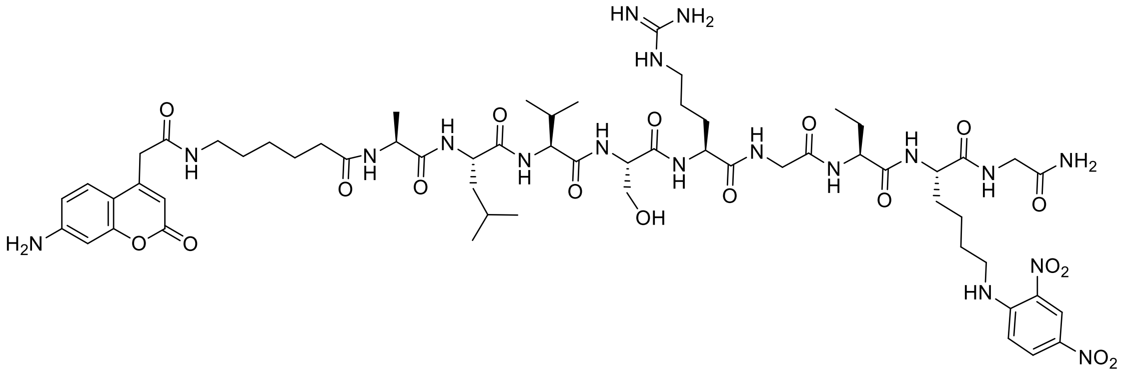

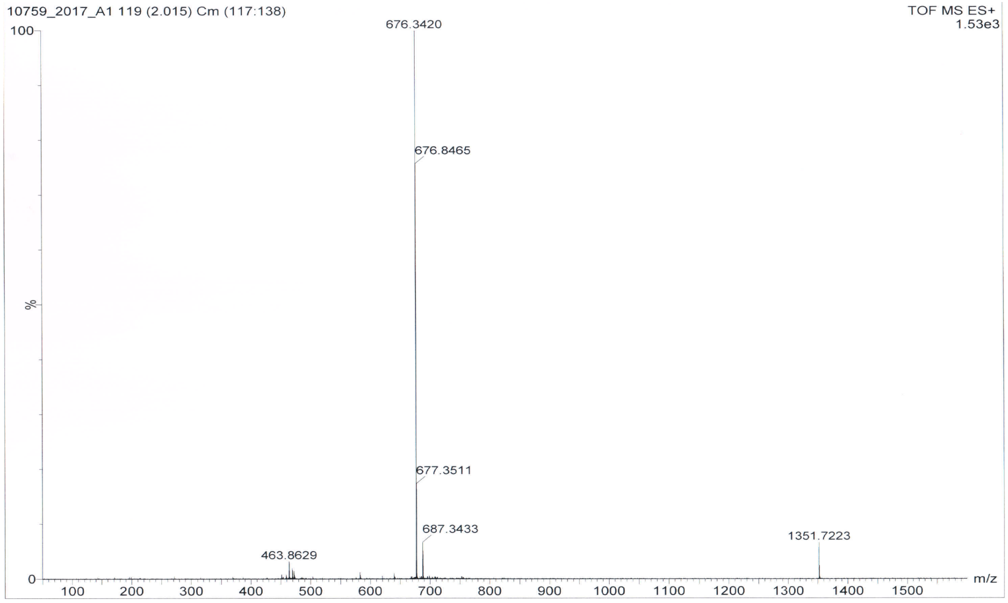

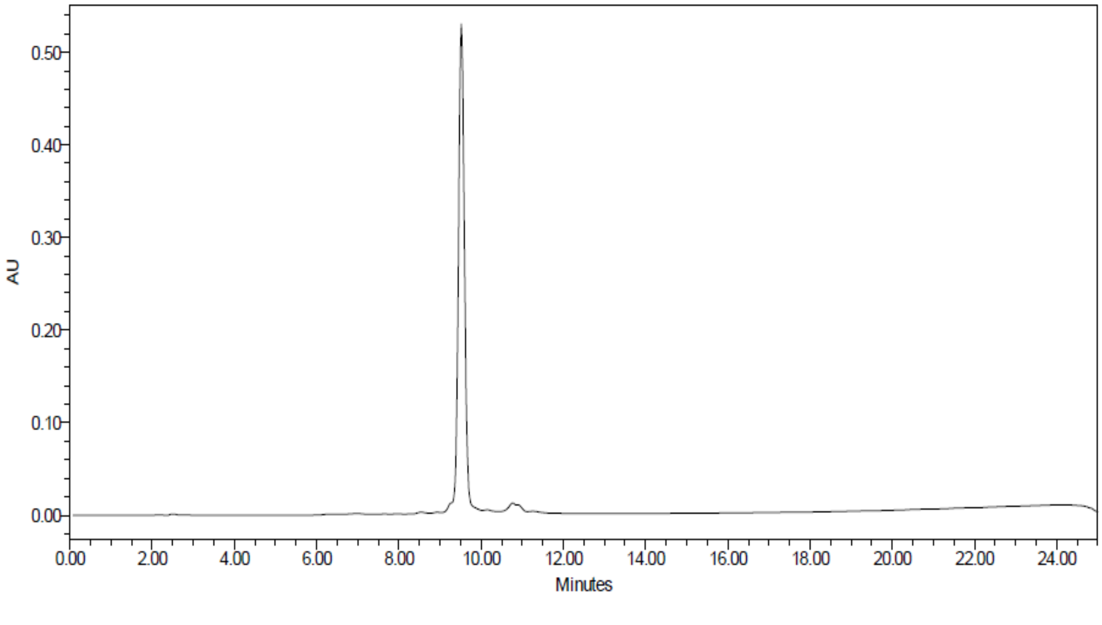

ACC-Ahx-Ala-Leu-Val-Ser-Arg-Gly- β-Ala-Lys(Dnp)-Gly

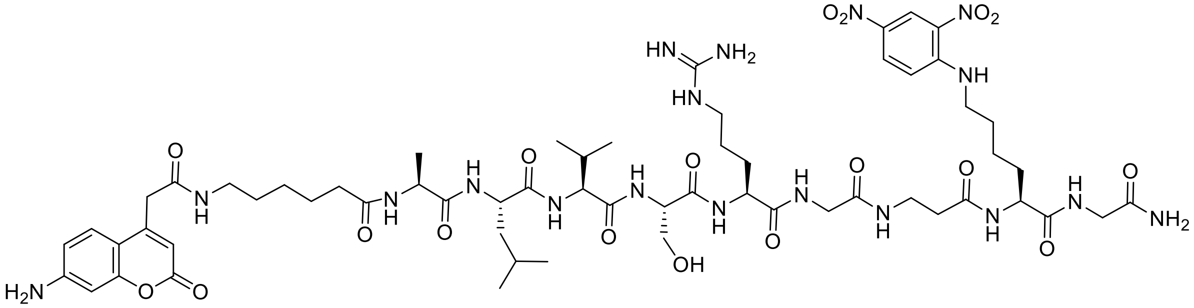

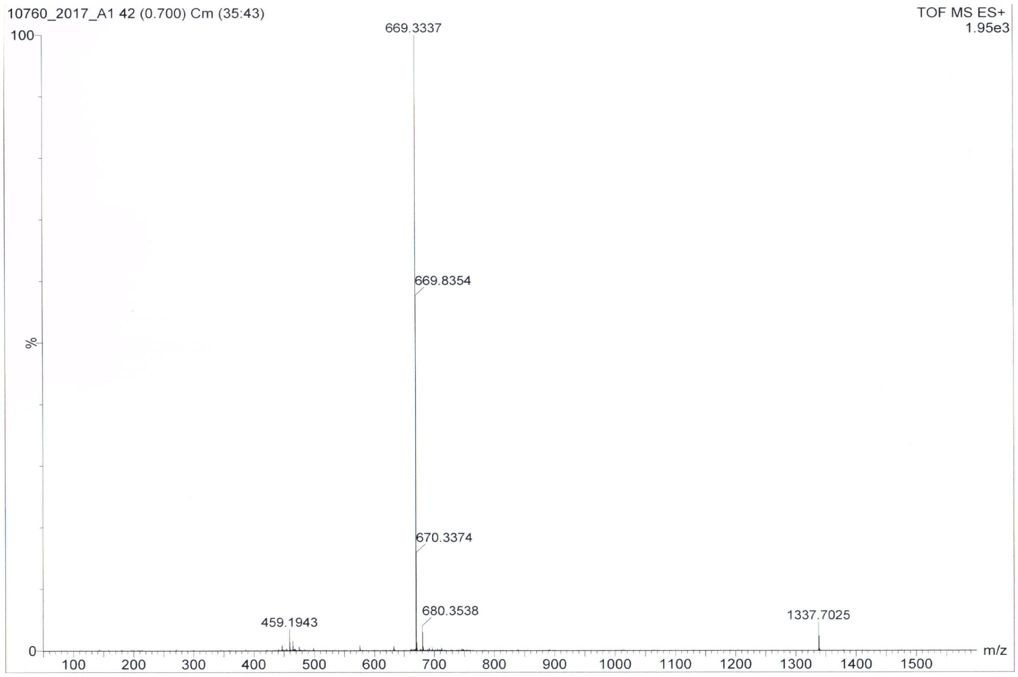

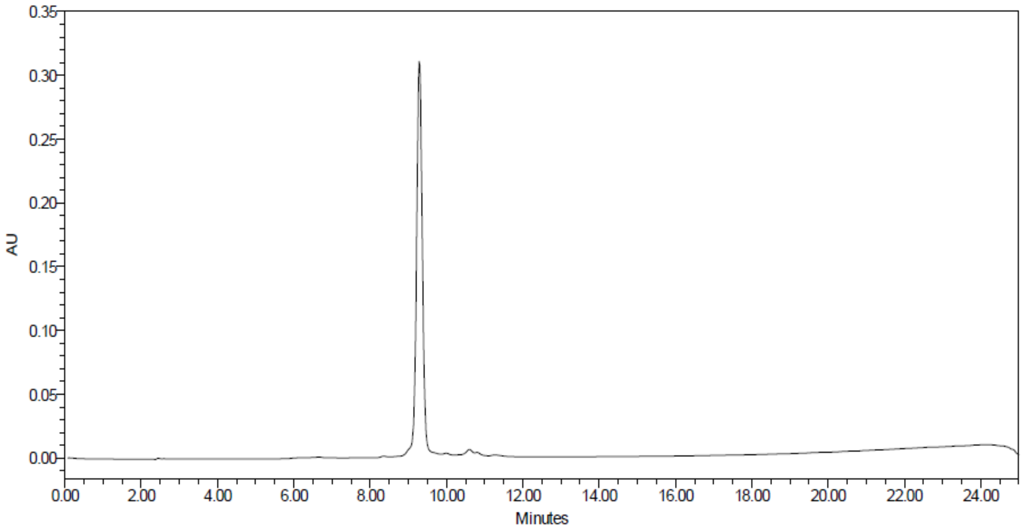

ACC-Ahx-Ala-Leu-Val-Ser-Arg-Gly-Tle-Lys(Dnp)-Gly

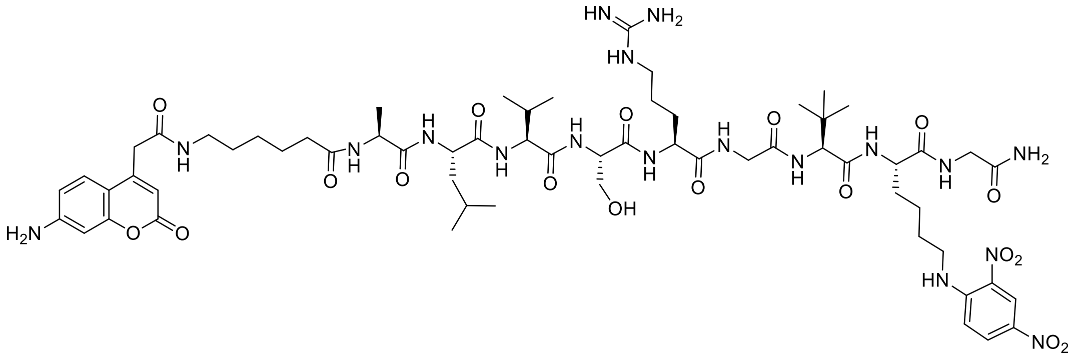

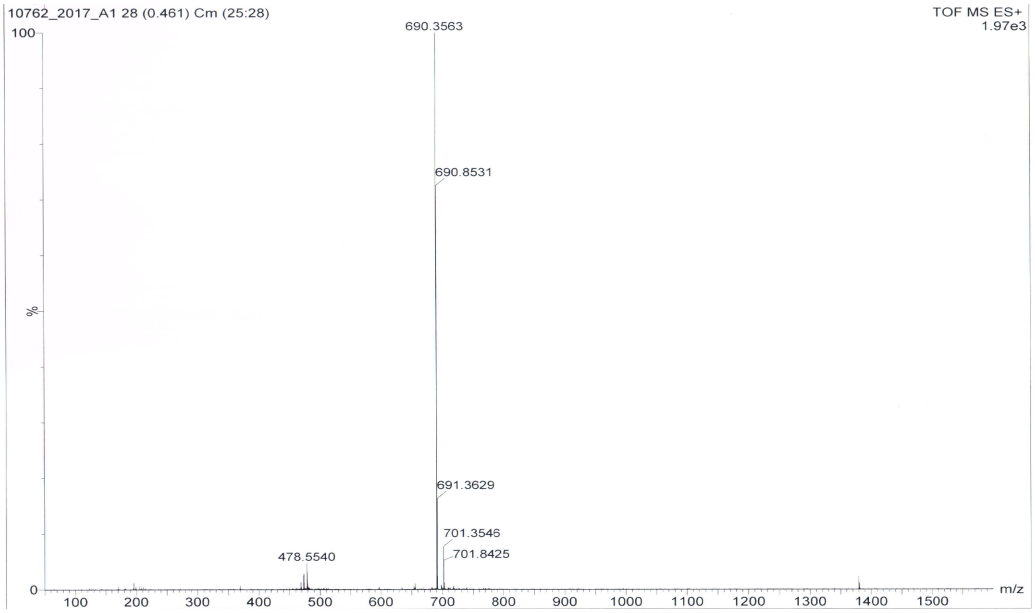

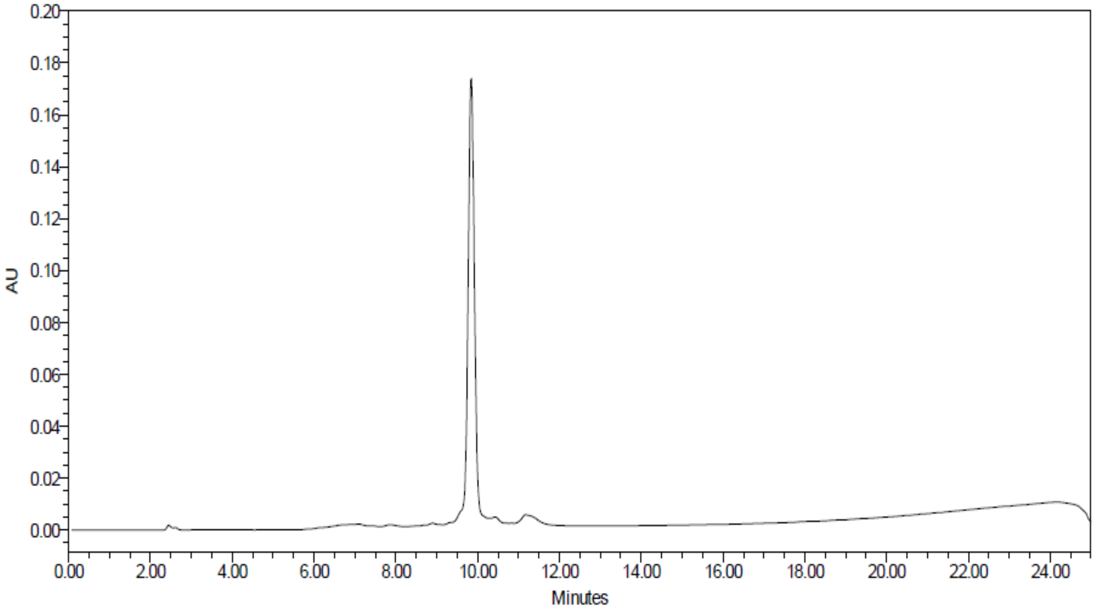

ACC-Ahx-Ala-Leu-Val-Ser-Arg-Gly-Nva-Lys(Dnp)-Gly

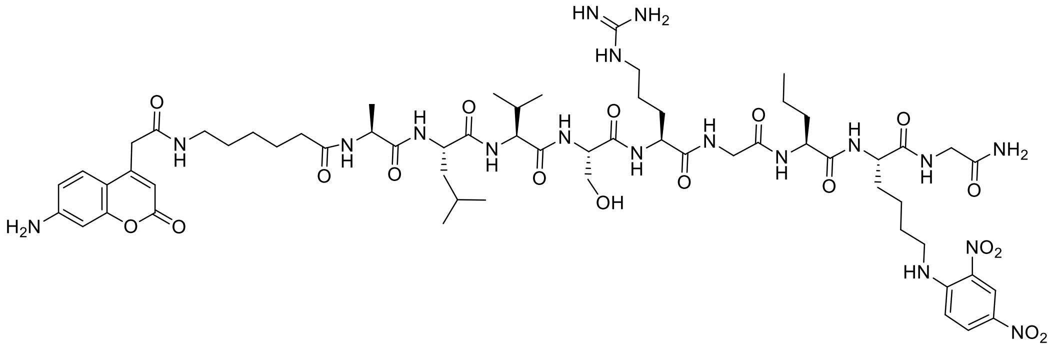

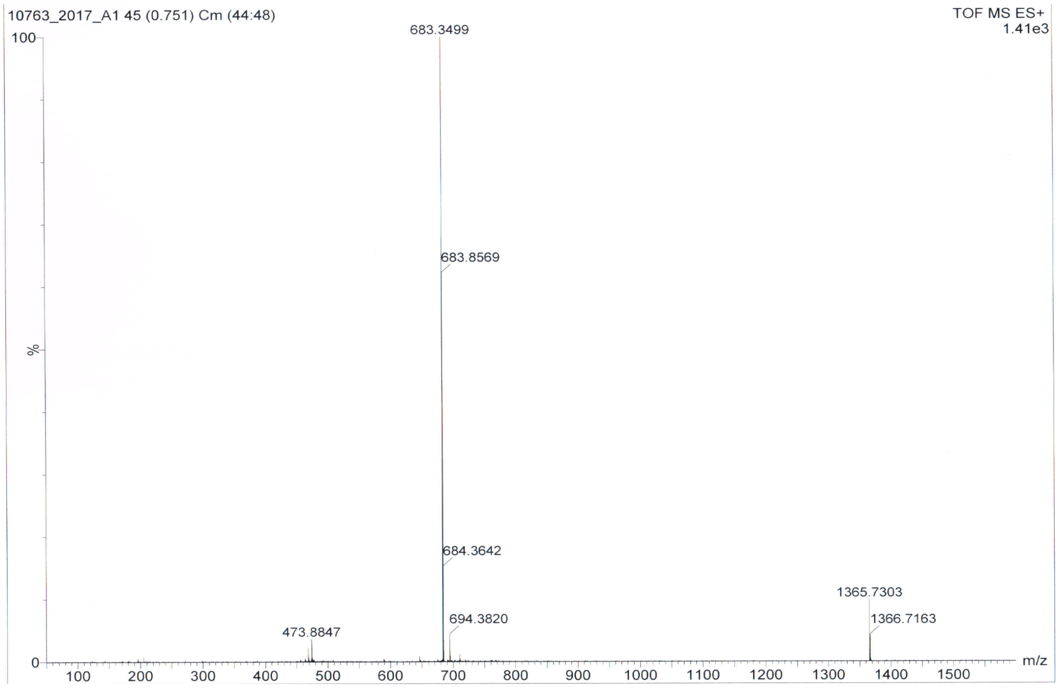

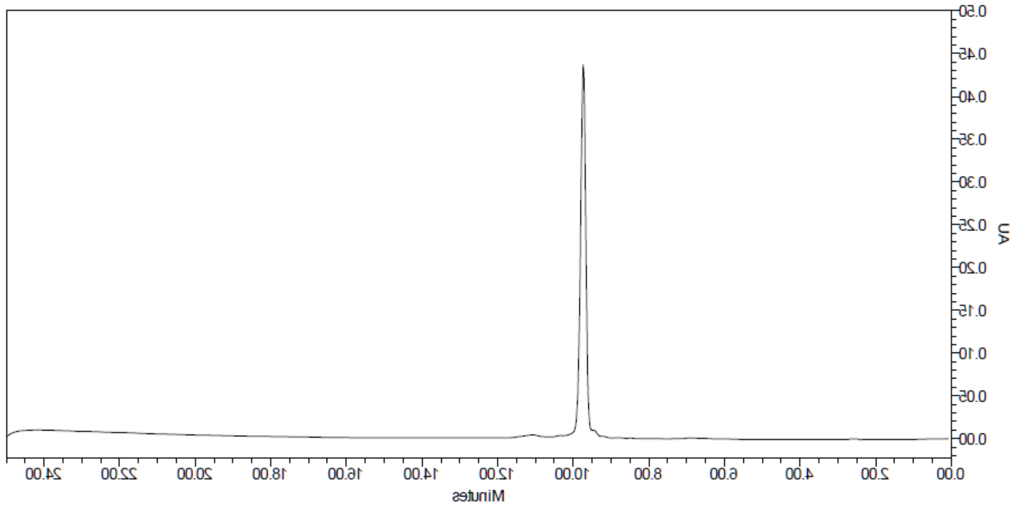

ACC-Ahx-Ala-Leu-Val-Ser-Arg-Leu-Ala-Lys(Dnp)-Gly

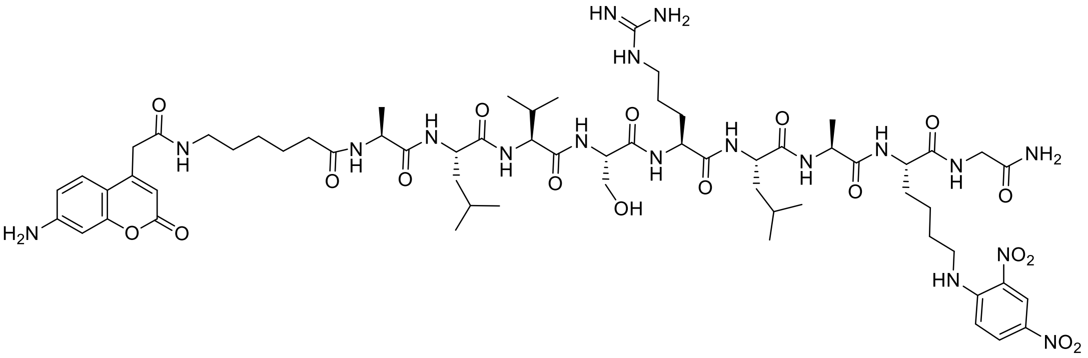

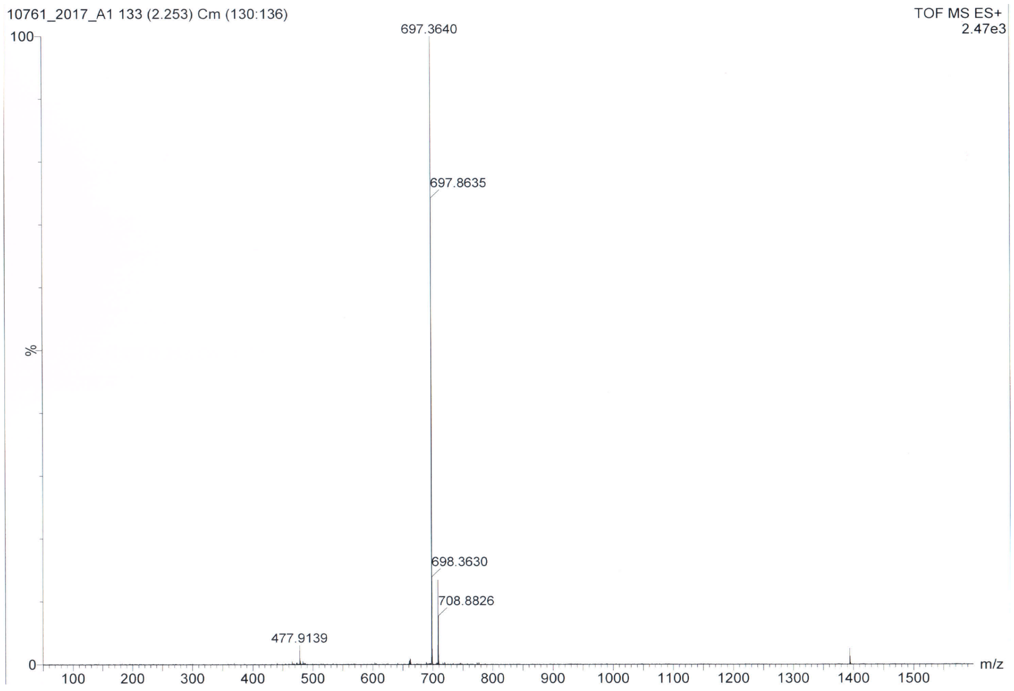

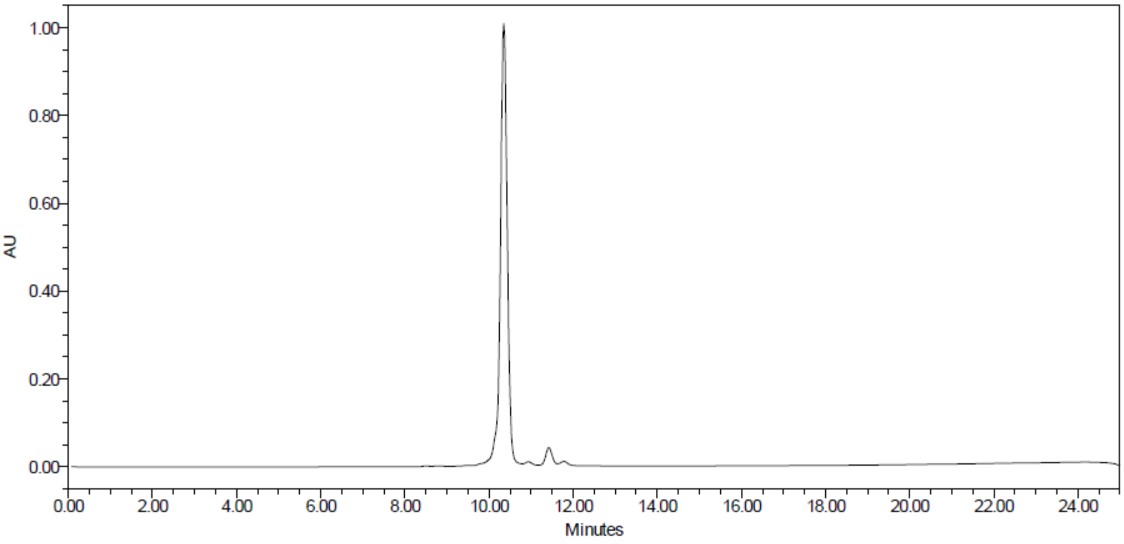

Supplement: Supplementary file 1 — Supplementary Dataset 1 [file 41598_2018_34476_MOESM1_ESM.pdf]
